# Full-length huntingtin is palmitoylated at multiple sites and post-translationally myristoylated following caspase-cleavage

**DOI:** 10.3389/fphys.2023.1086112

**Published:** 2023-01-13

**Authors:** Fanny L. Lemarié, Shaun S. Sanders, Yen Nguyen, Dale D. O. Martin, Michael R. Hayden

**Affiliations:** Centre for Molecular Medicine and Therapeutics, BC Children’s Hospital Research Institute, University of British Columbia, Vancouver, BC, Canada

**Keywords:** huntingtin, HTT, huntington disease, fatty acylation, myristoylation and palmitoylation, post-translational modification (PTM)

## Abstract

**Introduction:** Huntington disease is an autosomal dominant neurodegenerative disorder which is caused by a CAG repeat expansion in the HTT gene that codes for an elongated polyglutamine tract in the huntingtin (HTT) protein. Huntingtin is subjected to multiple post-translational modifications which regulate its cellular functions and degradation. We have previously identified a palmitoylation site at cysteine 214 (C214), catalyzed by the enzymes ZDHHC17 and ZDHHC13. Reduced palmitoylation level of mutant huntingtin is linked to toxicity and loss of function. Moreover, we have described N-terminal myristoylation by the N-myristoyltransferases of a short fragment of huntingtin (HTT553-586) at glycine 553 (G553) following proteolysis at aspartate 552 (D552).

**Results:** Here, we show that huntingtin is palmitoylated at numerous cysteines: C105, C433, C3134 and C3144. In addition, we confirm that full-length huntingtin is cleaved at D552 and post-translationally myristoylated at G553. Importantly, blocking caspase cleavage at the critical and pathogenic aspartate 586 (D586) significantly increases posttranslational myristoylation of huntingtin. In turn, myristoylation of huntingtin promotes the co-interaction between C-terminal and N-terminal huntingtin fragments, which is also protective.

**Discussion:** This suggests that the protective effect of inhibiting caspase-cleavage at D586 may be mediated through post-translational myristoylation of huntingtin at G553.

## 1 Introduction

Huntington disease (HD) is an autosomal dominant neurodegenerative disorder characterized clinically by behavioral changes and a progressive deterioration of motor function and cognitive ability that ultimately leads to death (Ghosh and Tabrizi, 2018). The genetic cause of HD is a CAG trinucleotide repeat expansion (>35 repeats) in the huntingtin gene (*HTT*) resulting in an abnormally long polyglutamine (polyQ) stretch near the N-terminal region of the huntingtin (HTT) protein. Wild-type HTT is a 3,144 amino acid long scaffold protein that plays a critical role in brain development, neuronal health and connectivity (Harjes and Wanker, 2003; Reiner et al., 2003; McKinstry et al., 2014; Dragatsis et al., 2018; Mehler et al., 2019; Burrus et al., 2020). Through interactions with its numerous protein partners, HTT is involved in many cellular processes, including endocytosis, vesicle/organelle transport and recycling, autophagy, and DNA transcription (Ochaba et al., 2014; Saudou and Humbert, 2016; Maiuri et al., 2017).

The wild-type HTT protein undergoes a large variety of post-translational modifications (PTMs) including phosphorylation (Humbert et al., 2002; Warby et al., 2005; Schilling et al., 2006; Aiken et al., 2009; Watkin et al., 2014), ubiquitination (Kalchman et al., 1996), SUMOylation (Steffan et al., 2004), acetylation (Jeong et al., 2009), proteolysis (Graham et al., 2006) and fatty acylation (Yanai et al., 2006; Martin et al., 2014). The role of the identified PTMs has mainly been studied in relation to the toxicity and aggregation of mutant HTT, but their effects on wild-type HTT functions remain elusive.

PTMs likely regulate different aspects of wild-type HTT scaffolding function, including subcellular localization and protein-protein interactions (Ehrnhoefer et al., 2011). Many PTMs of HTT are altered in the presence of the polyQ expansion, impacting mutant HTT clearance and aggregation which, in turn, modulate mutant HTT toxicity (Atwal et al., 2007; 2011; Caron et al., 2013; Kratter et al., 2016; Cariulo et al., 2017; Lontay et al., 2020).

S-palmitoylation of proteins refers to the reversible addition of long-chain fatty acids, commonly the 16-carbon palmitic acid, onto a cysteine residue *via* a thioester bond (Martin B. R et al., 2011). This PTM increases the hydrophobicity of proteins and plays a key role in protein trafficking, stability, membrane association and protein-protein interactions (Fukata and Fukata, 2010). We have initially described the palmitoylation of HTT at cysteine 214 (C214) by ZDHHC17 and 13 (also known as HIP14 and HIP14L) (Yanai et al., 2006). Mutant HTT is less palmitoylated compared to wild-type HTT in multiple experimental models of HD (Yanai et al., 2006; Lemarié et al., 2021). Transient expression of mutant HTT carrying a palmitoylation-resistant mutation (C214 to serine, C214S) in immortalized cell lines and primary neurons leads to increased mutant HTT aggregation and nuclear inclusion formation, cell death, and susceptibility to excitotoxicity (Yanai et al., 2006). Importantly, modulating palmitoylation by inhibiting depalmitoylating acyl-protein thioesterases (APTs) is protective in HD cells (immortalized cell lines, primary neurons, iPSC-derived neurons) and in the *Hdh* ^CAG140/+^ knock-in mouse model (Lemarié et al., 2021; Virlogeux et al., 2021).

N-myristoylation corresponds to the irreversible addition of the 14-carbon myristic acid *via* a covalent amide bond to the N-terminal glycine residue of proteins, catalyzed by two N-myristoyltransferases (NMTs) (Martin D. D. O et al., 2011; Giglione and Meinnel, 2022). Myristoylation occurs either co-translationally, following the removal of the initiator methionine residue, or post-translationally, when a previously internal glycine residue becomes exposed by proteolytic cleavage (Martin D. D. O et al., 2011). We previously identified the post-translational myristoylation of a short fragment of huntingtin (HTT_553-586_) at glycine 553 (G553), following caspase-3 cleavage of HTT_1-588_-YFP at aspartate 552 (D552) (Martin et al., 2012; Martin et al., 2014). Overexpressed myristoylated HTT_553-586_ fragment robustly induced autophagy, which is defective in HD (Martinez-Vicente et al., 2010; Ochaba et al., 2014). In addition, while studying a novel caspase-cleavage site in HTT_1-588_, we noted that blocking proteolysis of wild-type and mutant huntingtin at the pathogenic site D586 promoted the generation of post-translationally myristoylated HTT (Martin et al., 2018a). In turn, we recently found that the protective effect of preventing mutant HTT proteolysis at amino acid 586 by caspases 6 and 8 in the YAC128 mouse model (C6R mouse line) makes mutant HTT a better substrate for autophagy while also promoting global autophagy (Ehrnhoefer et al., 2018). Conversely, transient expression of wild-type HTT_1-588_ carrying a human mutation that blocks myristoylation (G553E) was toxic and induced apoptosis in HD striatal-like cells (ST*Hdh* ^111^) suggesting that post-translational myristoylation is protective in HD (Martin et al., 2018b). Finally, HTT proteolysis at multiple sites is toxic, while proteolysis at one site is not, which promotes the interaction of N- and C-terminal HTT fragments (El-Daher et al., 2015). This led us to propose a PTM crosstalk model in which impairing D586 cleavage promotes D552 cleavage and post-translational myristoylation of HTT at G553 (Graham et al., 2006; Wong et al., 2015; Ehrnhoefer et al., 2018).

The study of HTT PTMs has mostly been restricted to the N-terminal region of HTT (1–600 amino acid region), and little is known about the modifications located in the remaining protein (600–3,144 amino acids). With advances in the detection of palmitoylation, it has become apparent that there are likely additional palmitoylation sites within HTT, as the mutation of the C214 residue (C214S) reduces but does not abrogate HTT palmitoylation. Here, we investigate and identify the existence of additional palmitoylation sites within the full-length HTT protein and further characterize post-translational myristoylation in the context of full-length HTT.

## 2 Materials and methods

### 2.1 Materials

#### 2.1.1 Reagents and chemicals

Table 1 lists the reagents and chemicals used for this study along with the application, manufacturer and catalogue number or bibliographic reference.

**TABLE 1:** List of reagents and chemicals used.

	Reagent	Manufacturer	#Catalogue or PMID
**Cloning**	Agarose	Invitrogen	#16500
	gBlock gene fragment	Integrated DNA tech	–
	MAX Efficiency Stbl2 Competent Cells	Invitrogen	#10268019
	mCherry-23/100Q-HTT_1-3144_-EGFP WT, TEV552, TEV586	–	PMID: 26165689
	pCI-neo 15Q-HTT_1-548_	In-house	PMID: 15603740
	pCI-neo 15Q-HTT_1-1212_	In-house	PMID: 18992820
	15Q-HTT_1-3144_	In-house	PMID: 10770929
	QIAquick Gel Extraction Kit	Qiagen	#28706
	Quick Ligation Kit	New England Biolabs	#M2200
	Restriction Enzymes (EcoRI, PspXI, PshAI, EcoRV, PciI, XbaI)	New England Biolabs	-
	Subcloning Efficiency DH5α Competent Cells	Invitrogen	#18265017
**Cell Culture Reagents**	Dimethyl sulfoxide (DMSO)	Millipore-Sigma	#D2650
	Fetal Bovine Serum (FBS), qualified, Canada	Gibco	#12483020
	GlutaMAX Supplement	Gibco	#35050061
	Nunc EasYFlask Cell Culture 75 cm^2^	Thermo Fisher Scientific	#156472
	Penicillin-Streptomycin (10,000 U/mL)	Gibco	#15140122
	Pepstatin A Protease Inhibitor	Thermo Fisher Scientific	#78436
	Phosphate-Buffered Saline (PBS) (10X)	Gibco	#70011044
	Phenylmethylsulfonyl fluoride (PMSF) protease inhibitor	Thermo Fisher Scientific	#36978
	Sodium pyruvate (100 mM)	Gibco	#11360070
	Trypsin-EDTA (0.25%), phenol red	Gibco	#25200056
	X-tremeGENE 9 DNA Reagent	Roche	#6365809001
**Fatty Acylation Assays**	Biotin Azide	Invitrogen	#B10184
	Caspase inhibitor I (Z-VAD (OMe)-FMK)	Calbiochem	#627610
	Charcoal-stripped FBS	Gibco	#12676029
	Click Tag Myristic Acid Alkyne (13-tetradecynoic acid)	Cayman Chemical	#13267
	Click Tag Palmitic Acid Alkyne	Cayman Chemical	#13266
	Cycloheximide (CHX)	Millipore-Sigma	#C7698
	Dulbecco’s Modified Eagle (DMEM)	Gibco	#11960044
	Dynabeads Protein G	Invitrogen	#10003D
	EZ-Link BMCC-Biotin	Thermo Fisher Scientific	#21900
	Fatty acid free Bovine Serum Albumin (BSA)	Millipore-Sigma	#A6003
	Hydroxylamine hydrochloride (HA)	Millipore-Sigma	#255580
	N-ethylmaleimide (NEM)	Sigma-Aldrich	#E3876
	Re-blot Plus Strong Solution (10X)	Millipore-Sigma	#2504
	Sodium Dodecyl Sulfate (SDS)	Thermo Fisher Scientific	#BP166-500
	Staurosporine (STS) solution	Millipore-Sigma	#S6942
	Triton X-100	Roche	#T9284
	Tris (benzyltriazolylmethyl)amine (TBTA)	Millipore-Sigma	#678937
	Tris-carboxyethylphosphine (TCEP)	Millipore-Sigma	#C4706
**Western Blot Analysis**	Amersham Hybond P WB membranes, PVDF	Cytiva	#10600023
	Amersham Protran WB membranes, nitrocellulose	Cytiva	#GE10600020
	Complete Protease Inhibitor Cocktail	Roche	#11836145001
	Complete EDTA-free protease inhibitors	Roche	#04693132001
	Dithiothreitol (DTT)	Millipore-Sigma	#3483–12–3
	Gel Cassettes, mini, 1.5 mm	Thermo Fisher Scientific	#NC2015
	Invitrogen UltraPure TEMED	Thermo Fisher Scientific	#15524010
	Methanol	Thermo Fisher Scientific	#A412P4
	NuPAGE LDS Sample Buffer (4X)	Thermo Fisher Scientific	#NP0007
	NuPAGE Transfer Buffer (20X)	Invitrogen	#NP00061
	NuPAGE Novex 3%–8% Tris-Acetate gels	Thermo Fisher Scientific	#EA0375
	PROTEAN II xi Cell system	Bio-Rad	#1651804
	Protein Assay Reagent A	Bio-Rad	#5000113
	Protein Assay Reagent B	Bio-Rad	#5000114
	Tween 20	Thermo Fisher Scientific	#BP337-500
	2% Bis Solution	Bio-Rad	#1610142
	30% Acrylamide/Bis Solution, 29:1	Bio-Rad	#1610156
	40% Acrylamide Solution	Bio-Rad	#1610140

#### 2.1.2 Antibodies

Table 2 displays the list of antibodies used for this study along with the application, concentrations and experimental conditions.

**TABLE 2:** List of antibodies used and application.

Target (Clone)	Species	Type	Supplier	Catalogue	Application	Dilution	Volume^a^
				AB registry reference			
HTT	Mouse	Monoclonal	Sigma-Aldrich	MAB2166	WB	1:1,000	–
(1HU-4C8)				AB_2123255	IP	–	2 µL
HTT	Rabbit	Monoclonal	Cell Signaling Technology	5656	WB	1:1,000	–
(D7F7)				AB_10827977			
HTT	Rabbit	Polyclonal	in-house	Kalchman et al. (1996)	IP	–	2 µL
(BKP1/HD46)					WB	1:400	–
HTT	Rabbit	Polyclonal	Sigma-Aldrich	H7540	WB	1:1,000	–
				AB_1840946	IP	–	2 µL
mCherry	Rabbit	Polyclonal	Abcam	ab167453	WB	1:1,000	–
Streptavidin	–	Conjugated to AF-680	Invitrogen	S-32358	WB	1:5,000	–
GFP	Goat	Polyclonal	Eusera	EU4	IP	–	0.75 µL
GFP	Rabbit	Polyclonal	Eusera	EU2	WB	1:5,000	–
					IP	–	0.75 µL
Goat IgG	–	–	Thermo Fisher Scientific	02–6202	IP	–	0.75 µL
Mouse IgM (µ chain specific)	Goat	–	Rockland Immuno-chemicals	92,632,210	WB (secondary)	1:5,000	–
Rabbit IgG	Goat	–		926–32211	WB (secondary)	1:5,000	–

^a^ Volume of antibodies used for 20 µL of Dynabeads and 200 μg–2 mg proteins.

#### 2.1.3 Plasmids

**FIGURE 1:**
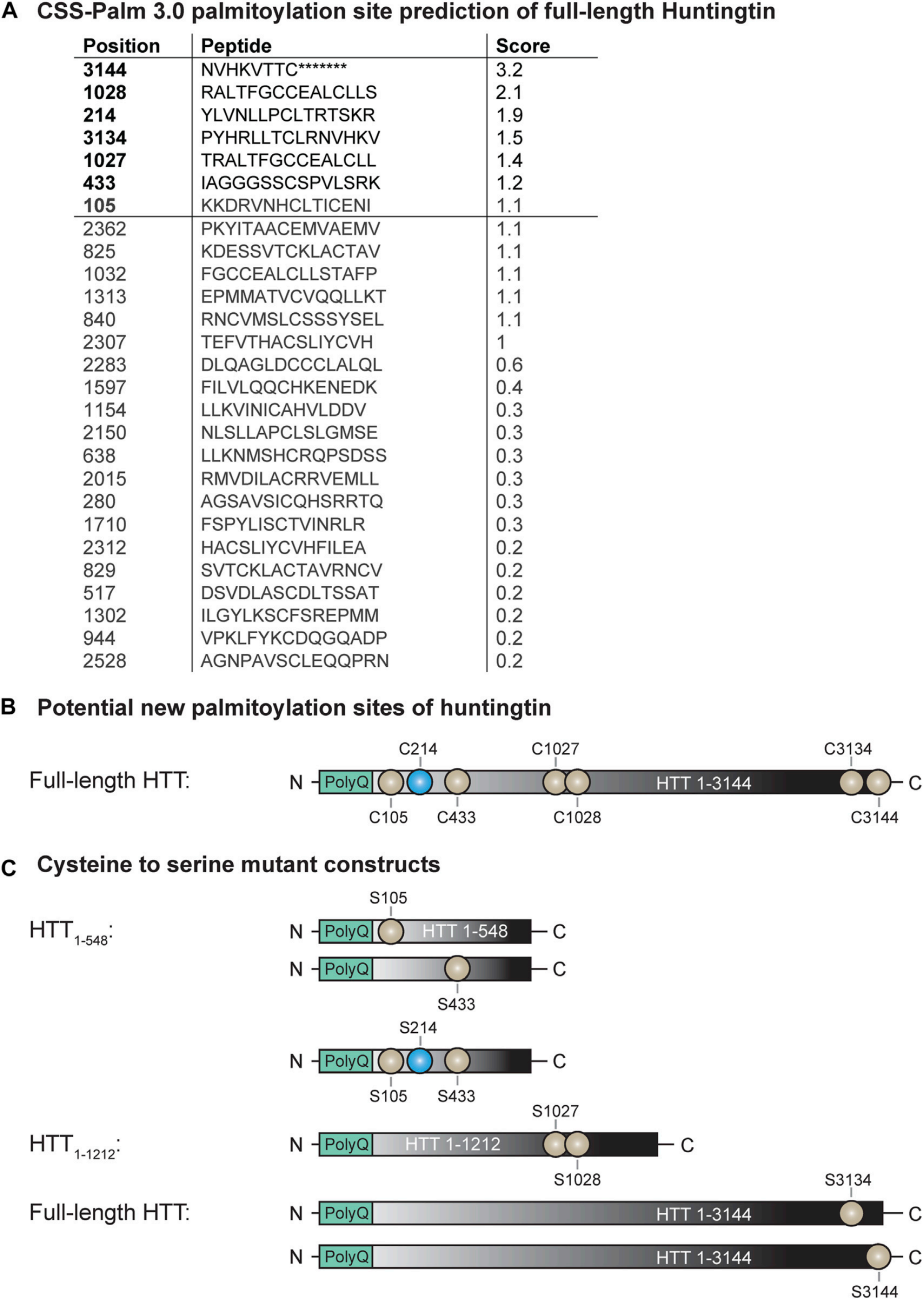
Potential new palmitoylation sites of huntingtin. **(A)** CSS-Palm 3.0 palmitoylation site prediction of full-length HTT, **(B)** palmitoylation site of HTT (in blue: C214) and potential new palmitoylation sites investigated in this study (in light grey: C105, C433, C1027, C1028, C3134 and C3144), **(C)** cysteine-to-serine mutant DNA constructs generated to investigate the potential new palmitoylation sites of huntingtin. Point mutations were introduced in N-terminal HTT_1-548_, HTT_1-1212_ or full-length HTT_1-3144_ at C105, C214, C433, C105/214/433, C1027/C1028, C3134 and C3144.

15Q-HTT_1-548_(C105S): gBlock^®^ gene fragment coding for 15Q-HTT carrying the C105S mutation and including EcoRI and PspXI restriction sites were designed and ordered from IDT. The gBlock^®^ gene fragment and the pCI-neo 15Q-HTT_1-548_ construct [15Q 1955; NM_002111; (Wellington et al., 1998)] were digested with EcoRI and PspXI restriction enzymes, gel-purified using the QIAquick gel extraction kit and ligated using Quick Ligation Kit followed by transformation of MAX Efficiency Stbl2 competent cells (Figure 1C). 15Q-HTT_1-548_(C433S): gBlock^®^ gene fragment coding for carrying the C433S mutation and the flanking PshAI and EcoRV restriction sites were designed and ordered from IDT. The gBlock^®^ gene fragment and the pCI-neo 15Q-HTT_1-548_ construct were digested with PshAI and EcoRV restriction enzymes, gel-purified and ligated (Figure 1C). 15Q-HTT_1-548_(C105/214/433S): The 15Q-HTT_1-548_(C214S), (C105S) and (C433S) plasmids were digested with PspXI, PshAI, EcoRV and EcoRI restriction enzymes to isolate on agarose gel and purify fragments and open vector containing each CS mutations that were then ligated (Figure 1C). 15Q-HTT_1-1212_(C1027/1028S): gBlock^®^ gene fragment coding for an HTT fragment carrying the C1027S and C1028S mutations and including PciI and XbaI restriction sites were designed and ordered from IDT. The gBlock^®^ fragment and the pCI-neo 15Q-HTT_1-1212_ construct [15Q 3949; NM_002111; (Warby et al., 2009)] were digested with PciI and XbaI restriction enzymes, purified and ligated, followed by transformation of MAX Efficiency Stbl2 competent cells (Figure 1C). 15Q-HTT_1-3144_(C3134S) and (C3144S): The plasmids were generated using PCR based site directed mutagenesis using primers carrying the C3134S or C3144S mutations followed by ligation at the SalI sites in full-length HTT (15Q-HTT) (Wellington et al., 2000) (Figure 1C). C-term HTT_553-3144_-EGFP WT, G553A and mCherry-23Q and 100Q-HTT_1-3144_-EGFP WT, G553A, TEV586 and TEV552: Dr. Saudou kindly shared the mCherry-23Q and 100Q-HTT_1-3144_-EGFP with and without caspase cleavage sites at D552 and D586 replaced with Tobacco Etch virus (TEV) proteolytic sites (El-Daher et al., 2015). The G553A myristoylation-resistant mutation was introduced in the mCherry-23Q and 100Q-HTT_1-3144_-EGFP WT by TOP Gene Technologies (Quebec, Canada). The HTT_553-3144_-EGFP WT and G553A constructs were generated by TOP Gene Technologies, by excising the mCherry-23Q and 100Q-HTT_1-552_ fragments from the mCherry-23Q and 100Q-HTT_1-3144_-EGFP WT and G553A plasmids, and by introducing a N-terminal methionine (Figures 4A, C; Figure 5A). All the constructs were verified by DNA sequencing (CMMT facility).

#### 2.1.4 Experimental models (cell lines)

HeLa, HEK293 and COS-7 cells were grown at 37°C using in Dulbecco’s Modified Eagle Medium (DMEM) supplemented with 10% fetal bovine serum (FBS), 1 mM sodium pyruvate, 2 mM GlutaMAX and 10 units/mL of penicillin and 10 μg/mL of streptomycin. Cells were grown in 75 cm^2^ flasks at 37°C and 5% CO_2_, and were split using a 0.25% trypsin-EDTA solution (1:10 dilution) when cells were ∼90% confluent by microscopy.

### 2.2 Methods

#### 2.2.1 Plasmid transfection

Cells were seeded in 6-well or 10-cm plates overnight at 37°C and 5% CO_2_. The next day, cells were transfected with cDNA constructs encoding for 15Q-HTT_1-548_, HTT_1-1212_ and full-length HTT_1-3144_, mCherry-23Q or 100Q-HTT_1-3144_-EGFP and HTT_553-3144_-EGFP using X-tremeGENE 9 DNA transfection reagent (ratio 3:1 or 6:1, reagent (μL): DNA (μg)) or calcium phosphate transfection (Graham and van der Eb, 1973). Cells were treated or harvested 18–48 h post-transfection.

#### 2.2.2 Palmitoylation assays

##### 2.2.2.1 Bio-orthogonal labeling assay using alkyne-palmitate (click chemistry)

To measure the dynamic palmitoylation level of HTT, cells transiently transfected with HTT constructs for 18 h were incubated for 1 h in DMEM supplemented with 5% charcoal-stripped FBS, to deprive the cells of lipids. Cells were subsequently metabolically labeled with 100 µM alkyne-palmitate in fatty acid-free bovine serum albumin (BSA) for 6 h. Cells were lifted from plates in phosphate-buffered saline (PBS) using cell scrapers, pelleted by centrifugation (500 × *g*, 5 min, +4°C) and subsequently lysed in EDTA-free RIPA buffer (150 mM NaCl_2_, 50 mM HEPES pH 7.4, 1% Igepal CA-630, 0.5% sodium deoxycholate, 0.1% sodium dodecyl sulfate (SDS) in water, EDTA-free cOmplete protease inhibitors) for 10 min on ice, 5 min at 4°C on rotator and were centrifuged (16,000 × *g*, 10 min, +4°C). Protein concentrations were assessed using the DC protein assay. HTT was immunoprecipitated from cell lysates by overnight incubation with Dynabeads Protein G and appropriate antibodies (Table 2). Bio-orthogonal click chemistry of alkyne-palmitate with biotin azide was performed on cell lysates, as previously described (Yap et al., 2010; Liao et al., 2021). The immunoprecipitated proteins were adjusted to 1% SDS and incubated with 100 mM tris (benzyltriazolylmethyl)amine (TBTA), 1 mM CuSO_4_, 1 mM tris-carboxyethylphosphine (TCEP) and 100 mM biotin azide (PEG4 carboxamide-6-Azidohexanyl biotin) at 37°C in darkness for 30 min. Total HTT and palmitoylated HTT were detected by western blot analysis.

##### 2.2.2.2 Acyl-biotin exchange assay on immunoprecipitated proteins (IP-ABE)

The IP-ABE assay was performed as previously described to measure the global palmitoylation level of HTT (Drisdel and Green, 2004; Huang et al., 2009). Briefly, cells transiently expressing HTT constructs for 48 h were lifted from plates in PBS using cell scrapers and then pelleted by centrifugation (500 × *g*, 5 min, +4°C). Subsequently, cell pellets were lysed on ice in lysis buffer (150 mM NaCl, 50 mM tris, 5 mM EDTA, 0.1% SDS, 1% Triton X-100, pH 7.4) containing 100 mM N-ethylmaleimide (NEM). Homogenates were sonicated to shear DNA, and the insoluble material was removed by centrifugation (20,000 × *g*, 15 min, +4°C). Protein concentrations in lysates were assessed by DC protein assay. HTT was immunoprecipitated from cell lysates by overnight incubation with Dynabeads Protein G and appropriate antibodies (Table 2). Beads were then washed and split into two and treated with neutral pH hydroxylamine in lysis buffer (HAM+) or just lysis buffer (HAM-) for 2 h. Following HAM treatment, beads were washed and treated with 2.5 µM EZ-Link BMCC-Biotin in pH 6.2 lysis buffer for 1 h at 4°C. At the end of the BMCC-Biotin treatment, beads were washed and then heated for 10 min at 70°C with NuPAGE LDS sample buffer and 100 mM dithiothreitol (DTT) to elute proteins. Total HTT and palmitoylated HTT were detected by western blot analysis.

#### 2.2.3 Myristoylation assay (bio-orthogonal labeling assay using alkyne-myristate)

Myristoylation of HTT was detected as previously described (Yap et al., 2010; Martin et al., 2012; Martin et al., 2014; Martin et al., 2018b; Liao et al., 2021). HEK293 cells were transiently transfected with the HTT_553-3144_-EGFP WT and G553A plasmids using the calcium phosphate transfection protocol. The following day, the cells were starved of lipids in DMEM supplemented with 5% charcoal-stripped FBS for 30 min, and then treated with 100 µM alkyne-myristate in fatty acid-free BSA for 4 h. HeLa cells were transiently transfected with mCherry-23Q and 100Q-HTT_1-3144_-EGFP WT, G553A, TEV552 and TEV586 plasmids using X-tremeGENE 9 as per the manufacturer’s directions. HeLa cells transfected for 40 h were washed in PBS and starved in DMEM supplemented with 5% charcoal-stripped FBS for 30 min. HeLa cells were then incubated with 100 µM alkyne-myristate in fatty acid-free BSA for 30 min, prior to the addition of 1 µM staurosporine (STS) to promote caspase activity, and cycloheximide (CHX) at 5 μg/mL to inhibit protein synthesis for 4 h. HEK293 and HeLa cells were lysed in modified EDTA-free RIPA buffer (50 mM HEPES pH 7.4, 0.5% sodium deoxycholate, 150 mM NaCl, 1% Igepal, 0.1% SDS, 2 mM MgCl_2_) supplemented with PMSF, pepstatin A and caspase inhibitor I (Z-VAD-FMK). Proteins were immunoprecipitated from cell lysates by overnight incubation with Protein G Dynabeads and goat anti-GFP antibodies, and subsequently subjected to click chemistry, as previously described in the section ‘Bio-orthogonal labeling assay using alkyne-palmitate’. Sample denaturation was conducted in NuPAGE LDS sample buffer and 100 mM DTT for 10 min at 70°C. Total HTT and myristoylated HTT were detected by western blot analysis.

#### 2.2.4 Co-immunoprecipitation (Co-IP)

HeLa cells were seeded in 6-well plates and transfected with the mCherry-23Q-HTT_1-3144_-EGFP WT, TEV552, TEV586 or G553A using X-tremeGENE 9 Transfection reagent for 46 h, and treated for 2 h with 5 µM STS and 5 μg/mL cycloheximide (STS/CHX). Cells were then lysed in SDP lysis buffer (50 mM tris pH 8.0, 150 mM NaCl, 1% Igepal, 10 mM NaF, 40 mM β-glycerophosphate) supplemented with fresh protease inhibitors (cOmplete protease inhibitor cocktail, 1 mM sodium orthovanadate, 1 µM pepstatin A, and 5 µM Z-VAD-FMK), homogenized using an Eppendorf homogenizer, rotated at 4°C for 20 min and centrifuged (16,000 × *g*, 12 min, +4°C). Protein concentrations in the supernatants were assessed by DC assay. Lysates were pre-cleared with 10 µL Dynabeads Protein G for 30 min at 4°C. Full-length and C-terminal HTT-EGFP were immunoprecipitated from lysates by overnight incubation with Dynabeads Protein G and goat anti-GFP antibodies. Proteins were eluted from beads and denatured in NuPAGE LDS sample buffer with 100 mM DTT for 15 min at 70°C. Immunoblots for N-terminal HTT fragments were conducted with HTT [MAB2166/1HU-4C8, amino acid ∼443–457 (Cong et al., 2005)], or mCherry antibodies. Immunoblots for full-length and C-terminal HTT fragments were conducted with rabbit anti-GFP antibodies.

#### 2.2.5 Western blot analysis

For the palmitoylation assays, denatured protein samples were run on NuPAGE 3%–8% tris-acetate gradient protein gels in tris-acetate SDS running buffer (50 mM tricine, 50 mM tris base, 0.1% SDS, pH 8.24). Proteins were transferred to 0.45 μm nitrocellulose membranes, in NuPAGE transfer buffer supplemented with 5% methanol. For the myristoylation assay, the lysates from HEK293 cells expressing C-terminal HTT_553-3144_-EGFP were run on tris-glycine gels (7%) in SDS-tris-glycine running buffer (25 mM tris base, 190 mM glycine, 3.5 mM SDS). Lysates from HeLa cells expressing mCherry-HTT-EGFP were run on 10% low bis acrylamide (1:200 bis-acrylamide:acrylamide) gels (without β-mercaptoethanol) in SDS-tris-glycine running buffer (Carroll et al., 2011; Caron et al., 2021). Proteins were transferred to 0.45 μm PVDF membranes, in NuPAGE transfer buffer supplemented with 5% methanol. For the co-immunoprecipitation assay, denatured HeLa lysates were run on large tris-glycine gels (10%) in SDS-tris-glycine running buffer using the Bio-Rad PROTEAN II xi Cell system. Proteins were transferred to 0.45 μm nitrocellulose membranes, in NuPAGE transfer buffer supplemented with 5% methanol.

PVDF and nitrocellulose membranes were blocked in 3%–5% BSA in tris-buffered saline (TBS) supplemented with 0.1%–0.5% Tween-20 (T). Primary antibody dilutions in 3%–5% BSA TBST were applied to the immunoblots at room temperature for 1-2 h or overnight at 4°C (Table 2). Membranes were washed in PBST or TBST (4 × 5 min, room temperature). The appropriate secondary antibodies and Alexa Fluor 680 conjugated streptavidin were then applied in 3%–5% BSA TBST for 1-2 h at room temperature. Membranes were washed in TBST (4 × 5 min, room temperature) and imaged using the LI-COR Odyssey Infrared Imaging System (LI-COR Biosciences). To perform membrane stripping, PVDF membranes were washed with PBS, submerged in Re-blot plus strong solution and incubated at 55°C for 30 min with intermittent agitation. The membranes were washed twice in TBST (2 × 10 min, room temperature), blocked for 1 h in 3% BSA in TBST, and re-probed with primary and secondary antibodies. Densitometry was quantified using the LI-COR Image Studio Lite software and median signal intensity following background subtraction was used for analysis. Acylation was analyzed as a ratio of myristoylation or palmitoylation signal (streptavidin) to total immunoprecipitated protein signal and normalized to the control. All the uncropped immunoblots are displayed in Supplementary Figures S1–S10.

#### 2.2.6 Statistical analysis

GraphPad Prism 9 (9.4.1) was used for all statistical analyses and graph preparation. Figures were generated in Adobe Illustrator 2020. All data are presented as mean ± SEM. Biological replicates (n) are displayed on each graph, or indicated in the captions. Student’s *t*-test or 1, 2, or 3-way ANOVA statistical tests with post-hoc analysis (Sidak, Tukey, Bonferroni) were used for all experiments.

## 3 Results

### 3.1 Identification of new palmitoylation sites of huntingtin within its N-terminal region

**FIGURE 2:**
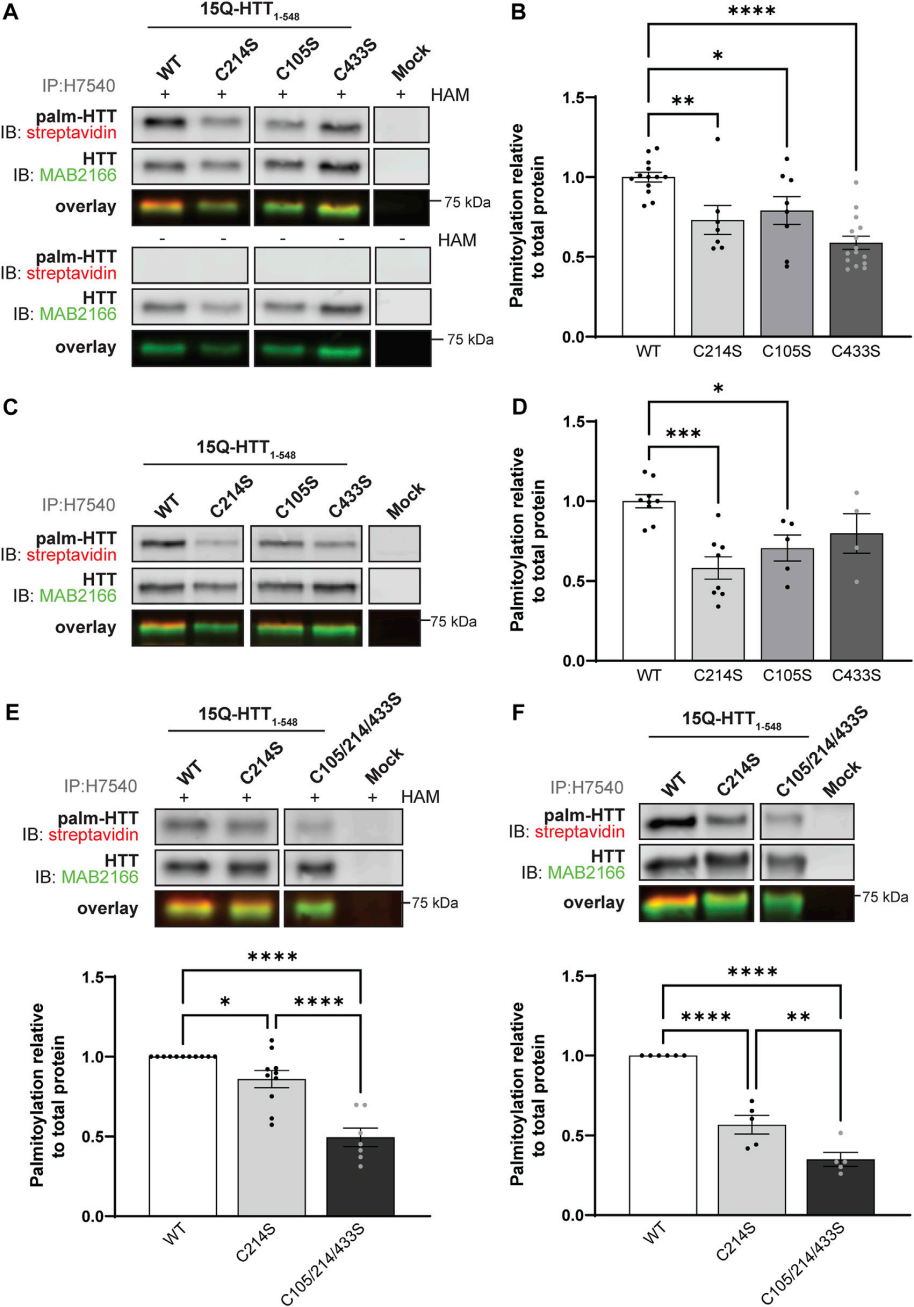
Huntingtin is palmitoylated at cysteines 105 and 433. **(A)** COS-7 cells transiently expressing 15Q-HTT_1-548_ WT or carrying cysteine-to-serine mutations at residues 214 (C214S), 105 (C105S) or 433 (C433S) were harvested 48 h post-transfection, and subjected to the IP-ABE assay. Mock transfections were included to determine baseline background levels. Proteins were immunoprecipitated from lysates using N-terminal HTT antibodies (H7540). Palmitoylated HTT labeled with biotin was detected by western blot analysis using streptavidin, and total HTT with MAB2166 antibodies. The palmitoylation signal (palm-HTT, HAM+) and the negative control treatment (palm-HTT, HAM-) are shown in the top panels, the total HTT protein immunoprecipitated are presented in the corresponding middle panels, and the overlay of the two are displayed in the bottom panels. The western blot images are composites of different lanes from the same image (Supplementary Figure S1), **(B)** HTT palmitoylation level was calculated as the ratio of palmitoylation over total HTT protein signal, and expressed relative to 15Q-HTT_1-548_ WT (n = 7–15), **(C)** COS-7 cells transiently expressing 15Q-HTT_1-548_ WT or C214S, C105S and C443S were labeled with alkyne-palmitate for 6 h, and harvested 48 h post-transfection. Proteins were immunoprecipitated from lysates with H7540 antibodies, and bio-orthogonally labeled with azido-biotin by click chemistry. Palmitoylated HTT_1-548_ (top panel) labeled with biotin was detected with streptavidin, and total HTT (corresponding middle panel) was detected with HTT antibodies (MAB2166). The overlay of the two is displayed in the bottom panel. The western blot images are composites of different lanes from the same image (Supplementary Figure S2) **(D)** HTT palmitoylation level was calculated as the ratio of palmitoylated HTT over total HTT protein signal, and expressed relative to 15Q-HTT_1-548_ WT (n = 5–7) **(E)** Palmitoylation of 15Q-HTT_1-548_ WT, C214S, C105/214/433S expressed in COS-7 cells was measured as described in **(A, B)**, by IP-ABE assay (n = 7–11) **(F)** Palmitoylation of 15Q-HTT_1-548_ WT, C214S, C105/214/433S expressed in COS-7 cells was measured as described in **(C, D)**, by bio-orthogonal labeling following metabolic labeling with alkyne-palmitate (n = 5–6). The western blot images are composites of different lanes from the same image (Supplementary Figures S3, S4). *Statistical analysis*: **(B)** 1-way ANOVA: C to S mutations, ****p < .0001. Tukey’s multiple comparisons test: WT vs. C214S, **p = .0084; WT vs. C105S, *p = .040; WT vs. C433S, ****p < .0001. **(D)** 1-way ANOVA: C to S mutations, ***p = .0009. Tukey’s multiple comparisons test: WT vs. C214S, ***p = .0003; WT vs. C105S, *p = .024; WT vs. C433S, *p* = .23. **(E)** 1-way ANOVA: C to S mutations, ****p < .0001. Tukey’s multiple comparisons test: WT vs. C214S, *p = .044; WT vs. C105/214/433S, ****p < .0001; C214S vs. C105/214/433S, ****p < .0001. (**F**) 1-way ANOVA: C to S mutations, ****p < .0001. Tukey’s multiple comparisons test: WT vs. C214S, ****p < .0001; WT vs. C105/214/433S, ****p < .0001; C214S vs. C105/214/433S, **p = .0058.

**FIGURE 3:**
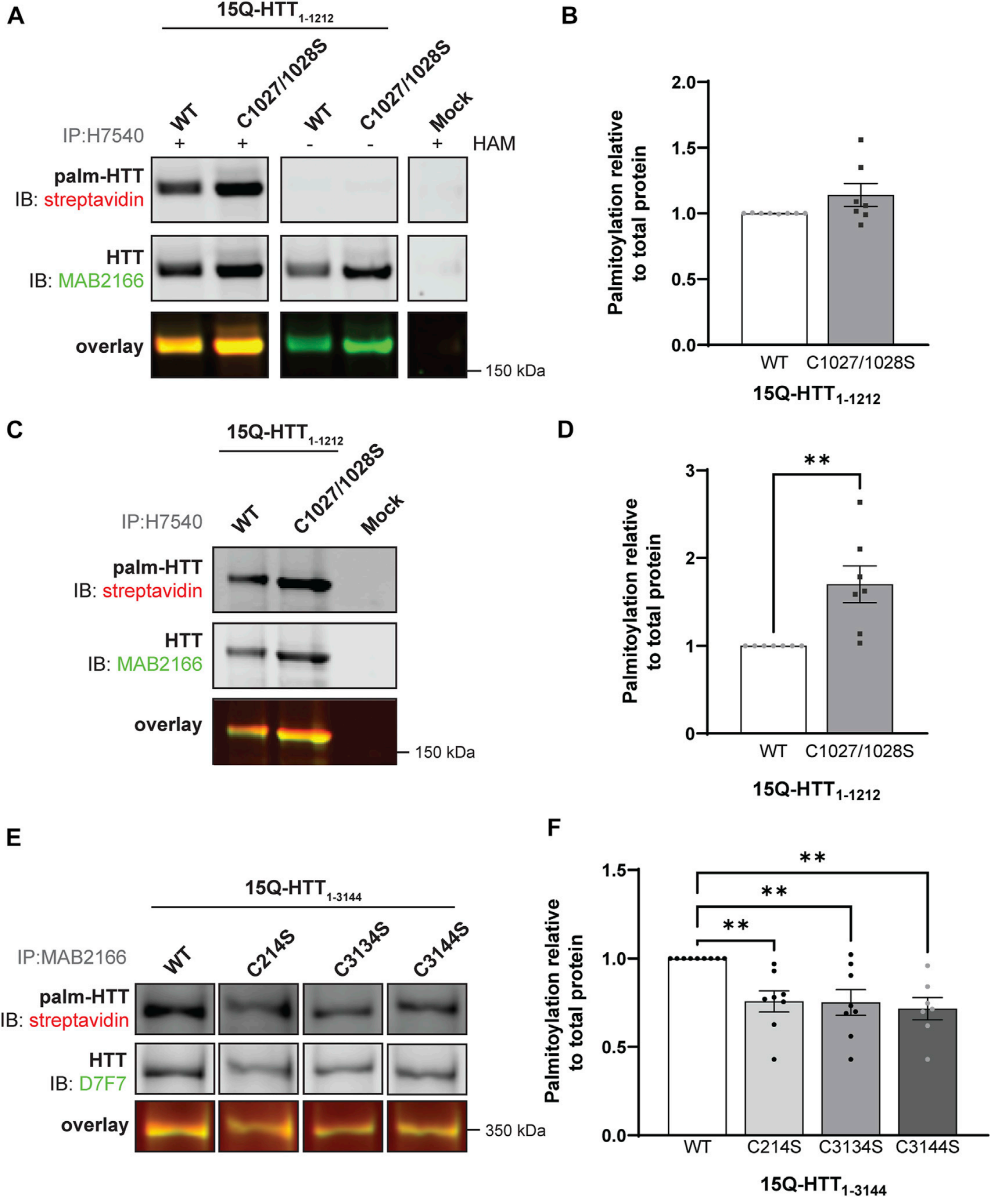
Huntingtin is palmitoylated at cysteines 3134 and 3144. **(A)** COS-7 cells transiently expressing 15Q-HTT_1-1212_ WT or carrying two cysteine-to-serine mutations at residues 1027 and 1028 (C1027/1028S) were harvested 48 h post-transfection, and subjected to the IP-ABE assay. Proteins were immunoprecipitated from lysates using N-terminal HTT antibodies (H7540). Palmitoylated HTT labeled with biotin was detected by western blot analysis using streptavidin, and total HTT with MAB2166 antibodies. The palmitoylation signal (palm-HTT, HAM+) and the negative control treatment (HAM-) are shown in the top panels, the total HTT protein immunoprecipitated is presented in the corresponding middle panels, and the overlay of the two is displayed in the bottom panels. Uncropped blots are displayed in Supplementary Figure S5, **(B)** HTT palmitoylation level was calculated as the ratio of palmitoylated HTT over total HTT protein signal, and represented relative to 15Q-HTT_1-1212_ WT (n = 7), **(C)** COS-7 cells transiently expressing 15Q-HTT_1-1212_ WT or C1027/1028S were labeled with alkyne-palmitate for 6 h, and harvested 48 h post-transfection. Proteins were immunoprecipitated from lysates with H7540 antibodies, and bio-orthogonally labeled with azido-biotin by click chemistry. Palmitoylated HTT_1-1212_ (top panel) labeled with biotin was detected with streptavidin, and total HTT (corresponding middle panels) was detected with HTT antibodies (MAB2166). The overlay of the two is displayed in the bottom panels. Uncropped blots are displayed in Supplementary Figure S5, **(D)** HTT palmitoylation level was calculated as the ratio of palmitoylated HTT over total HTT protein signal, and represented relative to 15Q-HTT_1-1212_ WT (n = 7), **(E)** HeLa cells transiently expressing 15Q-HTT_1-3144_ WT, C214S, C3134S and C3144S were labeled with alkyne-palmitate for 6 h, and harvested 48 h post-transfection. Proteins were immunoprecipitated from lysates with HTT (MAB2166) antibodies, and bio-orthogonally labeled with azido-biotin by click chemistry. Palmitoylated HTT_1-3144_ (top panel) labeled with biotin was detected with streptavidin, and total HTT (corresponding middle panels) was detected with HTT antibodies (D7F7). The overlay of the two is displayed in the bottom panels. The western blot images are composites of different lanes from the same image (Supplementary Figure S6), **(F)** HTT palmitoylation level was calculated as the ratio of palmitoylated HTT over total HTT protein signal, and represented relative to 15Q-HTT_1-3144_ WT (n = 7–8). *Statistical analysis*: **(B)** Unpaired *t*-test: *p* = .11. **(C)** Unpaired *t*-test: **p = .0058. **(F)** 1-way ANOVA: C to S mutations, **p = .0024. Bonferroni’s multiple comparisons test: WT vs. C214S, **p = .0090; WT vs. C3134S, **p = .0073; WT vs. C3144S, **p = .0031.

We have previously shown that HTT is palmitoylated at cysteine 214 (C214), using radioactive labeling techniques (Yanai et al., 2006). With recent advances in the detection of palmitoylation it has become apparent that there are likely additional palmitoylation sites within HTT, as the C214S mutation only reduces HTT palmitoylation levels by 30%–40% (*unpublished data*; displayed in Figures 2, 3). To identify these potential palmitoylation sites, the HTT amino acid sequence was run through the CSS-Palm 3.0 prediction program to determine which cysteines are predicted to be palmitoylated (Figure 1A) (Zhou et al., 2006). Cysteine residues with a prediction score higher than 1.2 were prioritized for testing as potential palmitoylation sites (3144, 1028, 3134, 1027 and 433). We also included the cysteine 105 as it is localized in the N-terminal 548 amino acid fragment, which contains the interaction domain of HTT with ZDHHC17 and 13 (Sanders et al., 2014) (Figure 1B).

In order to reduce the number of potential sites detected, we first sought to identify new palmitoylated residues within the N-terminal 548 amino acid fragment (Figure 1C and Figure 2; uncropped blots displayed in Supplementary Figures S1–S4). The palmitoylation level of 15Q-HTT_1-548_ WT, carrying a cysteine-to-serine mutation at C214 (C214S), C105 (C105S) or C433 (C433S) was measured in COS-7 cells using the IP-ABE assay (Figure 2A). The palmitoylation level of HTT C214S was significantly decreased compared to the WT control (by 30%; 1-way ANOVA: *p* < .0001; Tukey’s test: WT vs. C214S, *p* = .0084), but not abrogated (Figure 2B). Palmitoylation levels of 15Q-HTT carrying the C105S or C433S mutations were significantly lower than the WT control, by 20% and 40%, respectively (Tukey’s test: WT vs. C105S, *p* = .040; WT vs. C433S, *p* < .0001). This result supports that C214 is not the only palmitoylated residue of HTT, and that C105 and C433 are two palmitoylation sites within the huntingtin protein.

The dynamic palmitoylation level of 15Q-HTT_1-548_ WT, C214S, C105S and C433S was also measured in COS-7 cells by bio-orthogonal labeling following metabolic labeling with alkyne-palmitate (Figure 2C). As expected, the HTT C214S palmitoylation level was significantly reduced compared to the WT control (by 40%; 1-way ANOVA: *p* = .0009; Tukey’s test: WT vs. C214S, *p* = .0003) (Figure 2D). The palmitoylation level of 15Q-HTT C105S was significantly decreased compared to the WT control (by 30%; Tukey’s test: WT vs. C105S, *p* = .024). The data support that the residue C105 of the HTT protein is dynamically palmitoylated, similar to C214. While the palmitoylation level of 15Q-HTT C433S was lower than that of the WT control (20%), this decrease did not reach statistical significance due to a higher variability of the data (Tukey’s test: WT vs. C433S, *p* = .23). This result could be indicative of a slower palmitoylation turnover of HTT at C433.

The palmitoylation levels of 15Q-HTT_1-548_ WT, C214S or carrying three cysteine-to-serine mutations at C105, 214 and 433 (C105/214/433S) were measured in COS-7 cells using the IP-ABE (Figure 2E) and the bio-orthogonal assays (Figure 2F). The palmitoylation level of 15Q-HTT C105/214/433S was significantly reduced compared to 15Q-HTT WT (by 50%; Tukey’s test: *p* < .0001) and 15Q-HTT C214S (by 40%; Tukey’s test: *p* < .0001) when measured with the IP-ABE assay (Figure 2E). The dynamic palmitoylation level of 15Q-HTT C105/214/433S was also significantly decreased compared to 15Q-HTT WT (by 65%; Tukey’s test: *p* < .0001) and 15Q-HTT C214S (by 40%; Tukey’s test: *p* < .0001) when measured by bio-orthogonal labeling assay (Figure 2F). Our data show an additive effect of the cysteine residues C214, C105 and C433 on the global and dynamic palmitoylation levels of HTT. Altogether, these results support that the cysteines 105 and 433 of HTT are palmitoylated.

### 3.2 Identification of new palmitoylation sites of huntingtin within its C-terminal region

Next, we investigated the existence of potential new palmitoylated residues within HTT at C1027 and C1028 by using a longer fragment of HTT, 15Q-HTT_1-1212_ (Figure 1C and Figure 3 **;** uncropped blots displayed in Supplementary Figure S5). The global palmitoylation level of 15Q-HTT_1-1212_ WT, or carrying two cysteine-to-serine mutations at C1027 and C1028 (C1027/1028S) was measured in COS-7 cells using the IP-ABE assay (Figure 3A). The palmitoylation level of 15Q-HTT C1027/1028S was not significantly modified compared to the WT control (Figure 3B; *t*-test: *p* = .11). The dynamic palmitoylation level of 15Q-HTT_1-1212_ WT or C1027/1028S was also measured in COS-7 cells by bio-orthogonal labeling assay following metabolic labeling with alkyne-palmitate (Figure 3C). The palmitoylation level of 15Q-HTT C1027/1028S was significantly increased compared to the WT control (Figure 3D; *t*-test: *p* = .0058). The higher palmitoylation turnover measured with the bio-orthogonal labeling assay, without any change of the total palmitoylation level with the ABE assay, could be explained by an increased palmitoylation dynamism at other HTT residues when C1027 and C1028 are mutated. This could be the consequence of conformational, location or protein-protein interaction changes in HTT C1027/1028S that would allow more efficient palmitoylation at other sites. Altogether, the data generated with these two palmitoylation assays support that the C1027 and C1028 residues are not palmitoylated.

Finally, we sought to identify new sites of palmitoylation using full-length HTT (Figure 1C). The palmitoylation level of full-length HTT WT, carrying a cysteine-to-serine mutation at C214, C3134 or C3144 was measured in HeLa cells by bio-orthogonal labeling assay following metabolic labeling with alkyne-palmitate (Figure 3E **;** uncropped blots displayed in Supplementary Figure S6). The palmitoylation level of HTT C214S was significantly decreased compared to the WT control (by 25%; 1-way ANOVA: *p* = .0024; Bonferroni’s test: WT vs. C214S, *p* = .0090) (Figure 3F). Palmitoylation levels of 15Q-HTT carrying the C3134S and C3144S were reduced compared to the WT control, by 25% and 30%, respectively (Bonferroni’s test: WT vs. C3134S, *p* = .0073; WT vs. C3144S, *p* = .0031). Therefore, these results suggest that cysteines 3134 and 3144 of HTT are dynamically palmitoylated. The palmitoylation level of full-length HTT WT, C214S, C3134S and C3144S could not be reliably detected using the IP-ABE assay in this experimental model, for unknown reasons (data not shown).

### 3.3 Full-length huntingtin is cleaved at D552 and post-translationally myristoylated at G553

**FIGURE 4:**
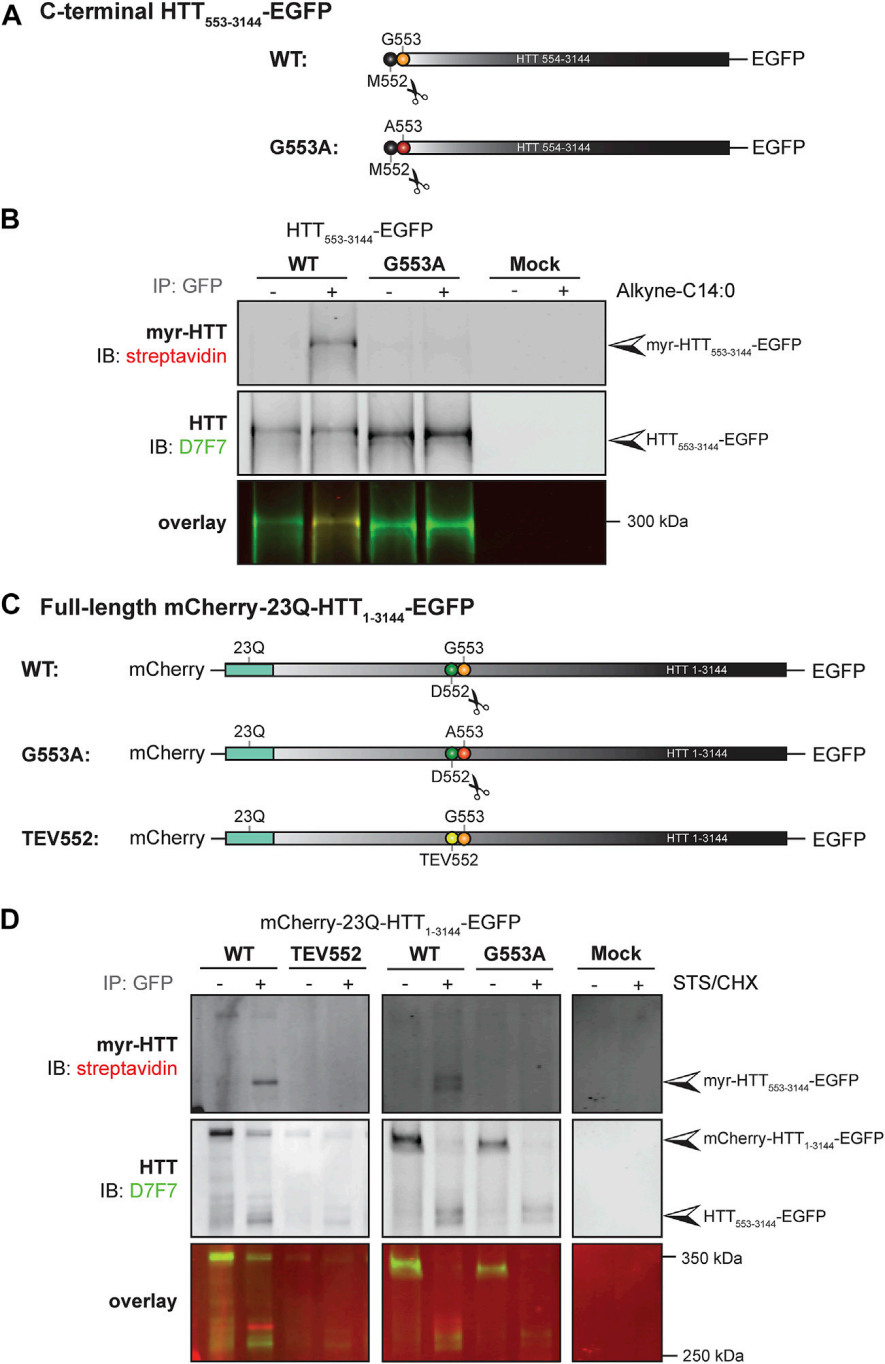
C-terminal HTT_553-3144_ is co- and post-translationally myristoylated at G553 in C-terminal HTT_553-3144_ and full-length HTT_1-3144_, respectively. **(A)** Schematic representation of the C-terminal HTT_553-3144_-EGFP constructs WT or carrying a glycine 5s53 to alanine (G553A). The scissors symbol indicates the cleavage of the N-terminal methionine M552, **(B)** HEK293 cells transfected with C-terminal HTT_553-3144_-EGFP WT or G553A mutation were labeled with DMSO or 100 µM alkyne-myristate for 4 h, **(C)** schematic representation of the full-length mCherry-23Q-HTT_1-3144_-EGFP constructs WT, G553A or with the D552 caspase cleavage site replaced with a Tobacco Etch Virus proteolytic site (TEV552). The scissors symbol indicates the D552 cleavage site, **(D)** HeLa cells were transfected with mCherry-23Q-HTT_1-3144_-EGFP constructs WT, G553A, or TEV552. Cells were labeled with 100 µM alkyne-myristate for 30 min, and then treated for 4 h with 1 µM staurosporine (STS) and 5 μg/mL cycloheximide (CHX) to induce proteolysis and inhibit protein synthesis, respectively. For both **(B, D)**, HTT fragments were immunoprecipitated from cell lysates using goat anti-GFP antibodies. The myristate analog was covalently linked to biotin azide through click chemistry. Myristoylated HTT_553-3144_-EGFP orthogonally labeled with biotin was detected with streptavidin and total HTT was detected with HTT antibodies (D7F7). Arrowheads indicate myristoylated HTT (myr-HTT_553-3144_-EGFP), total C-terminal HTT (HTT_553-3144_-EGFP) and full-length HTT (mCherry-HTT_1-3144_-EGFP). HTT_553-3144_-GFP myristoylation signal is not detected when the glycine 553 is mutated to alanine (G553A), or when the D552 cleavage site is blocked (TEV552). The images displayed in **(B, D)** are representative of three repetitions of each experiment. The western blot images in B are composites of different lanes from the same image for WT, G553A and mock, and a different image for WT and TEV552 as these constructs were expressed in independent experiments.

We previously demonstrated that a truncated version of HTT (HTT_1-588_-YFP) undergoes post-translational myristoylation at the newly exposed glycine 553 (G553) following caspase cleavage at D552 (Martin et al., 2014). To determine if longer C-terminal fragments of HTT can be myristoylated and detected *in vitro*, we first used a simplified experimental model allowing us to study HTT co-translational myristoylation, without inducing toxicity and proteolysis at D552 (Figures 4A, B; uncropped blots displayed in Supplementary Figure S7). Co-translational myristoylation of C-terminal HTT_553-3144_ was measured in HEK293 cells transiently expressing HTT_553_-_3144_-EGFP WT or carrying the G553A mutation and metabolically labeled with alkyne-myristate (Alkyne-C14:0) or DMSO (as a negative control) followed by click chemistry (Figure 4B). Myristoylation was detected in the WT form, but the G553A mutation entirely abrogates HTT myristoylation. The molecular weight of C-terminal HTT_553-3144_ G553A was slightly lower than that of C-terminal HTT_553-3144_ WT, which is consistent with what we observed previously with HTT_553-588_-YFP (Martin et al., 2014; Martin et al., 2018a; Martin et al., 2018b).

Our objective was then to fully characterize the post-translational myristoylation of full-length HTT (Figures 4C, D **;** uncropped blots displayed in Supplementary Figures S8). Myristoylation of C-terminal HTT_533-3144_ in HeLa cells exogenously expressing mCherry-23Q-HTT_1-3144_-EGFP WT, with the D552 caspase cleavage sites replaced with TEV proteolytic site (TEV552) or G553A, in the presence or absence of staurosporine (STS) which promotes caspase activity (Belmokhtar et al., 2001), was measured by bio-orthogonal labeling following metabolic labeling with alkyne-myristate (Figure 4D). The full-length mCherry-23Q-HTT_1-3144_-EGFP (indicated by the top arrowhead in the middle panel) was not orthogonally labeled with biotin, as expected (Martin et al., 2014). Myristoylated HTT_553-3144_ signal was detected when cells expressing the full-length HTT WT plasmid were treated with STS (myr-HTT_553-3144_-EGFP, indicated by an arrowhead in the top panel). A weaker signal was also detected in the absence of STS treatment, likely due to basal caspase activity, suggesting this is a constitutive PTM. Myristoylated HTT_553-3144_ was not detected when the D552 cleavage site of full-length HTT was replaced with a TEV proteolytic site nor in the presence of the G553A mutation. Altogether, the data confirms myristoylation of C-terminal HTT_553-3144_ at G553 after cleavage at D552 and shows for the first time that C-terminal HTT fragments longer than HTT_553-558_ undergo post-translational myristoylation, suggesting it occurs in endogenous HTT.

### 3.4 Huntingtin myristoylation increases when proteolytic cleavage of full-length huntingtin at D586 is impaired

Similar to palmitoylation, we have previously shown that a truncated version of HTT (HTT_1-588_-YFP) was less myristoylated in the presence of the HD mutation (Martin et al., 2014). Therefore, reduced myristoylation of huntingtin may be associated with increased toxicity of mutant HTT. Furthermore, we showed that blocking proteolysis of HTT_1-588_-YFP at D586 increases myristoylation at G553 (Martin et al., 2018b). Altogether, our data suggest that the protective effect of blocking proteolysis at D586 observed *in vitro* and *in vivo* (Graham et al., 2006; Graham et al., 2011; Wong et al., 2015) may be mediated, at least in part, through increased myristoylation at G553. Our aims were to assess the impact of the expanded polyQ tract and of D586 cleavage of full-length HTT on the myristoylation level of HTT_553-3144_.

**FIGURE 5:**
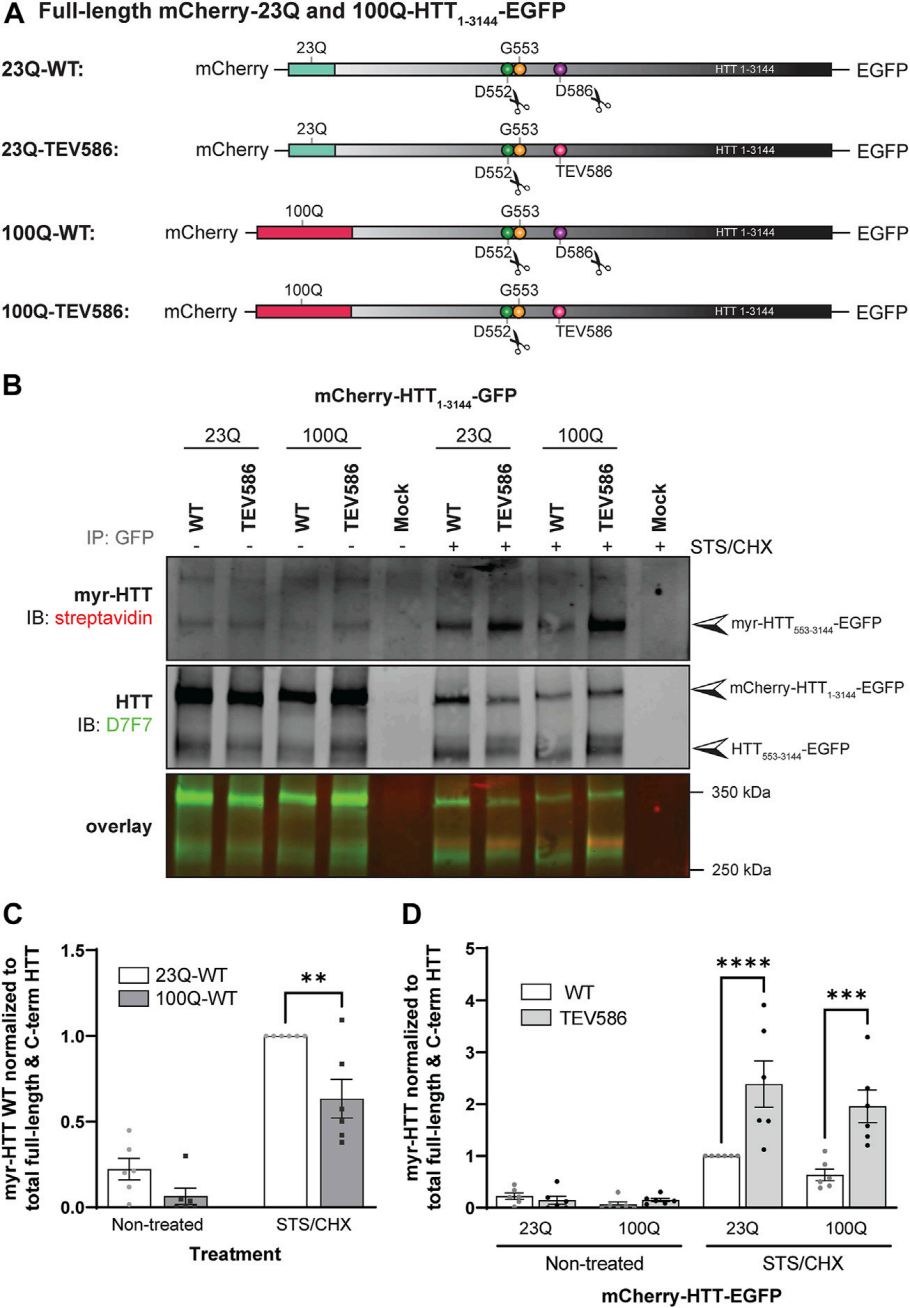
Myristoylation of C-terminal HTT_553-3144_ is reduced in the presence of the HD mutation, and increased when proteolytic cleavage of full-length huntingtin at D586 is impaired. **(A)** Schematic representation of the full-length mCherry-23Q and 100Q-HTT_1-3144_-EGFP constructs WT, or with the D586 caspase cleavage site replaced with a Tobacco Etch Virus proteolytic site (TEV586). The scissor symbols indicate the D552 and D586 cleavage sites, **(B)** HeLa cells were transfected with mCherry-23Q and mCherry-100Q-HTT_1-3144_-EGFP constructs WT or TEV586. Cells were metabolically labeled with 100 µM alkyne-myristate for 30 min in the absence or presence of 1 µM staurosporine and 5 μg/mL cycloheximide (STS/CHX) for 4 h to promote proteolysis and inhibit protein synthesis, respectively. Full-length and C-terminal HTT fragments were immunoprecipitated from cell lysates using goat anti-GFP antibodies. The myristate analog was covalently linked to biotin azide through click chemistry. Myristoylated HTT_553-3144_-EGFP orthogonally labeled with biotin was detected with streptavidin and total HTT was detected with HTT antibodies (D7F7). Arrowheads indicate myristoylated HTT (myr-HTT_553-3144_-EGFP) in the top panel, total C-terminal HTT (HTT_553-3144_-EGFP) and full-length HTT (mCherry-HTT_1-3144_-EGFP) in the middle panel, **(C)** myristoylation of HTT is reduced in the presence of the HD mutation. Myristoylation levels of HTT were quantified as the ratio of myristoylated HTT (myr-HTT_553-3144_-EGFP) normalized to the sum of full-length HTT (mCherry-HTT_1-3144_-EGFP) and C-terminal HTT fragments (HTT_553-3144_-EGFP), and expressed relatively to myristoylation of 23Q-HTT WT in cells treated with STS (n = 6), **(D)** Myristoylation of HTT is significantly increased when D586 cleavage of full-length HTT is impaired. Myristoylation of HTT was quantified as the ratio of myristoylated HTT normalized to the sum of full-length HTT and C-terminal HTT fragments, and expressed relatively to myristoylation of 23Q-HTT WT in cells treated with STS (n = 6). *Statistical analysis*: **(C)** 2-way ANOVA: interaction, *p* = .15; STS/CHX treatment, ****p < .0001; HD mutation, **p = .0011. Sidak’s multiple comparison test, **p = .0025. **(D)** 3-way ANOVA: WT vs. TEV586, ****p < .0001; Non-treated vs. STS/CHX, ****p < .0001; 23Q vs. 100Q, *p* = .10. Sidak’s multiple comparison test (WT vs. TEV586), ****p < .0001, ***p < .0001.2-way ANOVA (TEV586 constructs only): interaction, *p* = .44; Non-treated vs. STS/CHX, ***p < .0001; 23Q vs. 100Q, *p* = .79.

Post-translational myristoylation of HTT was assessed in HeLa cells exogenously expressing full-length mCherry-23Q or 100Q-HTT_1-3144_-EGFP WT or with the D586 caspase cleavage sites replaced with TEV proteolytic sites (TEV586) in the presence or absence of STS to promote proteolysis, using the bio-orthogonal labeling assay (Figures 5A, B **;** uncropped blots displayed in Supplementary Figure S9). Again, post-translationally myristoylated C-terminal HTT_533-3144_ was detected in all conditions, but significantly increased in the presence of STS, supporting that this PTM is constitutive, after basal caspase activity (Figure 5B). Post-translational myristoylation of C-terminal HTT_553-3144_ was significantly reduced by 30% in mutant 100Q-HTT_1-3144_ compared to the wild-type control (23Q-HTT_1-3144_) in the presence or absence of STS (Figure 5C; 2-way ANOVA: HD mutation, *p* = .0011). Post-translational myristoylation of C-terminal HTT_533-3144_ was significantly increased when D586 cleavage of full-length 23Q and 100Q-HTT_1-3144_ was impaired (TEV586) compared to fully-cleavable 23Q and 100Q-HTT_1-3144_ WT (Figure 5D; 3-way ANOVA: WT vs. TEV586, *p* < .0001). The presence of the HD mutation did not significantly alter the myristoylation level of 100Q-HTT_1-3144_ TEV586, compared to 23Q-HTT_1-3144_ TEV586 (Figure 5B; 2-way ANOVA: HD mutation, *p* = .79). These results confirm that blocking full-length HTT cleavage at D586 promotes HTT_533-3144_ myristoylation at G553, and normalizes the effect of the HD mutation on HTT_533-3144_ myristoylation.

### 3.5 Huntingtin post-translational myristoylation promotes the co-interaction between C-terminal and N-terminal HTT fragments

It has previously been shown that C-terminal domains of HTT can interact with N-terminal HTT after proteolysis at single sites (Ochaba et al., 2014; El-Daher et al., 2015). In addition, C-terminal HTT alone appears to be toxic, particularly when HTT is processed at multiple sites, which decreases the interaction between the N- and C-termini (El-Daher et al., 2015). Our next goal was to investigate the impact of HTT myristoylation on the binding affinity between N-terminal and C-terminal HTT fragments, as we hypothesized that myristoylated fragments of HTT induce autophagy by impacting HTT protein-protein interaction.

**FIGURE 6:**
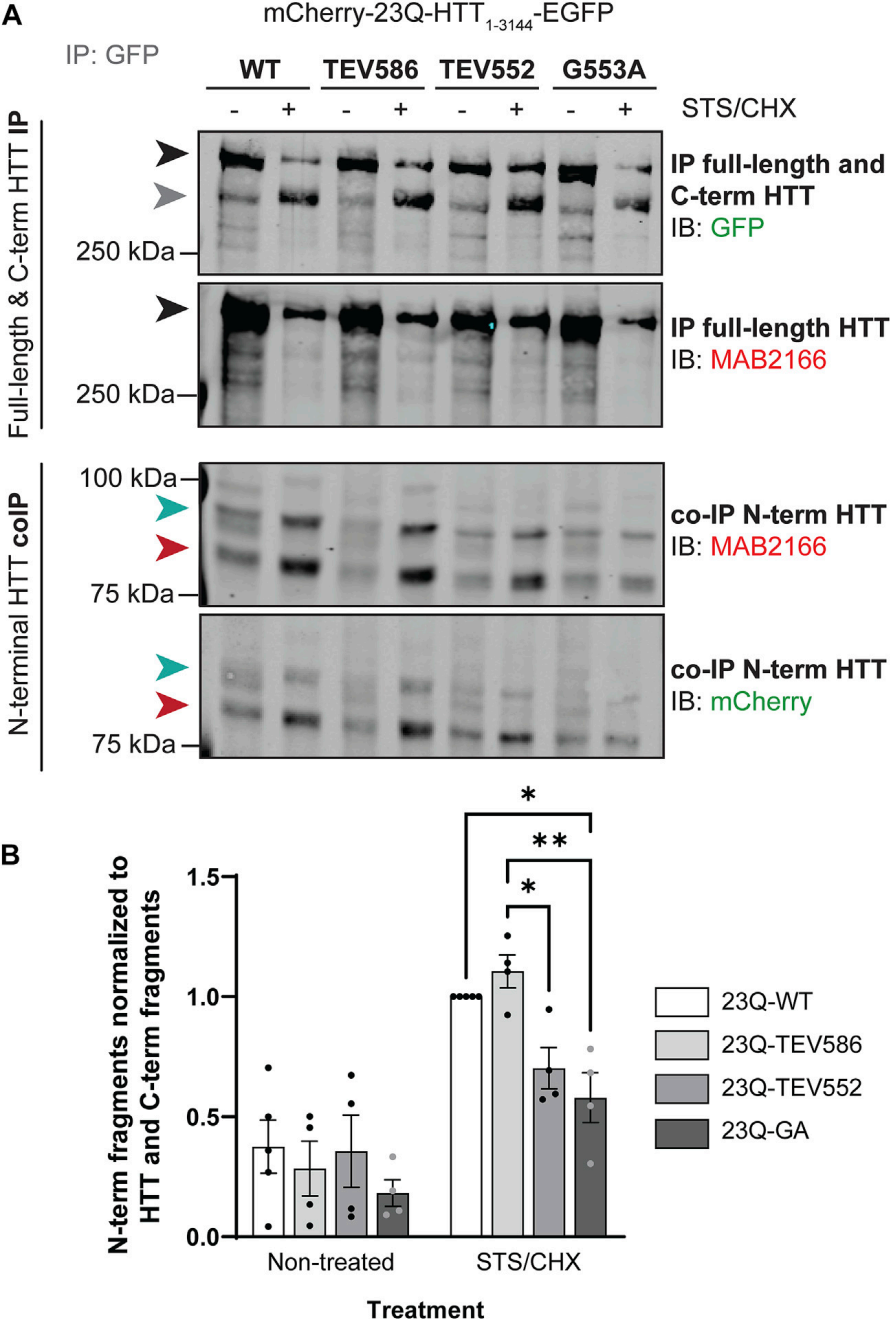
Blocking post-translational myristoylation in full-length wild-type HTT reduced the interaction between the N- and C-terminal fragments. **(A)** HeLa cells transiently expressing mCherry-23Q-HTT_1-3144_-EGFP WT, TEV586, TEV552 or G553A for 46 h were treated for 2 h with 5 µM STS and 5 μg/mL cycloheximide (STS/CHX) to induce proteolysis and inhibit protein synthesis, respectively. HTT-EGFP was immunoprecipitated from lysates with goat anti-GFP antibodies. Immunoblots were conducted with mouse anti-HTT (MAB2166; amino acid 443–457) and rabbit anti-GFP antibodies. Then, the membranes were stripped and re-probed with rabbit anti-mCherry antibodies. Full-length mCherry-23Q-HTT_1-3144_-EGFP, detected with GFP or MAB2166 antibodies, is indicated by a black arrowhead. C-terminal HTT-EGFP fragments, detected with GFP antibodies, are indicated by a grey arrowhead. The green and red arrowheads indicate the two main N-terminal mCherry-23Q-HTT fragments co-immunoprecipitated and detected by both mCherry and MAB2166 antibodies, with an apparent molecular weight of ∼82 and 90 kDa, respectively, **(B)** interaction between N-terminal HTT fragments with full-length and C-terminal HTT was quantified as the ratio of the co-immunoprecipitated N-terminal HTT fragments detected with mCherry antibodies with molecular weights between 75 and 100 kDa normalized to the sum of immunoprecipitated full-length HTT and all C-terminal HTT fragments detected with GFP antibodies, and expressed relatively to 23Q-HTT WT in cells treated with STS (n = 4). *Statistical analysis*: 2-way ANOVA: interaction, *p* = .076; STS/CHX treatment, ****p < .0001; PTM mutation, **p = .0076. Tukey’s multiple comparisons test: 23Q-WT vs. 23Q-G553A, *p = .017; 23Q-TEV586 vs. 23Q-TEV552, *p = .033; 23Q-TEV586 vs. 23Q-G553A, **p = .0038.

The co-interaction between C- and N-terminal HTT was investigated in HeLa cells transiently expressing mCherry-23Q-HTT_1-3144_-EGFP WT, TEV586, TEV552 or G553A, after treatment with STS to induce proteolysis, by co-immunoprecipitation (Figure 6 **;** uncropped blots displayed in Supplementary Figure S10). Full-length mCherry-23Q-HTT_1-3144_-EGFP (black arrowheads) and C-terminal HTT-EGFP fragments (grey arrowhead) generated by proteolysis were immunoprecipitated from lysates using GFP antibodies, and N-terminal HTT fragments co-immunoprecipitated were detected by western blot analysis using mCherry and N-terminal HTT antibodies (Figure 6A). Two main N-terminal HTT fragments with a molecular weight of ∼82 (red arrowheads) and 90 kDa (green arrowheads) detected by both mCherry and MAB2166 (amino acid ∼443–457) antibodies were co-immunoprecipitated with C-terminal fragments and full-length mCherry-HTT_1-3144_-EGFP. Additionally, co-immunoprecipitated N-terminal fragments with a molecular weight of 85, 100 and 115 kDa were detected at a lower level. This data confirm the interaction between N- and C-terminal HTT previously described (Palidwor et al., 2009; Ochaba et al., 2014; El-Daher et al., 2015). The amount of co-immunoprecipitated N-terminal HTT fragments (tagged with mCherry) with a molecular weight of between 75 and 100 kDa normalized to the level of full-length and C-terminal HTT (tagged with GFP) in non-treated or STS-treated cells is displayed in Figure 6B. Impairing post-translational myristoylation of full-length HTT_1-3144_ at G553 significantly reduced the co-interaction between the N-terminal fragments with full-length and C-terminal HTT compared to HTT WT and TEV586 in the STS-treated cells (2-way ANOVA, PTM mutation, *p* = .0076; Tukey’s post-test, WT vs. G553A, *p* = .017; TEV586 vs. G553A, *p* = .0038). Blocking proteolysis of full-length HTT at D552, which also blocks post-translational myristoylation, also significantly reduced C- an N-terminal fragment co-interaction compared to HTT TEV586, in STS-treated cells (Tukey’s post-test, TEV586 vs. TEV552, *p* = .033; *t*-test: WT vs. TEV552, *p* = .0056). This result suggests that post-translational myristoylation of HTT promotes the interaction between N-terminal and C-terminal HTT fragments in this model, which could contribute to the protective effect of blocking proteolysis at D586 in HD.

## 4 Discussion

Proper cellular localization of proteins is crucial to their function and is particularly important in neurons that require proteins to traffic long distances along axons and complex dendrites. Mislocalization of neuronal proteins can lead to proteostasis deficiencies and toxicity. Consequently, altered palmitoylation and myristoylation have been implicated in many psychiatric and neurodegenerative disorders (Sanders et al., 2015; Cho and Park, 2016; Pinner et al., 2016; Philippe and Jenkins, 2019; Seo et al., 2022). While targeting acyltransferase enzymes and their substrate proteins could be a promising approach to treat neurological disorders, there are still many unanswered questions that need to be addressed regarding the role of fatty acylation on protein trafficking and targeting to specific neuronal locations, or more generally on brain health and functions (Ji and Skup, 2021; Petropavlovskiy et al., 2021).

We have shown that the palmitoylation level of HTT and many synaptic proteins are reduced in HD (Yanai et al., 2006; Lemarié et al., 2021). Modulating palmitoylation by inhibiting depalmitoylating APTs is protective in HD cells (immortalized cell lines, primary neurons, iPSC-derived neurons) and in the *Hdh* ^CAG140/+^ knock-in mouse model (Lemarié et al., 2021; Virlogeux et al., 2021). However, the biological functions of HTT fatty acylation and the role that loss of HTT fatty acylation plays on disease progression are still not fully elucidated.

Only one site of palmitoylation was previously identified at C214 using radioactive labeling techniques (Yanai et al., 2006). With recent advances in the detection of palmitoylation, it has become apparent that there are likely additional palmitoylation sites within HTT. The exhaustive identification of fatty palmitoylation sites within HTT is essential to allow us to identify the specific sites that mediate protection in HD in the presence of depalmitoylation inhibitors.

Here, we investigated the existence of additional palmitoylation sites within the full-length HTT protein, by transiently expressing HTT carrying cysteine-to-serine mutations at sites predicted to be palmitoylated (CSS-Palm 3.0 prediction program; Figure 1) and by measuring HTT palmitoylation levels using the IP-ABE and bio-orthogonal labeling assays (Figures 2 and 3). Within the N-terminal region of HTT, our data support that C105 and C433 of HTT are palmitoylated (Figures 2A-D). This conclusion is supported by high throughput screening by resin-assisted capture of S-acylated proteins (acyl-RAC) followed by LC-MS/MS that identified these two residues as S-palmitoylation and S-nitrosylation (addition of nitric oxide (NO)) sites (Ni et al., 2016). The residues C109, C152, C204 and C280 were considered as palmitoylated in the Ni *et al.* study 2016. However, only C280 appeared in the CSS-Palm 3.0 prediction list with a low score (Figure 1A), and was therefore not investigated by low throughput palmitoylation assay. We observed an additive effect of the mutations of C105, C214 and C433 on HTT_1-548_ palmitoylation level, although HTT palmitoylation was still not entirely abrogated (Figures 2E, F). Blocking palmitoylation at C105, C214 and C433 may be compensated by palmitoylation at alternate sites in close proximity (C109 for C105 and C204 for C214) a phenomenon that has been observed for other proteins, including the voltage-gated sodium channel Nav1.6 (Pan et al., 2020).

In the middle region of the HTT protein, the cysteine residues 1027 and 1028 have a high palmitoylation prediction score according to the CSS-Palm 3.0 program (Figure 1A). Both residues were mutated to serine in case these adjacent cysteines were interchangeably palmitoylated. The double cysteine-to-serine mutations C1027/1028S did not significantly alter HTT_1-1212_ palmitoylation level using the IP-ABE assay (Figures 3A, B), but surprisingly increased HTT dynamic palmitoylation level when measured with the bio-orthogonal labeling assay (Figures 3C, D). This unexpected increase in palmitoylation turnover measured with the bio-orthogonal labeling assay, without any change of the global palmitoylation level with the ABE assay, could be due to an indirect effect of the C1027/1028 residue mutation which may impact HTT conformation, localization, or protein-protein interaction, and in doing so could promote palmitoylation of a nearby residue, such as C944 which was predicted to be palmitoylated (Ni et al., 2016). Alternatively, the significant increase in palmitoylation observed with the bio-orthogonal labeling assay may be indicative of transacylation (or catalytic cysteine palmitoyl relay (CCPR)) of HTT and these sites may serve as recipients of palmitate from other sites on HTT, similar to mitochondrial HMG-CoA synthase (Kostiuk et al., 2010). However, these two cysteines were not identified as palmitoylation sites in the acyl-RAC high throughput performed in the Ni et al., 2016 study. Therefore, they are unlikely to be new sites of palmitoylation, but this highlights an important issue when trying to identify new sites of palmitoylation.

In the C-terminal region of HTT, C3134 and C3144 were investigated as potential new palmitoylation sites. Palmitoylation of full-length 15Q-HTT C3134S and C3144S was decreased compared to the WT control to the same extent than the C214S mutation, suggesting that cysteines 3134 and 3144 of HTT are dynamically palmitoylated (Figures 3E, F). Of note, when expressing full-length HTT, our method also measures endogenous HTT palmitoylation. This suggests that the effect of blocking palmitoylation of exogenous HTT at these sites may be greater than what we measured.

To summarize, we have identified multiple new palmitoylation sites of huntingtin at C105, C433, C3134 and C3144. It will be important to confirm the palmitoylation of these residues by mass spectrometry. Investigating the effects of these palmitoylation sites on wild-type and mutant HTT localization, clearance, aggregation or protein trafficking will be necessary to further decipher the biological functions of HTT palmitoylation. Additionally, because proteins that contain multiple palmitoylation sites can be modified at different intracellular locations by various PATs (Tian et al., 2010; Kang et al., 2019), it will be relevant to assess if additional enzymes, aside from ZDHHC17 and 13, are involved in the palmitoylation of these residues.

Myristoylation has been shown to be essential for the function of many proteins and is required for cell survival (Martin D. D. O et al., 2011; Thinon et al., 2016). Previously, using truncated forms of HTT, we showed that HTT is post-translationally myristoylated at G553 following caspase-cleavage (Martin et al., 2014). Like HTT palmitoylation, post-translational myristoylation of HTT_1-588_ is significantly decreased in the presence of the polyQ expansion (Martin et al., 2014). We also found that the myristoylated HTT_553–585_ fragment induces the formation of autophagosomes, and thereby plays a role in the initiation of autophagy, a process altered in HD (Martinez-Vicente and Cuervo, 2007; Krainc, 2010; Martinez-Vicente et al., 2010; Ochaba et al., 2014; Menzies et al., 2015; Ehrnhoefer et al., 2018). Furthermore, our data show that blocking proteolysis of HTT_1-588_ at D586 increases myristoylation at G553 (Martin et al., 2018b). This suggests that the protective effect of blocking proteolysis at D586 (Graham et al., 2006; Graham et al., 2011; Wong et al., 2015) may be mediated, at least partially, through increased myristoylation at G553. This potential crosstalk between D586 cleavage and G553 myristoylation could explain the rescue of autophagy dysregulation in the C6R mouse model (YAC128 line carrying a mutation preventing proteolysis at D586) compared to the YAC128 mouse line (Ehrnhoefer et al., 2018). Developing tools to characterize post-translational myristoylation in full-length HTT is now essential to confirm our findings.

For the first time, co- and post-translational myristoylation of C-terminal HTT_533-3144_ was detected in human cells exogenously expressing HTT_533-3144_-EGFP WT (Figure 4B), mCherry-23Q-HTT_1-3144_-EGFP WT (Figure 4D) and mCherry-23Q and 100Q-HTT_1-3144_-EGFP WT and D586 proteolysis-resistant (TEV586) (Figure 5B), in the presence or absence of STS to induce proteolysis. Myristoylation of HTT in the absence of stressors suggests that HTT is likely constitutively post-translationally myristoylated endogenously. The presence of the G553A or the D552 proteolysis-resistant (TEV552) mutations entirely abrogated the myristoylation of HTT_533-3144_. Consistent with shorter N-terminal fragments of HTT, myristoylation of HTT_533-3144_ was reduced in the presence of the HD mutation (Figure 5C). In contrast, post-translational myristoylation of C-terminal HTT_553-3144_-EGFP was significantly increased when the cleavage of full-length HTT at D586 was impaired (TEV586) (Figure 5D). This result confirms our hypothesis that blocking HTT cleavage at D586 promotes HTT myristoylation at G553, and supports the PTM crosstalk we proposed in Ehrnhoefer et al., 2018 study.

A role for HTT in the regulation of autophagy has been described (Steffan, 2010; Ochaba et al., 2014; Rui et al., 2015; Ashkenazi et al., 2017a; Ashkenazi et al., 2017b), with both the HTT N- and C-termini playing different but inter-dependent roles in autophagy (Ochaba et al., 2014). This process may be promoted by the interaction of the two halves after proteolysis (Palidwor et al., 2009; El-Daher et al., 2015). C-terminal HTT alone appears to be toxic, particularly when HTT is processed at multiple sites (D586, D513 and D167), which decreases the interaction between the N- and C-termini (El-Daher et al., 2015). C-terminal HTT toxicity involves inactivation of dynamin 1 at ER membrane and ER dilatation and stress (El-Daher et al., 2015). Impairing myristoylation of wild-type 23Q-HTT_1-3144_ significantly decreased the interaction of two N-terminal HTT fragments (∼82 and 90 kDa) with C-terminal HTT_553-3144_ compared to the WT control and the TEV586 (Figure 6).

This result suggests that myristoylation of HTT promotes the interaction between C-terminal HTT_553-3144_ and N-terminal HTT fragments. Thus, the protective effect of blocking cleavage of full-length HTT at D586 may be mediated through promoting proteolysis at D552 and post-translational myristoylation of HTT, thereby maintaining the interaction between the toxic N- and C-termini. These N- and C-terminal fragments can then potentially be directed to the autophagosome for degradation. Altogether, the data support our hypothesis that targeting myristoylation could be beneficial in the context of HD. It would be relevant to modulate myristoylation levels of HTT to assess the impact on N- and C-terminal fragments co-interaction and clearance. Inhibitors of NMTs, which impair protein myristoylation, have been developed to treat cancer cells (Thinon et al., 2016; Beauchamp et al., 2020; Sangha et al., 2022) and various parasitic protozoa (Khalil et al., 2019). However, the irreversible nature of myristoylation makes it a more challenging target compared to palmitoylation, specifically if the goal is to promote its levels.

Our study reveals new palmitoylation sites of HTT and new insights into the regulation of HTT by myristoylation. Myristoylation often regulates protein anchoring to the membrane via second signals such as palmitoylation (Resh, 2016; Seo et al., 2022), and we can hypothesize that acylation cascades may impact wild-type HTT functions and mutant HTT toxicity. Moreover, some of the new palmitoylation sites identified in this study are located in clusters of PTMs of HTT that modulate mutant HTT functional or toxic properties (Arbez et al., 2017), and could therefore impact key PTMs of HTT. Determining the physiological relevance of these new palmitoylation sites, and the interplay between different PTMs of HTT will help develop new therapeutic strategies to treat HD.

## Data Availability

The raw data supporting the conclusion of this article will be made available by the authors, without undue reservation.

## Supplementary material

Supplementary Figure S1 Uncropped blots used for Figure 2A displaying total (MAB2166/green channel) and palmitoylated HTT (Streptavidin/red channel) measured by IP-ABE assay in COS-7 cells transiently expressing 15Q-HTT1-548 WT, C214S, C105S or C433S.

Supplementary Figure S2 Uncropped blots used for Figure 2C displaying total (MAB2166/green channel) and palmitoylated HTT (Streptavidin/red channel) measured by bio-orthogonal labeling assay in COS-7 cells transiently expressing 15Q-HTT1-548 WT, C214S, C105S or C433S.

Supplementary Figure S3 Uncropped blots used for Figure 2E displaying total (MAB2166/green channel) and palmitoylated HTT (Streptavidin/red channel) measured by IP-ABE assay in COS-7 cells transiently expressing 15Q-HTT1-548 WT, C214S or C105/214/433S.

Supplementary Figure S4 Uncropped blots used for Figure 2F displaying total (MAB2166/green channel) and palmitoylated HTT (Streptavidin/red channel) measured by bio-orthogonal labeling assay in COS-7 cells transiently expressing 15Q-HTT1-548 WT, C214S or C105/214/433S.

Supplementary Figure S5 Uncropped blots used for Figure 3A and 3C displaying total (MAB2166/green channel) and palmitoylated HTT (Streptavidin/red channel) measured by IP-ABE or bio-orthogonal labeling assays in COS-7 cells transiently expressing 15Q-HTT1-1212 WT or C1027/1028S.

Supplementary Figure S6 Uncropped blots used for Figure 3E displaying total (D7F7/green channel) and palmitoylated HTT (Streptavidin/red channel) measured by bio-orthogonal labeling assay in HeLa cells transiently expressing 15Q-HTT1-3144 WT, C214S, C3134S or C3144S.

Supplementary Figure S7 Uncropped blots used for Figure 4B displaying total (D7F7/green channel) and myristoylated HTT (Streptavidin/red channel) measured by bio-orthogonal labeling assay in HEK293 cells transfected with C-terminal HTT553-3144-EGFP WT or G553A.

Supplementary Figure S8 Uncropped blots used for Figure 4D displaying total (D7F7/green channel) and myristoylated HTT (Streptavidin/red channel) measured by bio-orthogonal labeling assay in HeLa cells transfected with mCherry-23Q-HTT1-3144-EGFP constructs WT, G553A, or TEV552.

Supplementary Figure S9 Uncropped blots used for Figure 5B displaying total (D7F7/green channel) and myristoylated HTT (Streptavidin/red channel) measured by bio-orthogonal assay in HeLa cells were transfected with mCherry-23Q and mCherry-100Q-HTT1-3144-EGFP constructs WT or TEV586.

Supplementary Figure S10 Uncropped blots used for Figure 6A displaying immunoprecipitated full length and C-term HTT (GFP/green channel; MAB2166/red channel) and co-immunoprecipitated N terminal HTT (MAB166/red channel; mCherry/green channel) in HeLa cells transiently expressing mCherry-23Q-HTT1-3144-EGFP WT, TEV586, TEV552 or G553A.

## Glossary

Acyl-RAC: acyl-resin-assisted capture
APT: acyl-protein thioesterase
BSA: bovine serum albumin
C6R: caspase-6 resistant
CCPR: catalytic cysteine palmitoyl relay
CHX: cycloheximide
DTT: dithiothreitol
DMEM: Dulbecco’s modified Eagle medium
EGFP: enhanced green fluorescent protein
ER: endoplasmic reticulum
FBS: fetal bovine serum
HIP14: huntingtin interacting protein 14
HIP14L: huntingtin interacting protein 14-like
HTT: huntingtin
HD: Huntington disease
HAM: hydroxylamine
IB: immunoblot
IP: immunoprecipitation
IP-ABE: acyl-biotin exchange assay on immunoprecipitated proteins
NEM: N-ethylmaleimide
NMT: N-myristoyltransferase
NO: nitric oxide
PMSF: phenylmethylsulfonyl fluoride
PBS: phosphate-buffered saline
PolyQ: polyglutamine
PTM: post-translational modification
SDS: sodium dodecyl sulfate
STS: staurosporine
TEV: Tobacco Etch virus
TBTA: tris (benzyltriazolylmethyl)amine
TCEP: tris-carboxyethylphosphine
WT: wild-type
ZDHHC17: Zinc finger DHHC-type palmitoyltransferase 17
ZDHHC13: Zinc finger DHHC-type palmitoyltransferase 13
