# Supplementary figures and images for: Full-length huntingtin is palmitoylated at multiple sites and post-translationally myristoylated following caspase-cleavage
Source: Front Physiol. 2023 Jan 13;14:1086112. doi: 10.3389/fphys.2023.1086112 (PMC9880554; doi:10.3389/fphys.2023.1086112)

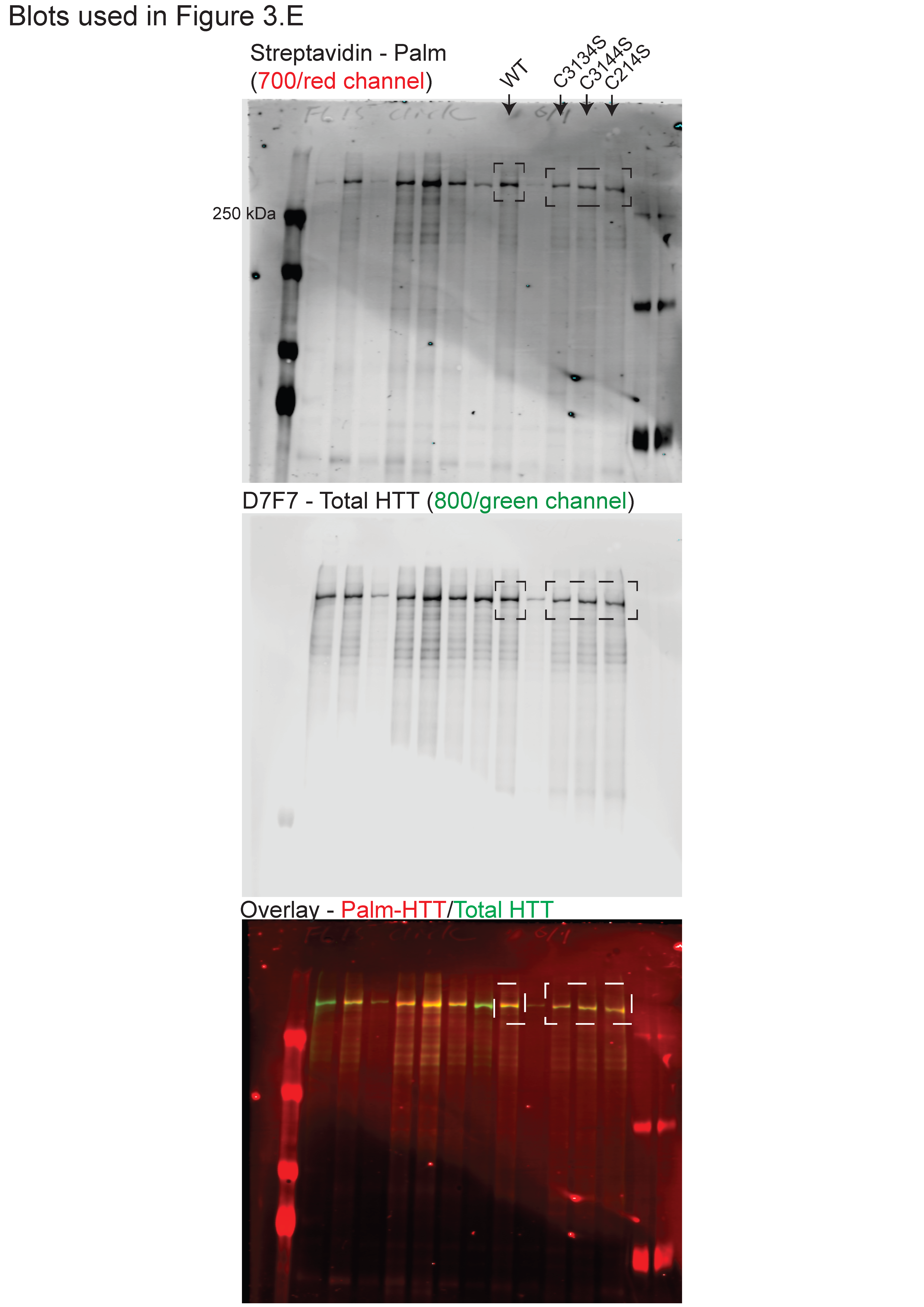

Supplement: Supplementary file 1 [file Image6.TIF]

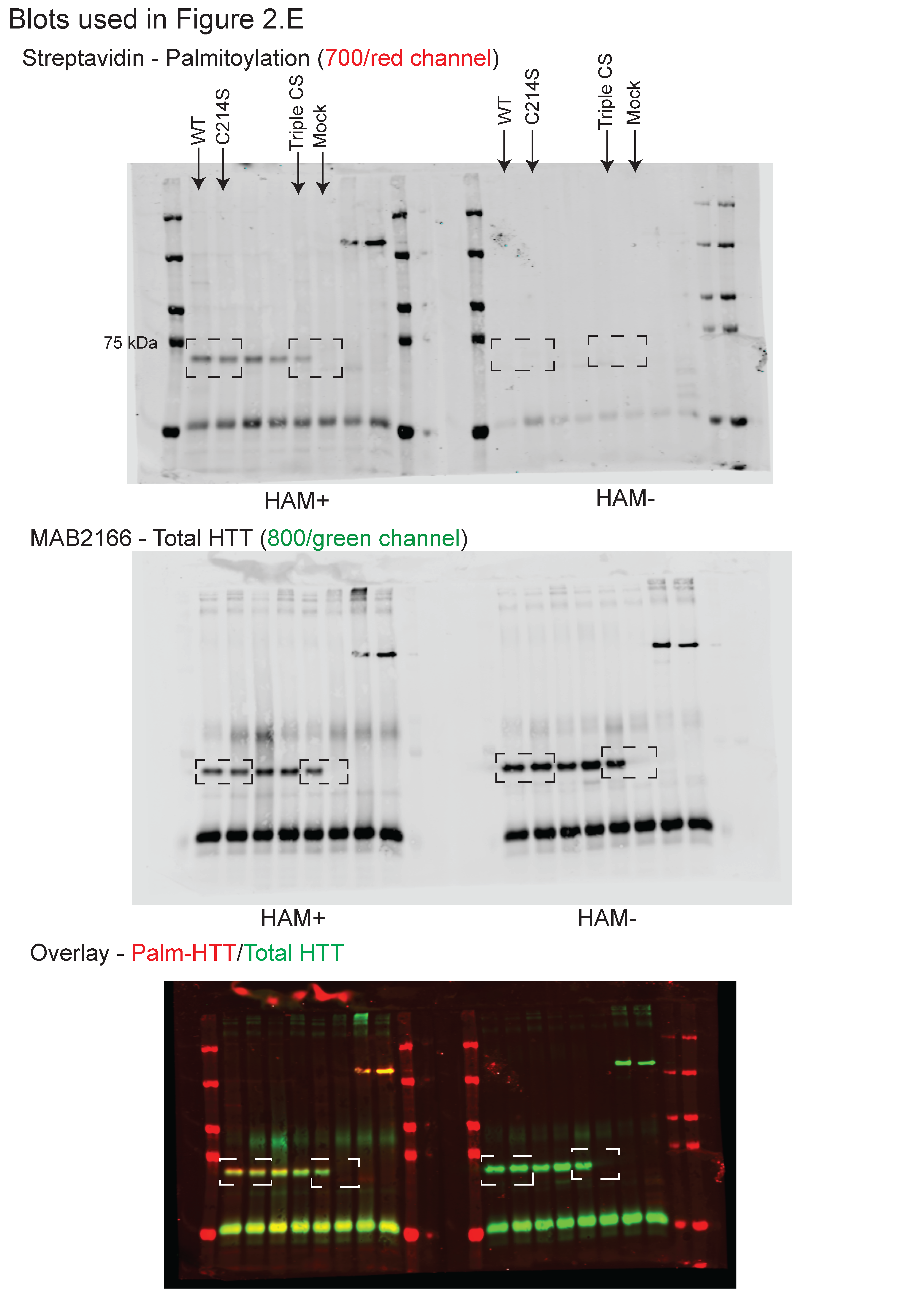

Supplement: Supplementary file 2 [file Image3.TIF]

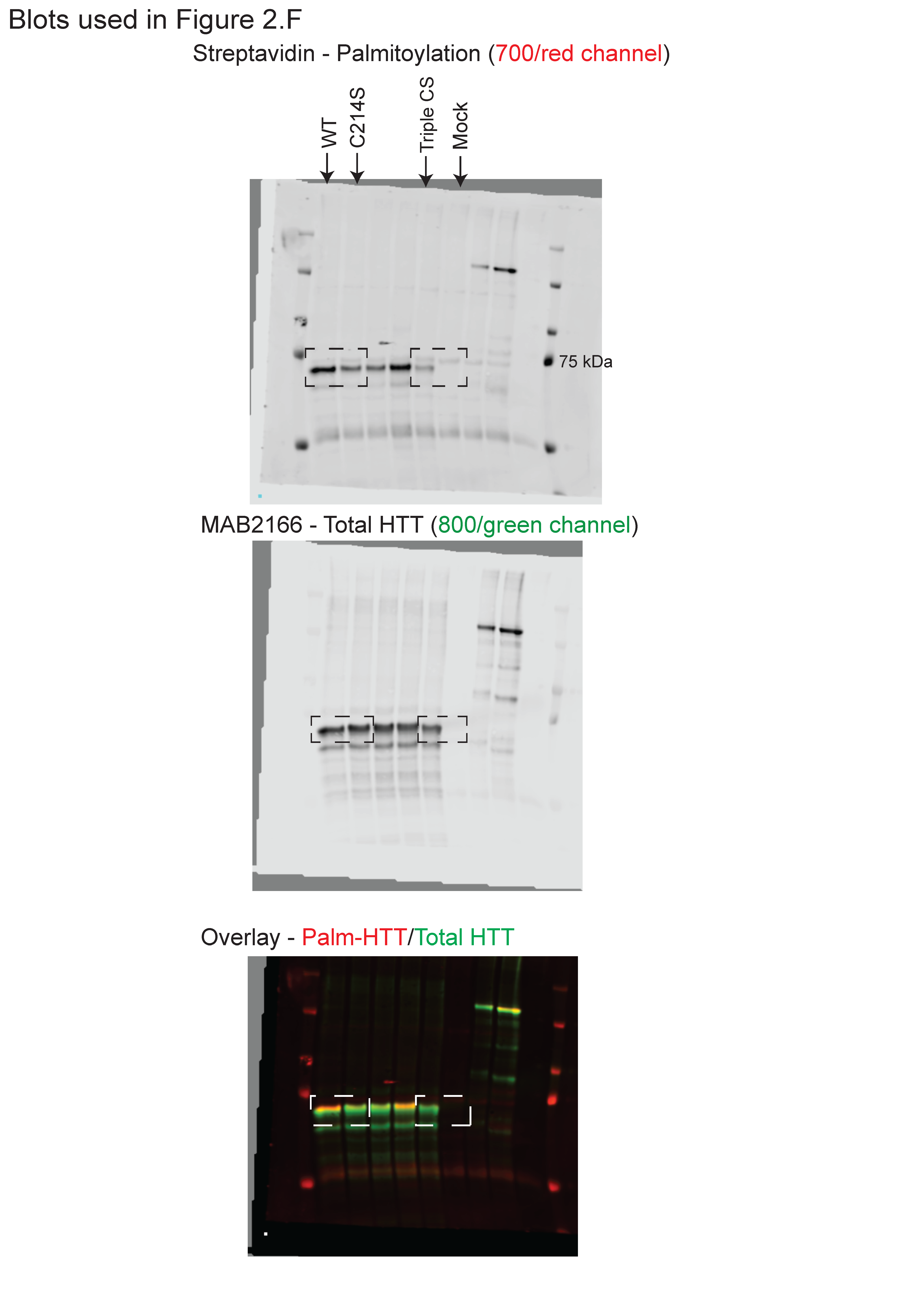

Supplement: Supplementary file 3 [file Image4.TIF]

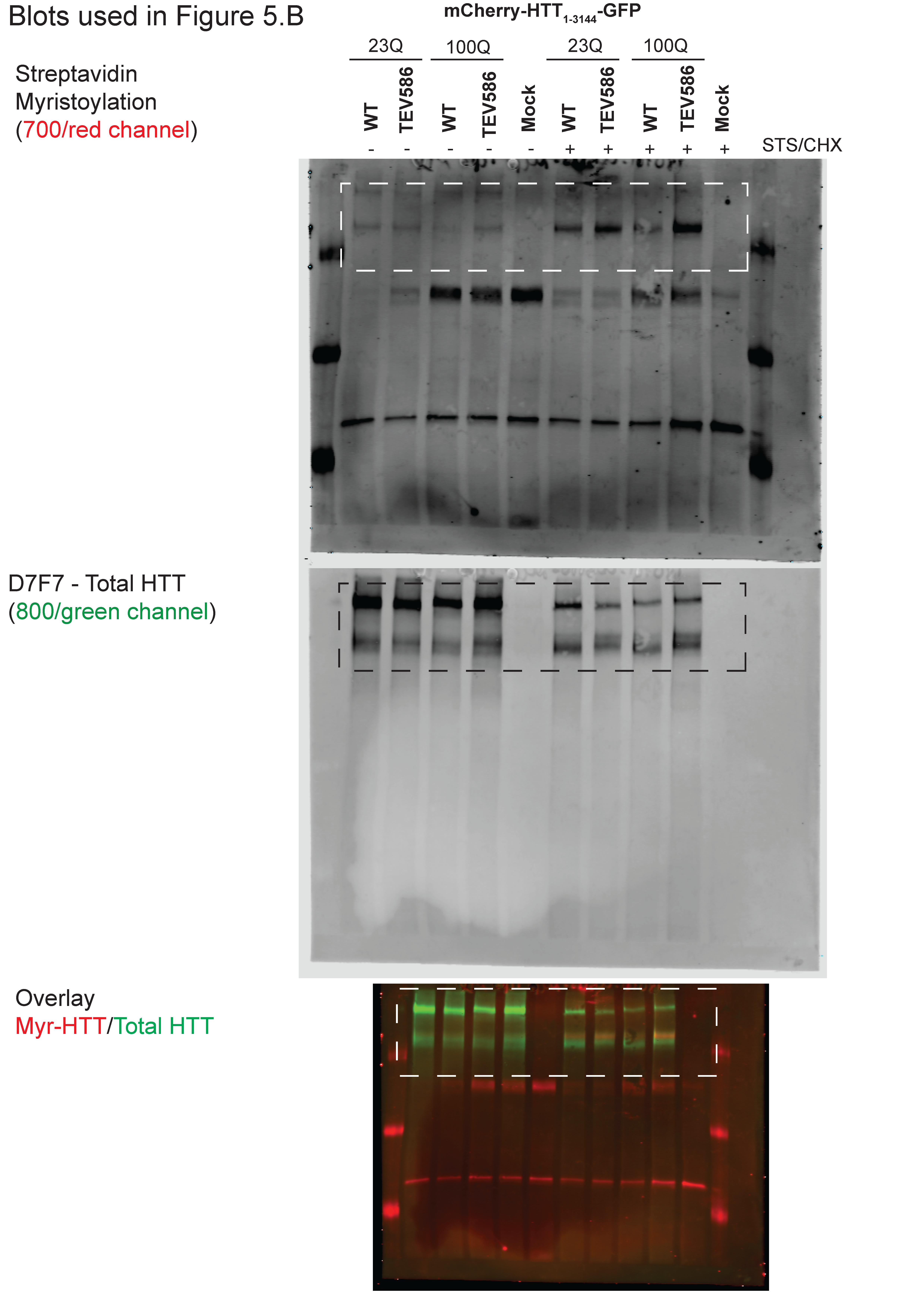

Supplement: Supplementary file 4 [file Image9.TIF]

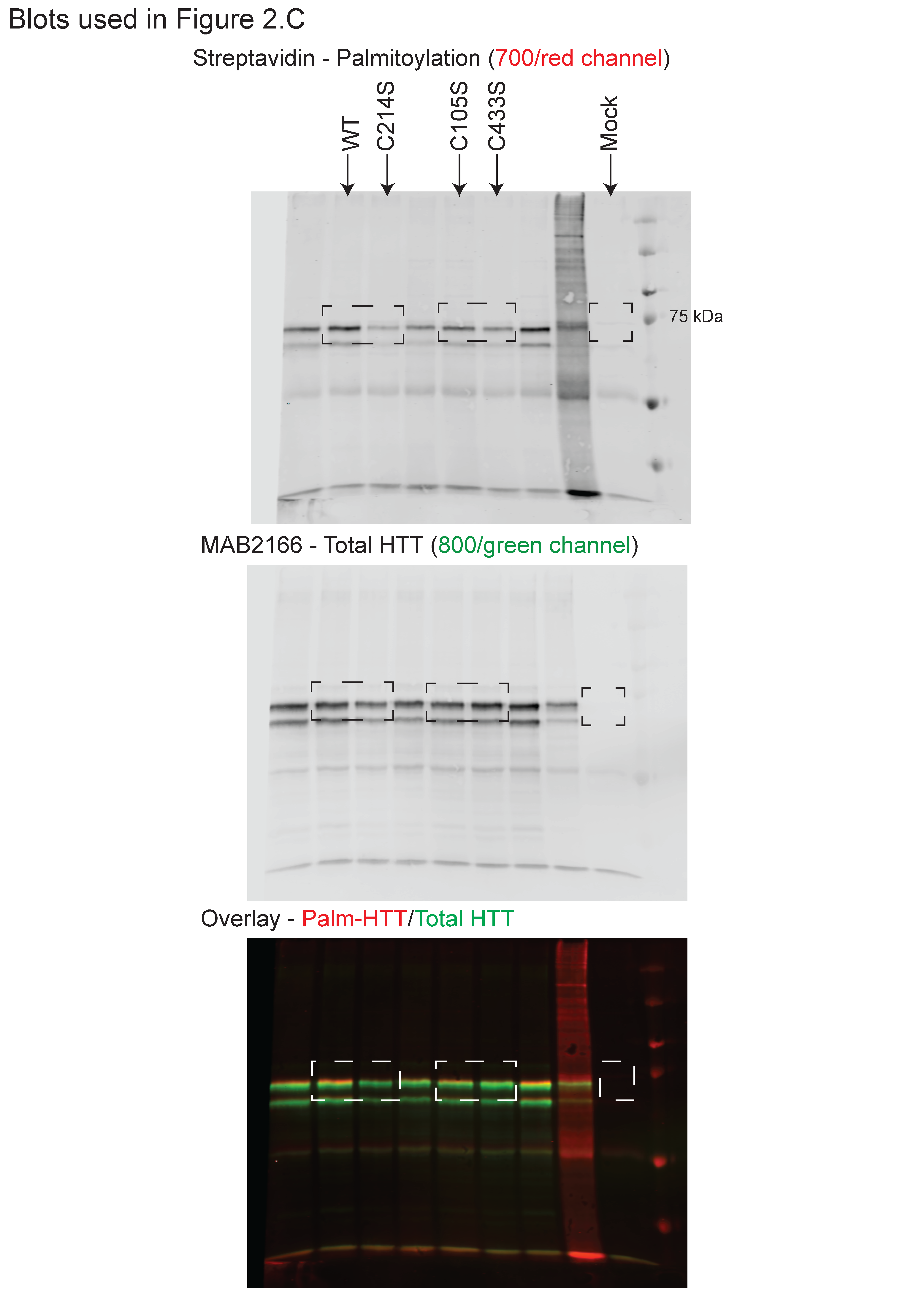

Supplement: Supplementary file 5 [file Image2.TIF]

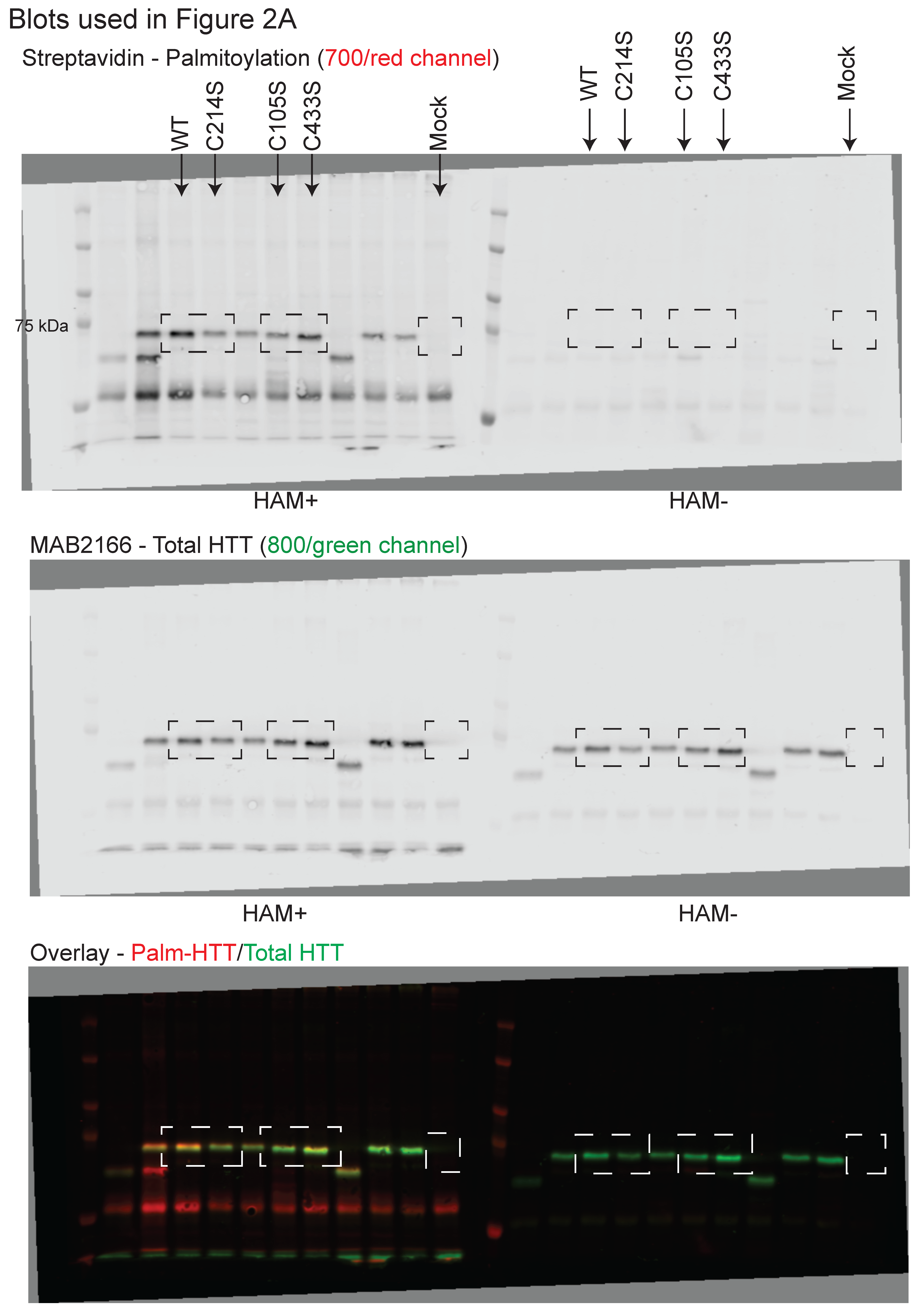

Supplement: Supplementary file 6 [file Image1.TIF]

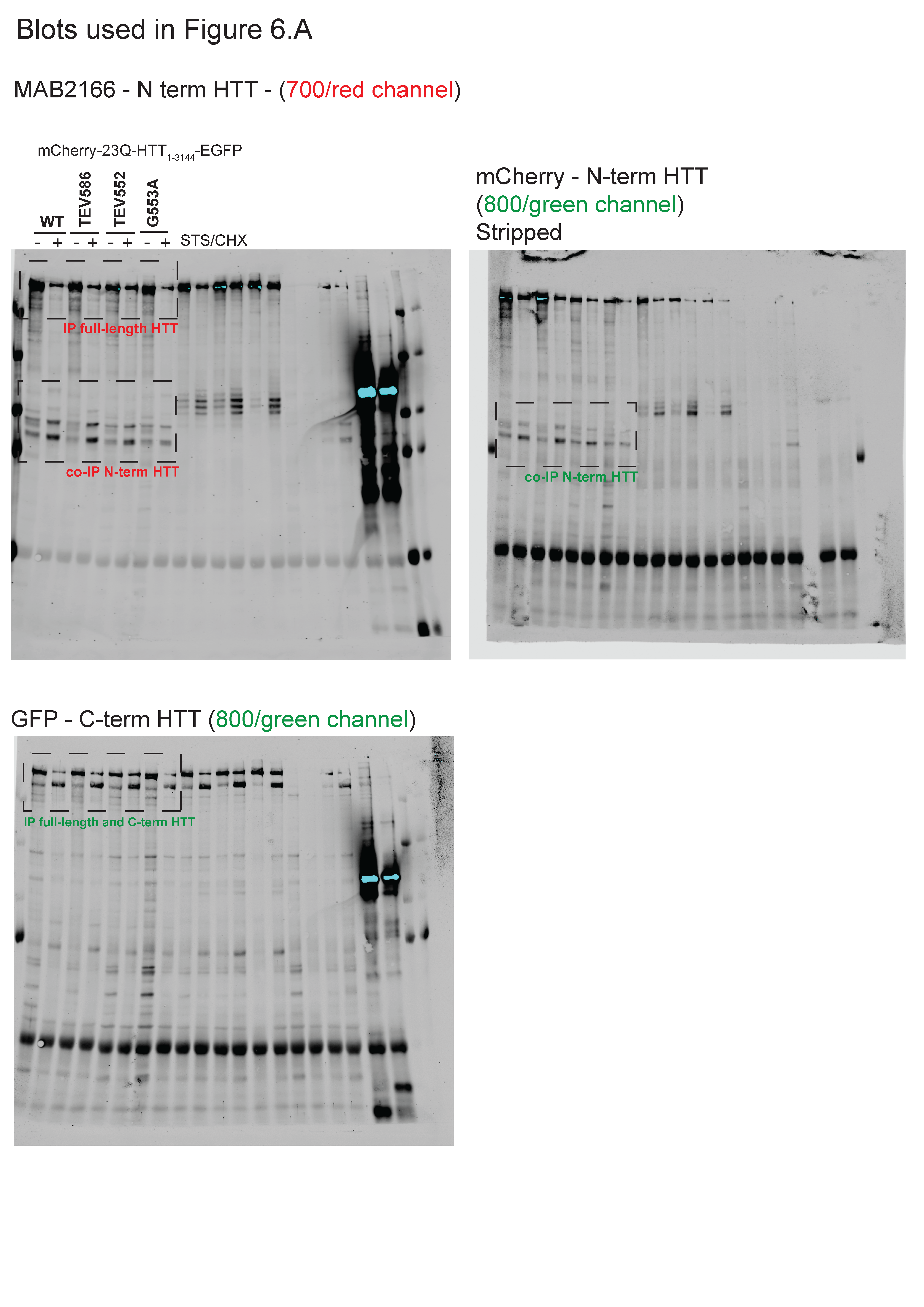

Supplement: Supplementary file 7 [file Image10.TIF]

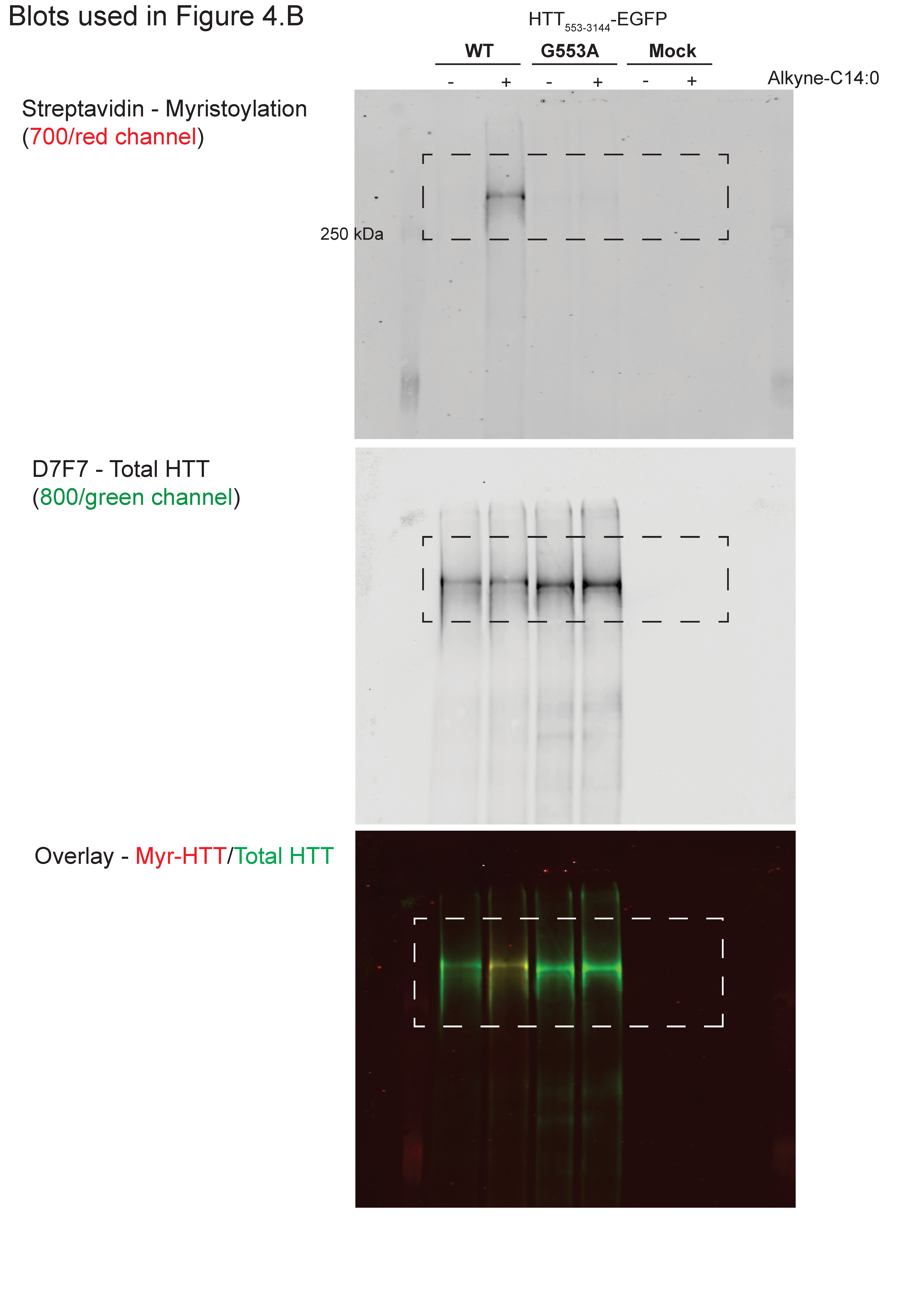

Supplement: Supplementary file 8 [file Image7.TIF]

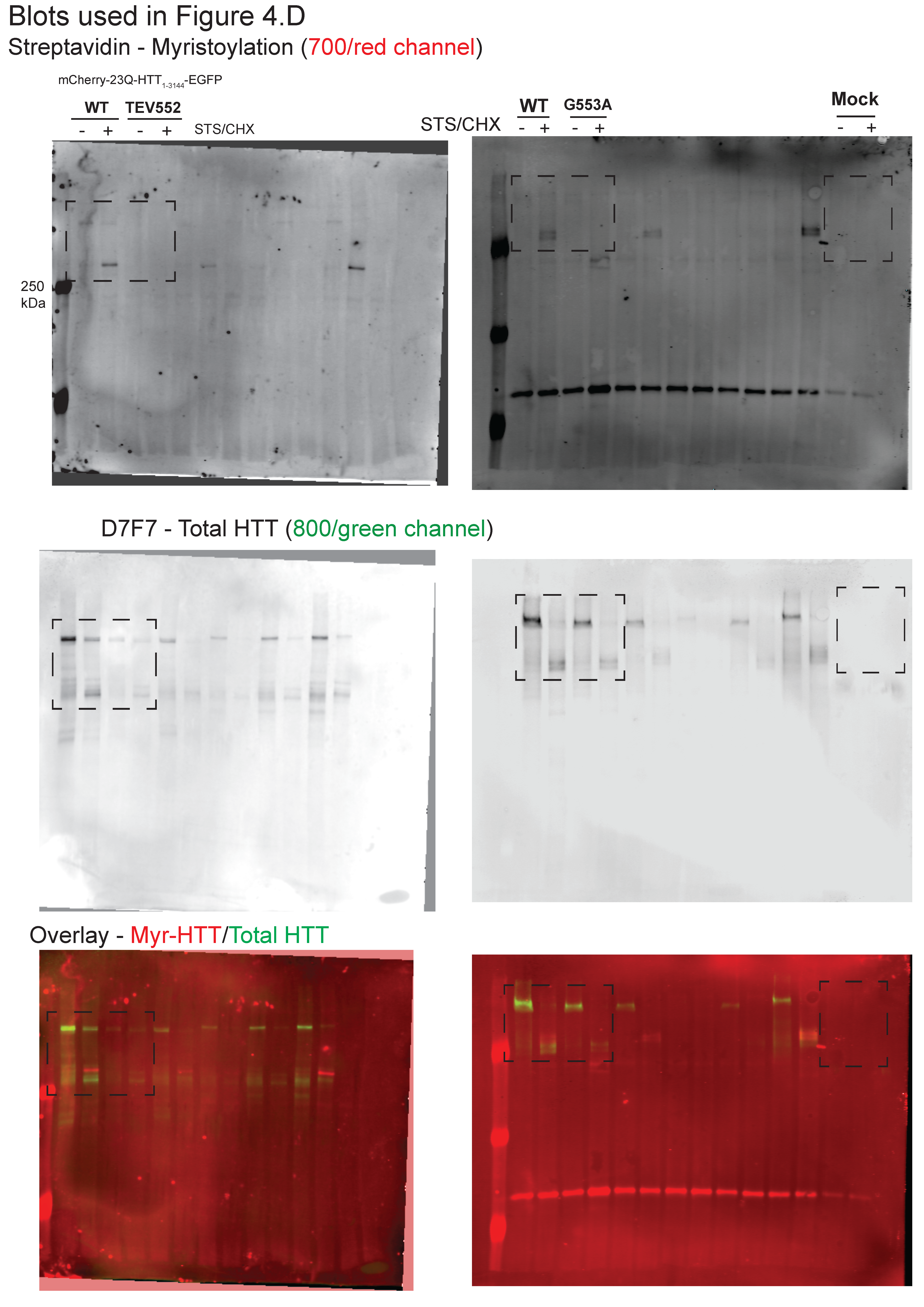

Supplement: Supplementary file 10 [file Image8.TIF]

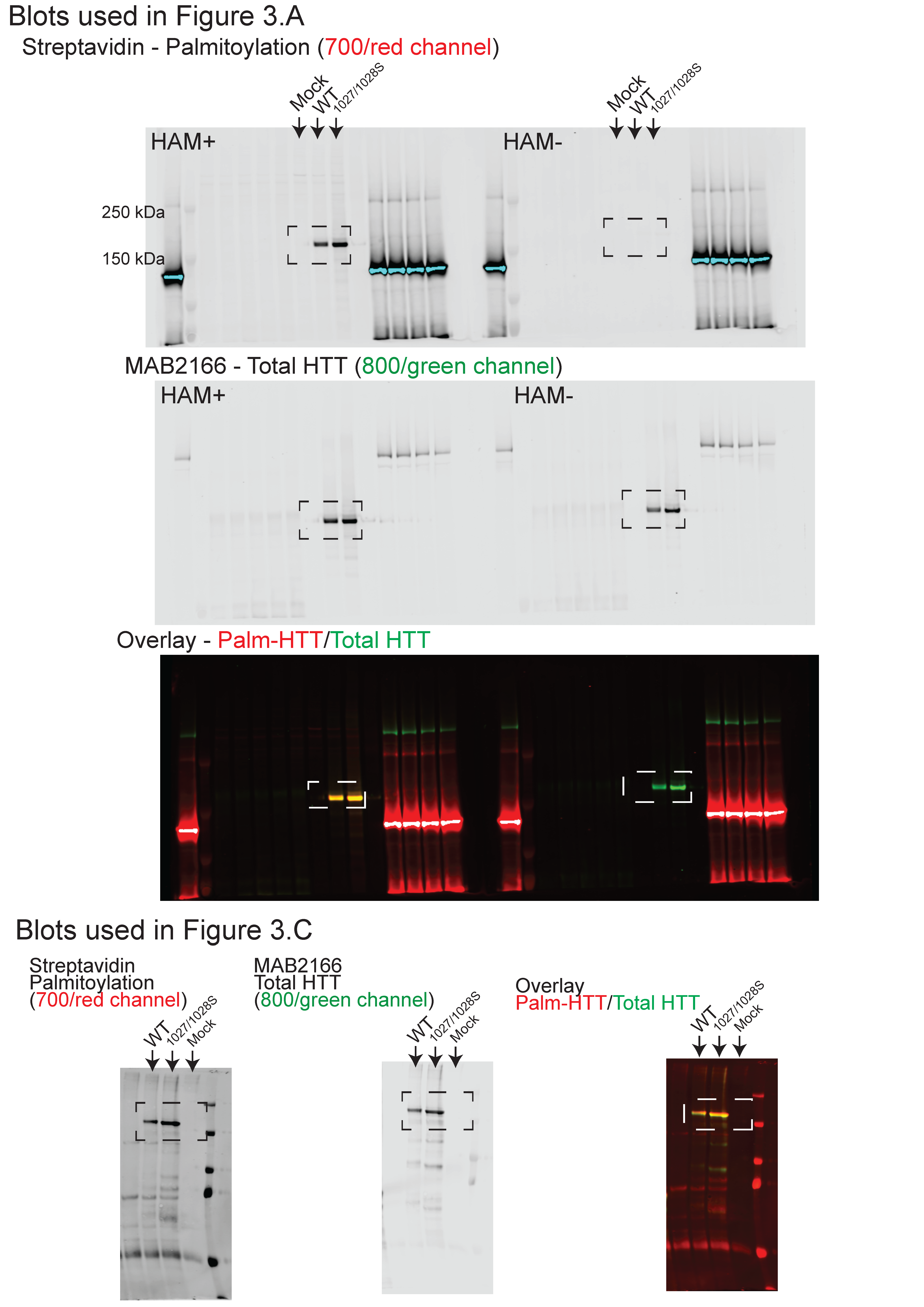

Supplement: Supplementary file 11 [file Image5.TIF]
