# Supplementary material for: Full-length huntingtin is palmitoylated at multiple sites and post-translationally myristoylated following caspase-cleavage
Source: Front Physiol. 2023 Jan 13;14:1086112. doi: 10.3389/fphys.2023.1086112 (PMC9880554; doi:10.3389/fphys.2023.1086112)

A CSS-Palm 3.0 palmitoylation site prediction of full-length Huntingtin

Position	Peptide	Score
**3144**	NVHKVTTC*******	3.2
**1028**	RALTFGCCEALCLLS	2.1
**214**	YLVNLLPCLTRTSKR	1.9
**3134**	PYHRLLTCLRNVHKV	1.5
**1027**	TRALTFGCCEALCLL	1.4
**433**	IAGGGSSCSPVLSRK	1.2
**105**	KKDRVNHCLTICENI	1.1
2362	PKYITAACEMVAEMV	1.1
825	KDESSVTCKLACTAV	1.1
1032	FGCCEALCLLSTAFP	1.1
1313	EPMMATVCVQQLLKT	1.1
840	RNCVMSLCSSSYSEL	1.1
2307	TEFVTHACSLIYCVH	1
2283	DLQAGLDCCCLALQL	0.6
1597	FILVLQQCHKENEDK	0.4
1154	LLKVINICAHVLDDV	0.3
2150	NLSLLAPCLSLGMSE	0.3
638	LLKNMSHCRQPSDSS	0.3
2015	RMVDILACRRVEMLL	0.3
280	AGSAVSICQHSRRTQ	0.3
1710	FSPYLISCTVINRLR	0.3
2312	HACSLIYCVHFILEA	0.2
829	SVTCKLACTAVRNCV	0.2
517	DSVDLASCDLTSSAT	0.2
1302	ILGYLKSCFSREPMM	0.2
944	VPKLFYKCDQGQADP	0.2
2528	AGNPAVSCLEQQPRN	0.2

B Potential new palmitoylation sites of huntingtin

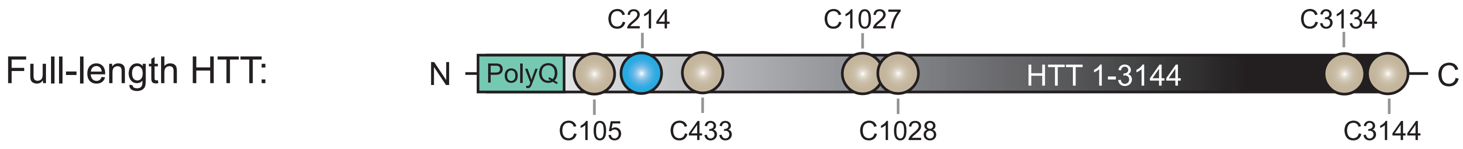

C Cysteine to serine mutant constructs

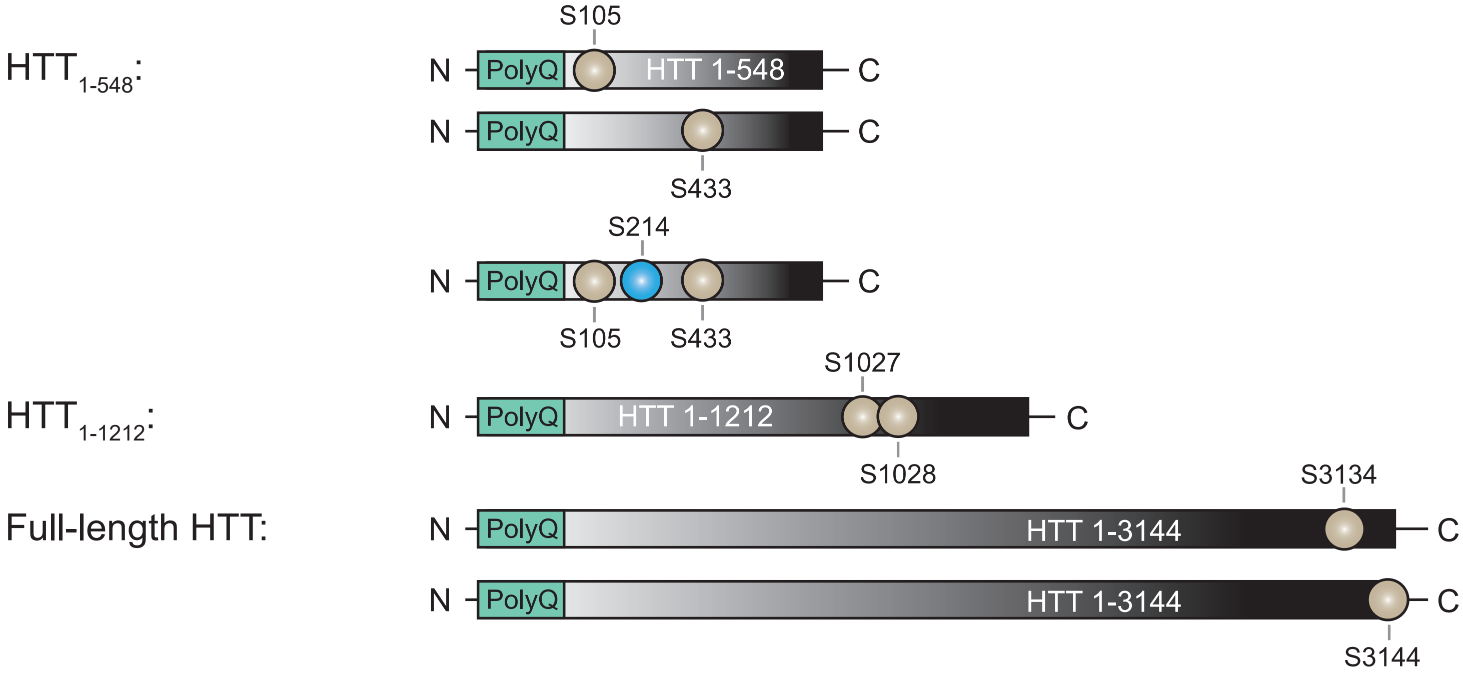

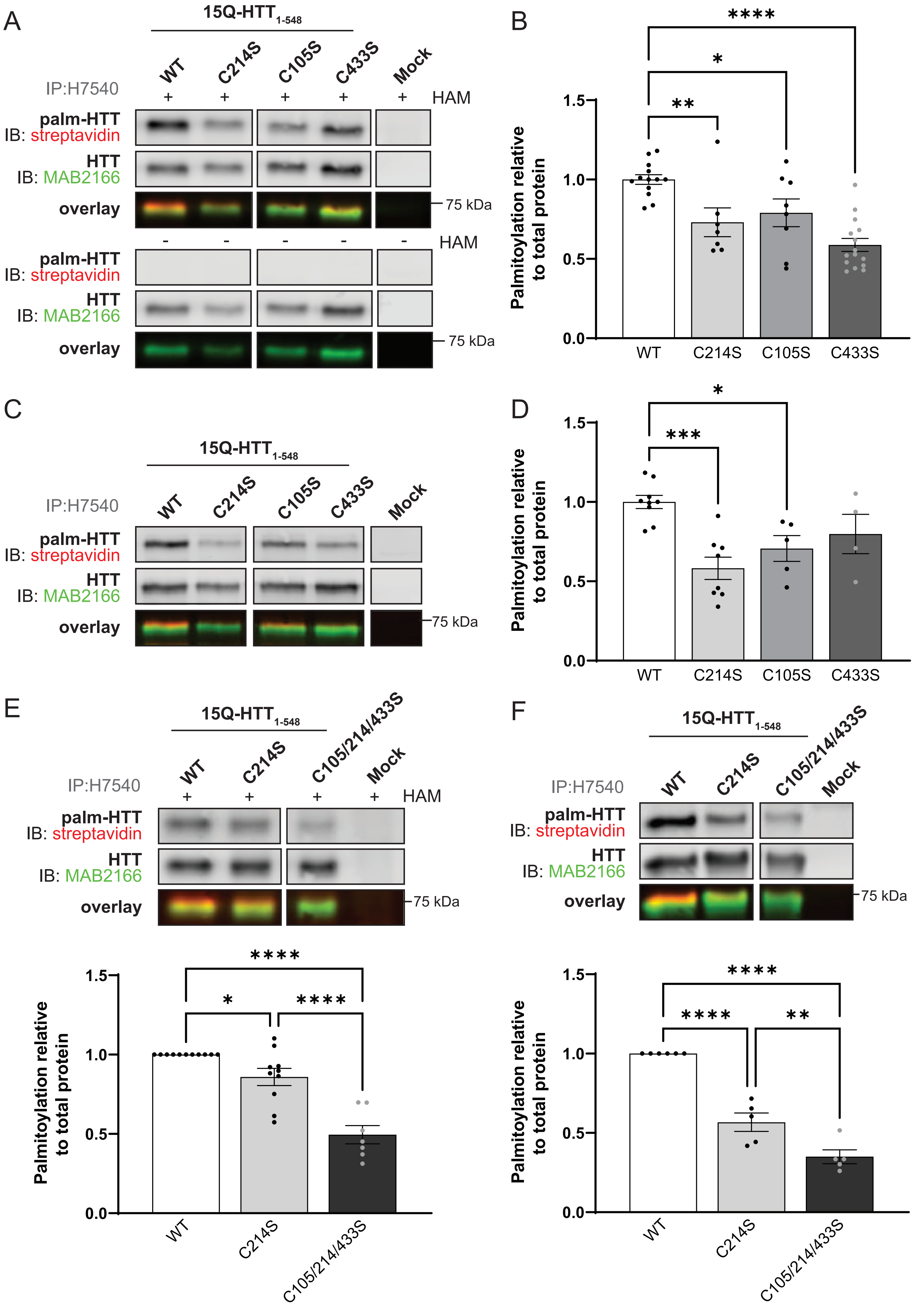

A
15Q-HTT$_{1-548}$
WT
C214S
C105S
C433S
Mock
IP:H7540
+ + + + + HAM
palm-HTT
IB: streptavidin
HTT
IB: MAB2166
overlay
75 kDa
- - - - - HAM
palm-HTT
IB: streptavidin
HTT
IB: MAB2166
overlay
75 kDa
B
Palmitoylation relative to total protein
0.0
0.5
1.0
1.5
WT
C214S
C105S
C433S
****
*
**
C
15Q-HTT$_{1-548}$
IP:H7540
WT
C214S
C105S
C433S
Mock
palm-HTT
IB: streptavidin
HTT
IB: MAB2166
overlay
75 kDa
D
Palmitoylation relative to total protein
0.0
0.5
1.0
1.5
WT
C214S
C105S
C433S
*
***
E
15Q-HTT$_{1-548}$
IP:H7540
WT
C214S
C105/214/433S
Mock
+ + + + HAM
palm-HTT
IB: streptavidin
HTT
IB: MAB2166
overlay
75 kDa
Palmitoylation relative to total protein
0.0
0.5
1.0
1.5
WT
C214S
C105/214/433S
****
*
****
F
15Q-HTT$_{1-548}$
IP:H7540
WT
C214S
C105/214/433S
Mock
palm-HTT
IB: streptavidin
HTT
IB: MAB2166
overlay
75 kDa
Palmitoylation relative to total protein
0.0
0.5
1.0
1.5
WT
C214S
C105/214/433S
****
****
**

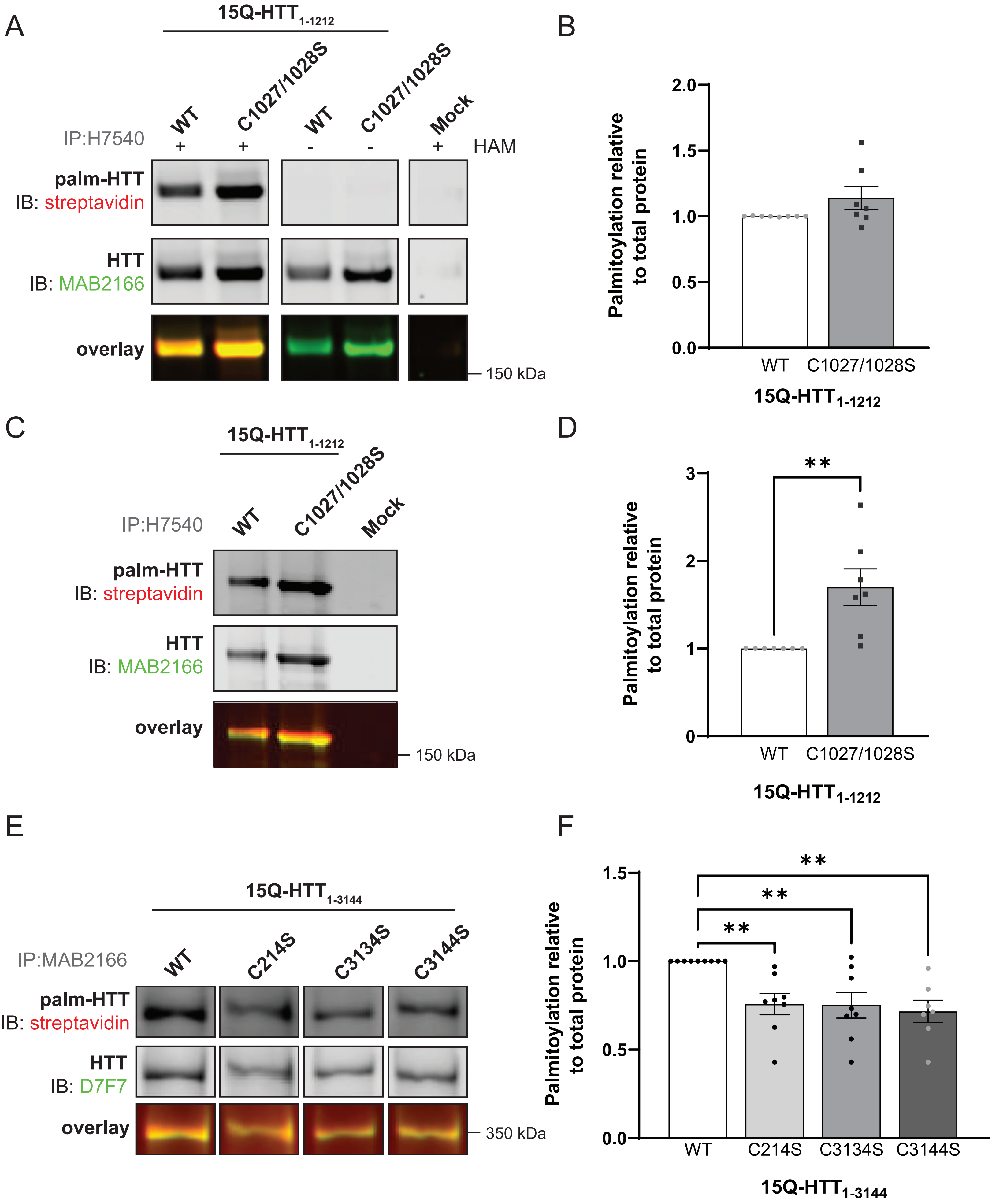
A
15Q-HTT1-1212
WT
C1027/1028S
WT
C1027/1028S
Mock
IP:H7540
+
+
-
-
+
HAM
palm-HTT
IB: streptavidin
HTT
IB: MAB2166
overlay
150 kDa
B
Palmitoylation relative to total protein
2.0
1.5
1.0
0.5
0.0
WT
C1027/1028S
15Q-HTT1-1212
C
15Q-HTT1-1212
IP:H7540
WT
C1027/1028S
Mock
palm-HTT
IB: streptavidin
HTT
IB: MAB2166
overlay
150 kDa
D
**
Palmitoylation relative to total protein
3
2
1
0
WT
C1027/1028S
15Q-HTT1-1212
E
15Q-HTT1-3144
IP:MAB2166
WT
C214S
C3134S
C3144S
palm-HTT
IB: streptavidin
HTT
IB: D7F7
overlay
350 kDa
F
**
**
**
Palmitoylation relative to total protein
1.5
1.0
0.5
0.0
WT
C214S
C3134S
C3144S
15Q-HTT1-3144

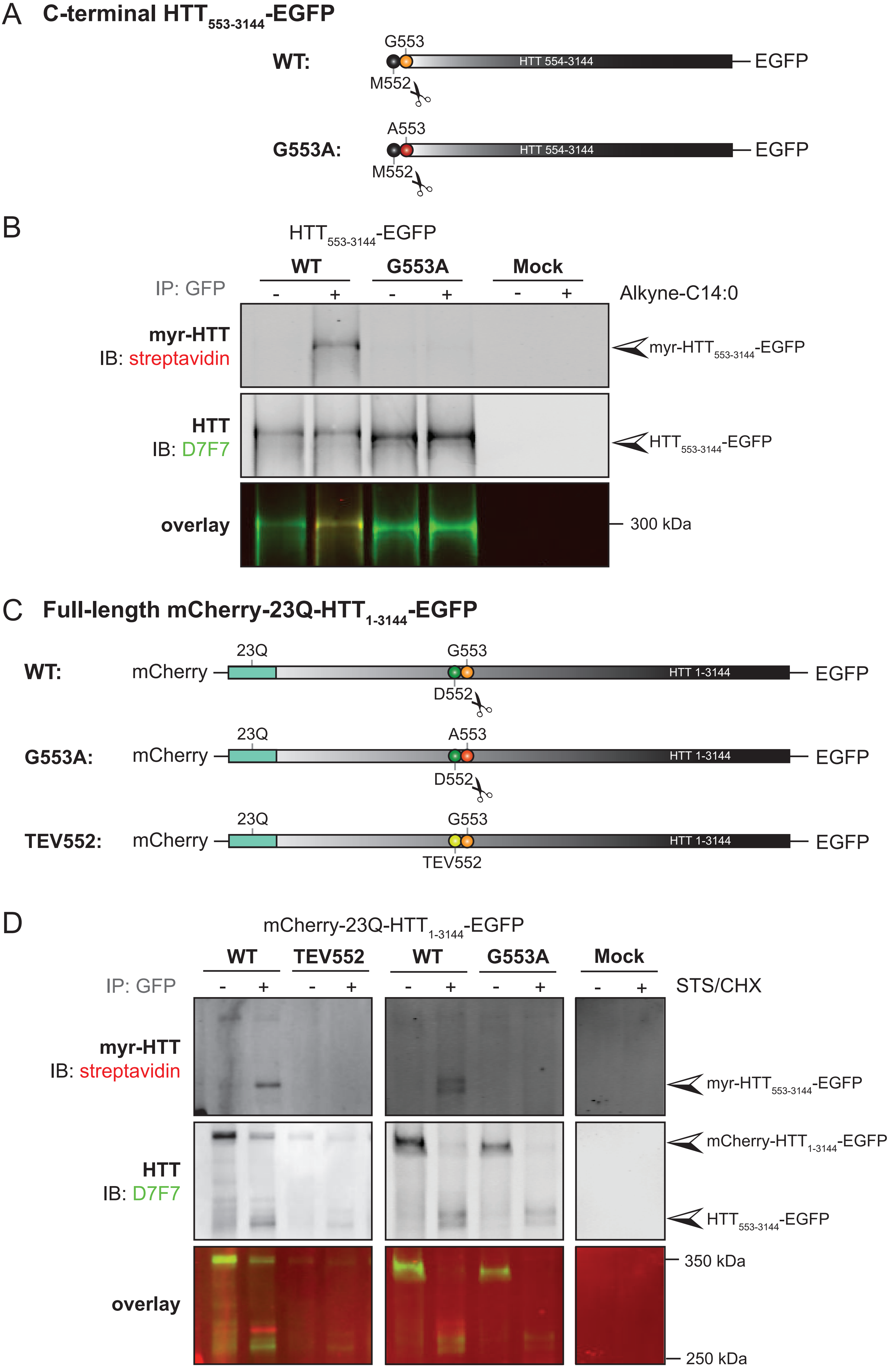
A
C-terminal HTT553-3144-EGFP
WT:
G553
HTT 554-3144
EGFP
M552
G553A:
A553
HTT 554-3144
EGFP
M552
B
HTT553-3144-EGFP
WT
G553A
Mock
IP: GFP
- + - + - +
Alkyne-C14:0
myr-HTT
IB: streptavidin
myr-HTT553-3144-EGFP
HTT
IB: D7F7
HTT553-3144-EGFP
overlay
300 kDa
C
Full-length mCherry-23Q-HTT1-3144-EGFP
WT:
mCherry
23Q
G553
HTT 1-3144
EGFP
D552
G553A:
mCherry
23Q
A553
HTT 1-3144
EGFP
D552
TEV552:
mCherry
23Q
G553
HTT 1-3144
EGFP
TEV552
D
mCherry-23Q-HTT1-3144-EGFP
WT
TEV552
WT
G553A
Mock
IP: GFP
- + - + - + - + - +
STS/CHX
myr-HTT
IB: streptavidin
myr-HTT553-3144-EGFP
HTT
IB: D7F7
mCherry-HTT1-3144-EGFP
HTT553-3144-EGFP
overlay
350 kDa
250 kDa

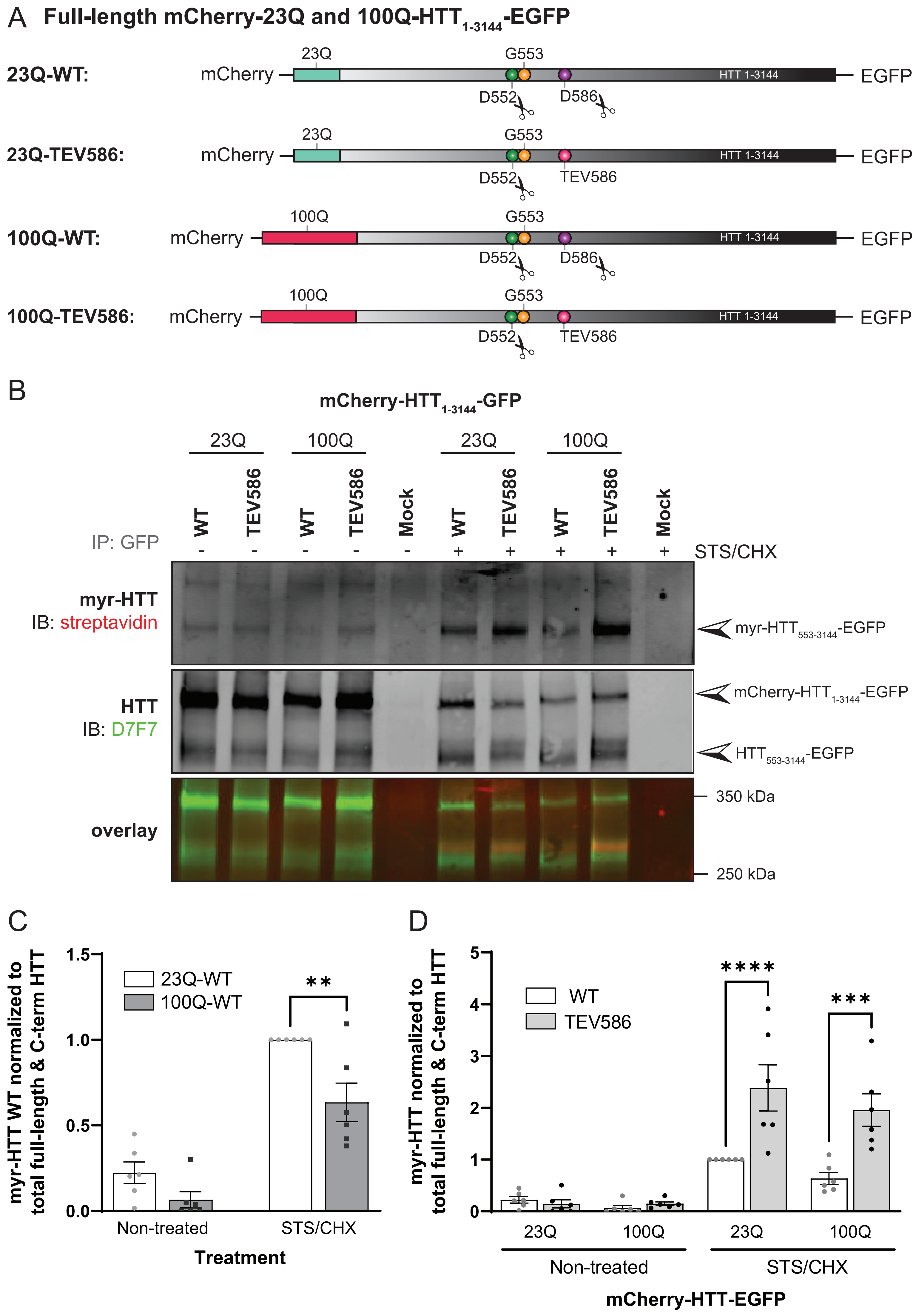

A Full-length mCherry-23Q and 100Q-HTT1-3144-EGFP
23Q-WT:
23Q-TEV586:
100Q-WT:
100Q-TEV586:
mCherry
EGFP
23Q
100Q
G553
D552
D586
TEV586
HTT 1-3144
B
mCherry-HTT1-3144-GFP
23Q
100Q
WT
TEV586
Mock
IP: GFP
STS/CHX
myr-HTT
IB: streptavidin
HTT
IB: D7F7
overlay
myr-HTT553-3144-EGFP
mCherry-HTT1-3144-EGFP
HTT553-3144-EGFP
350 kDa
250 kDa
C
myr-HTT WT normalized to total full-length & C-term HTT
23Q-WT
100Q-WT
**
Non-treated
STS/CHX
Treatment
D
myr-HTT normalized to total full-length & C-term HTT
WT
TEV586
****
***
23Q
100Q
Non-treated
STS/CHX
mCherry-HTT-EGFP

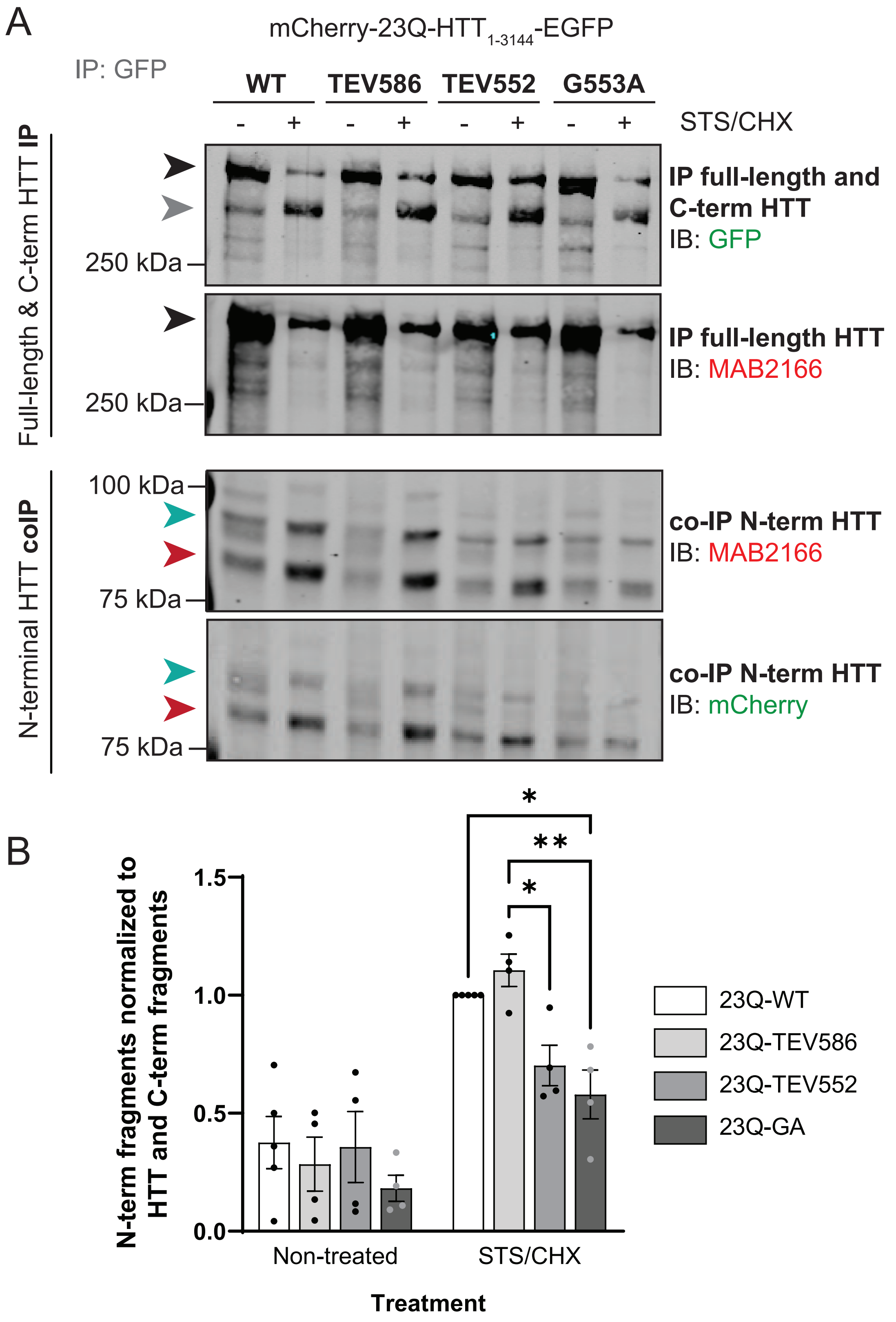

A
mCherry-23Q-HTT$_{1-3144}$-EGFP
IP: GFP
WT
TEV586
TEV552
G553A
STS/CHX
Full-length & C-term HTT IP
250 kDa
IP full-length and C-term HTT
IB: GFP
IP full-length HTT
IB: MAB2166
N-terminal HTT coIP
100 kDa
75 kDa
co-IP N-term HTT
IB: MAB2166
co-IP N-term HTT
IB: mCherry
B
N-term fragments normalized to HTT and C-term fragments
1.5
1.0
0.5
0.0
Non-treated
STS/CHX
Treatment
23Q-WT
23Q-TEV586
23Q-TEV552
23Q-GA

Blots used in Figure 2A

Streptavidin - Palmitoylation (700/red channel)

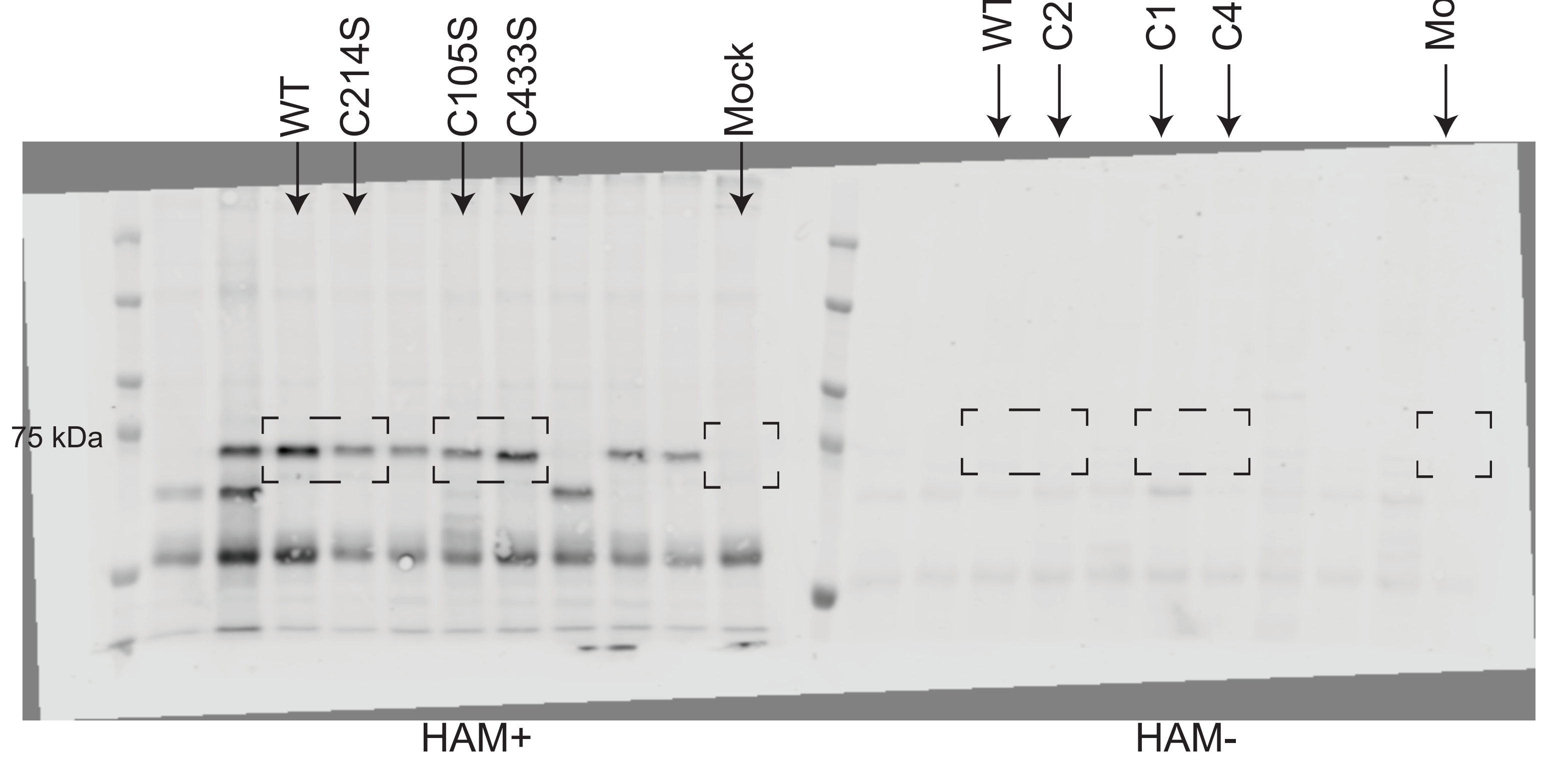

MAB2166 - Total HTT (800/green channel)

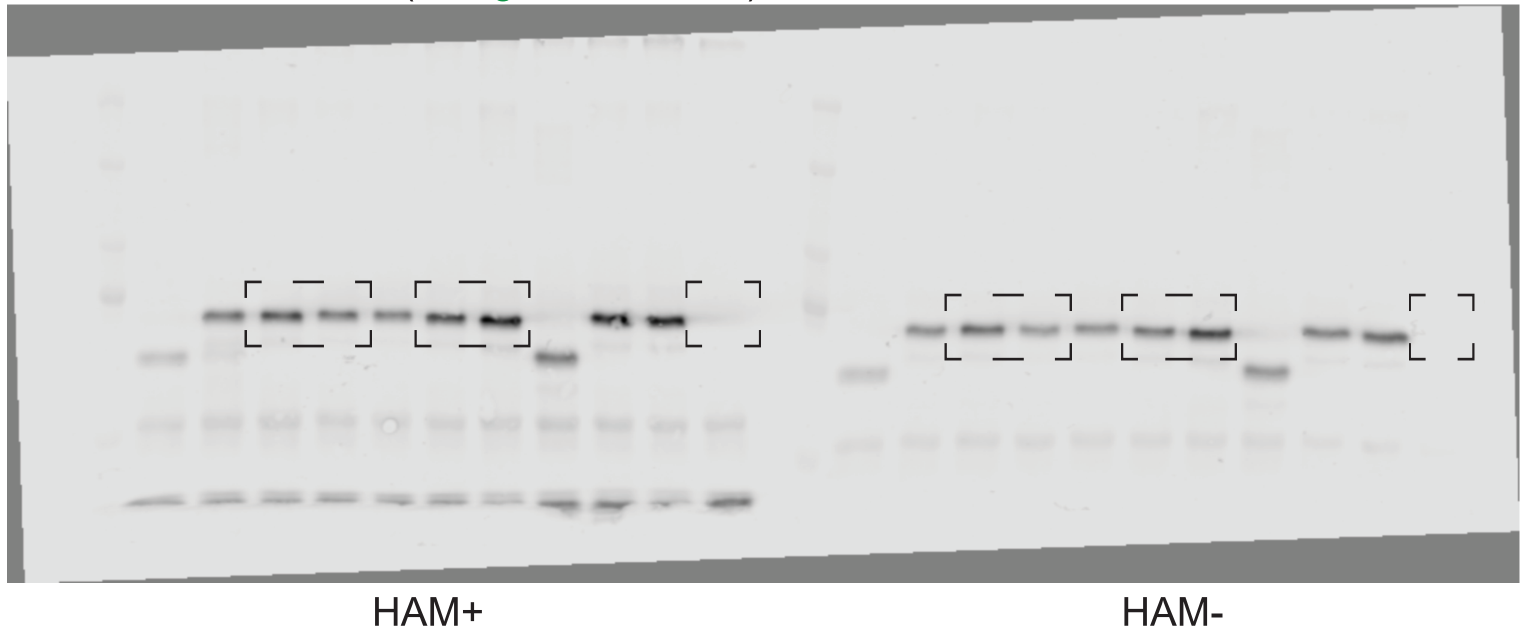

Overlay - Palm-HTT/Total HTT

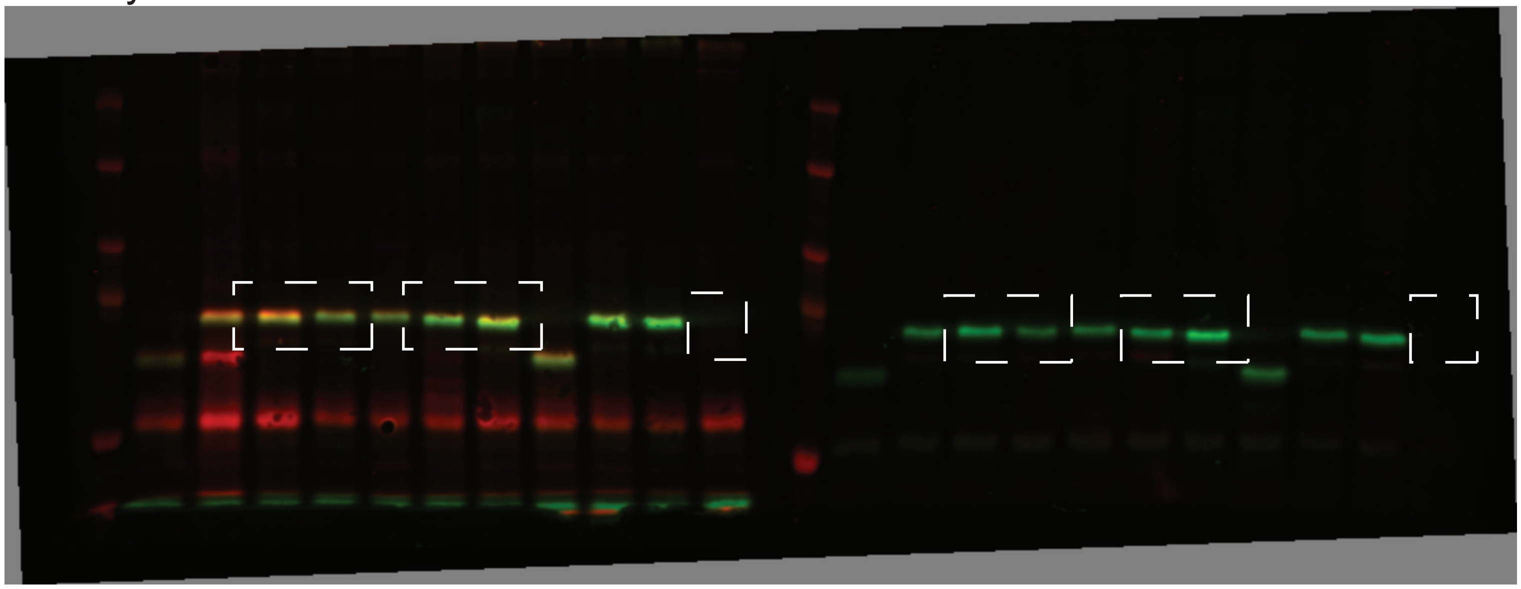

Blots used in Figure 2.C

Streptavidin - Palmitoylation (700/red channel)

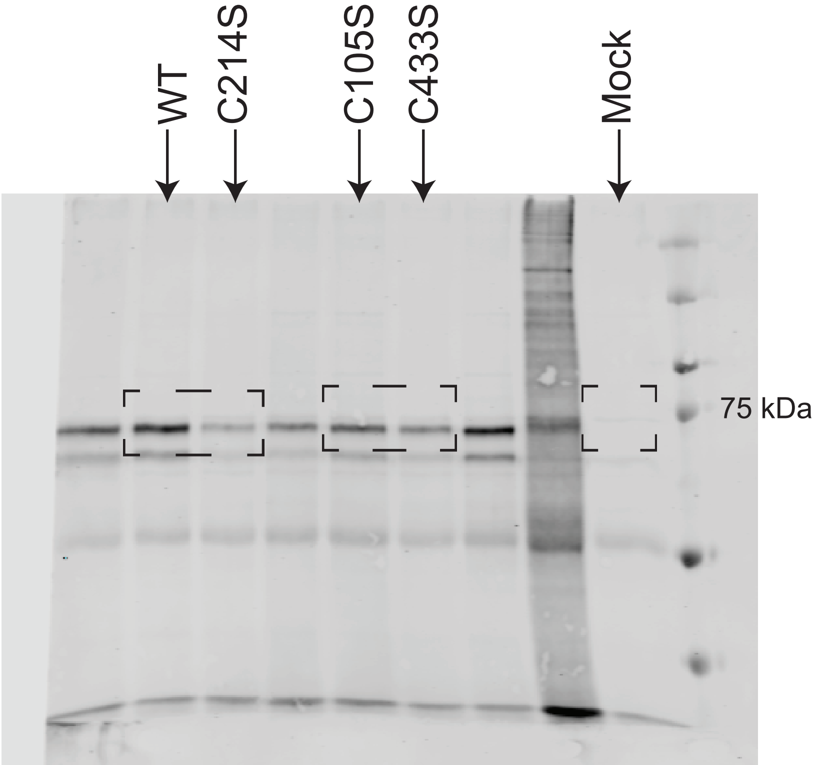

MAB2166 - Total HTT (800/green channel)

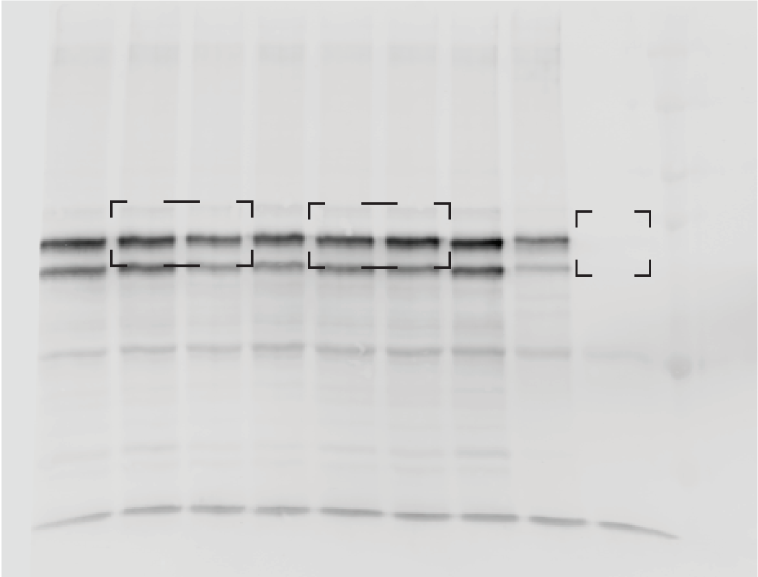

Overlay - Palm-HTT/Total HTT

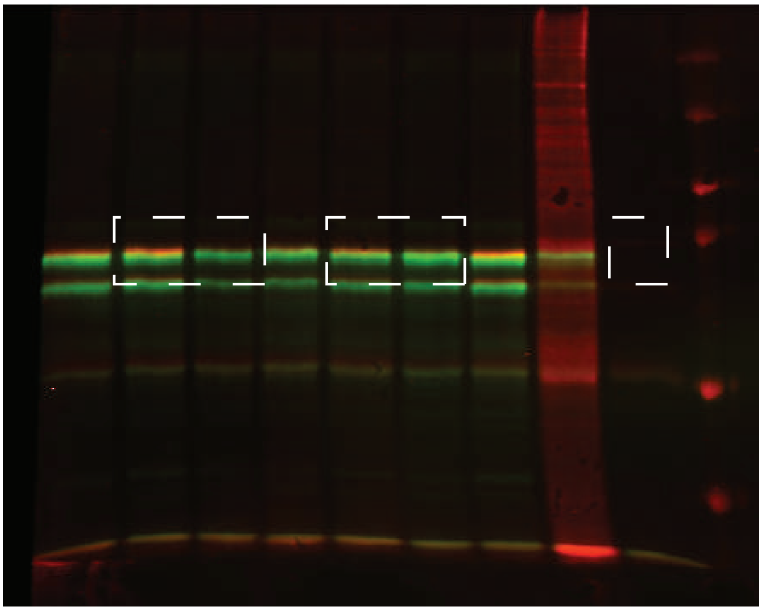

Blots used in Figure 2.E

Streptavidin - Palmitoylation (700/red channel)

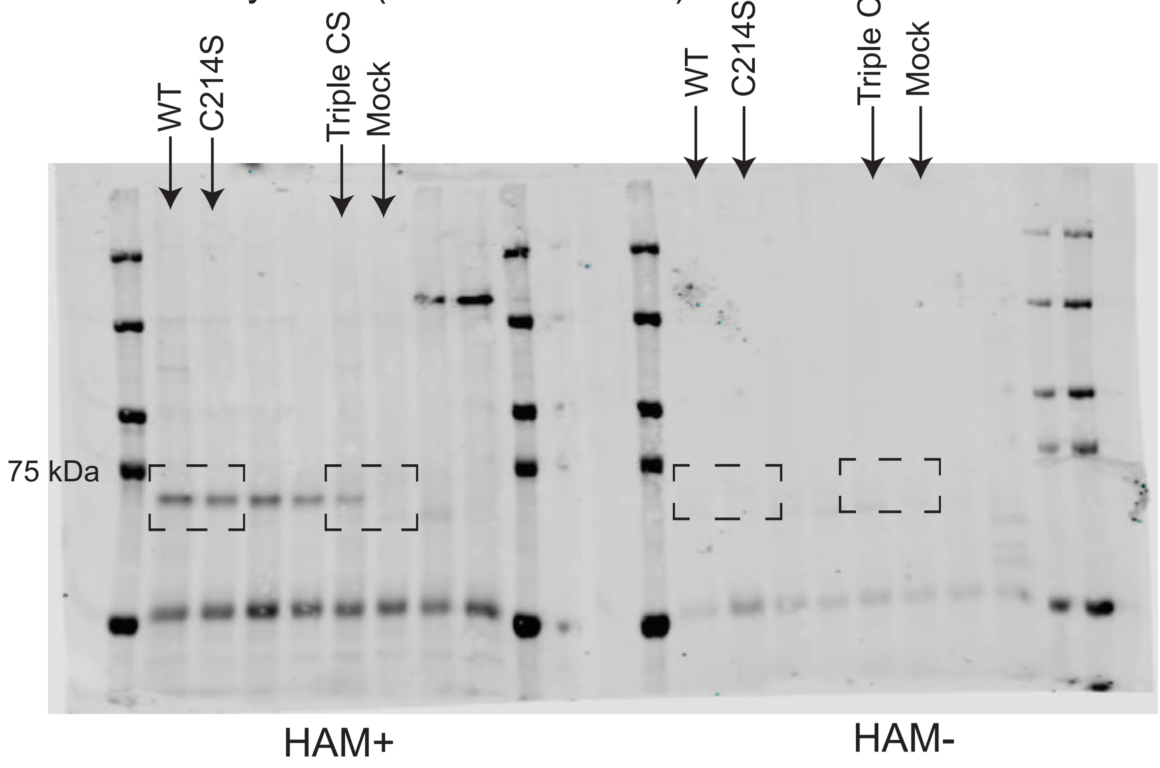

MAB2166 - Total HTT (800/green channel)

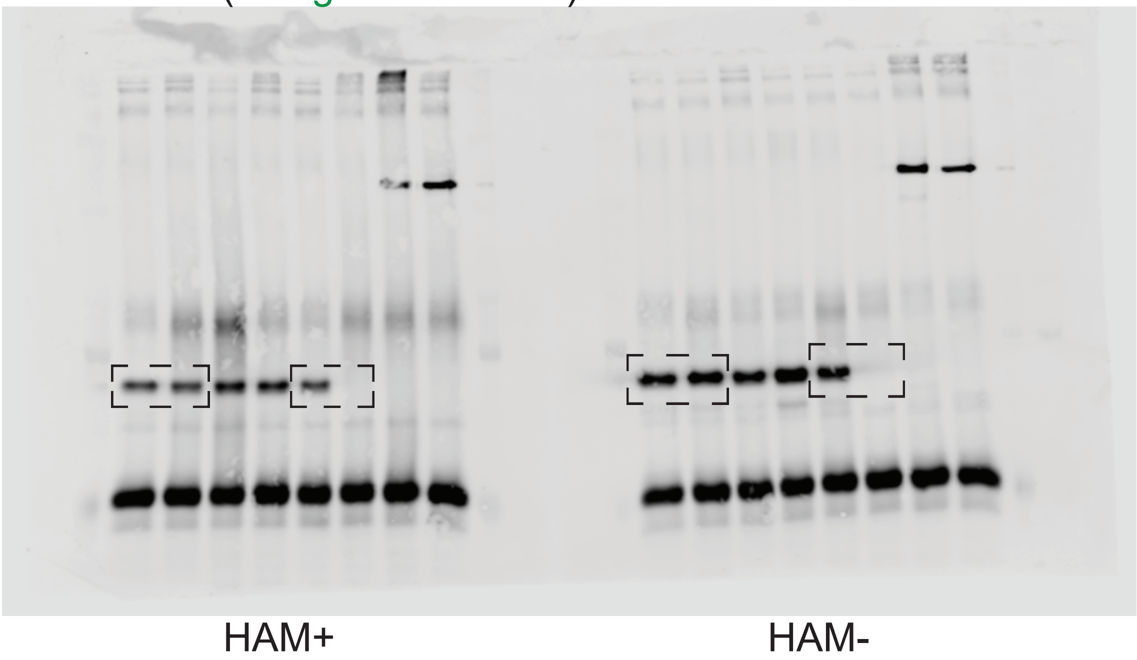

Overlay - Palm-HTT/Total HTT

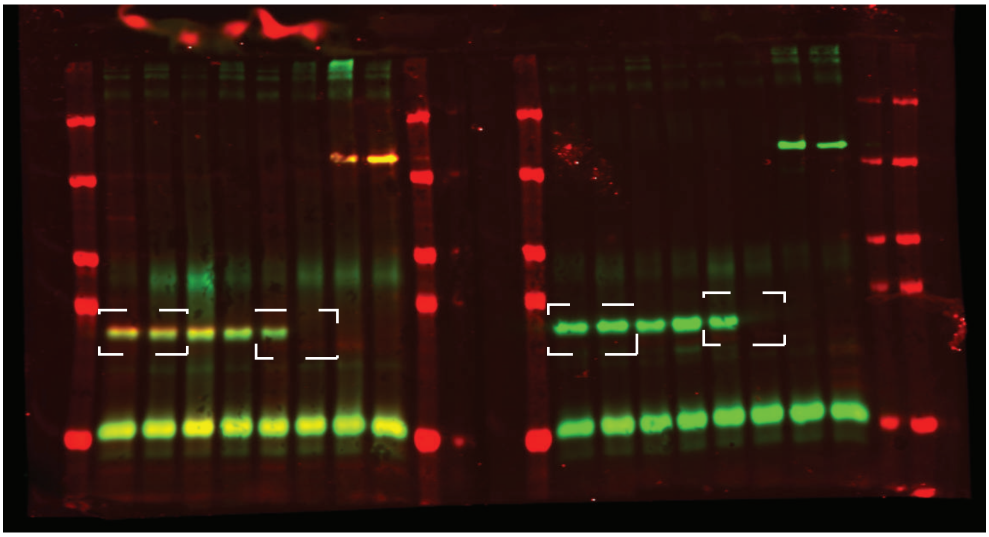

Blots used in Figure 2.F

Streptavidin - Palmitoylation (700/red channel)

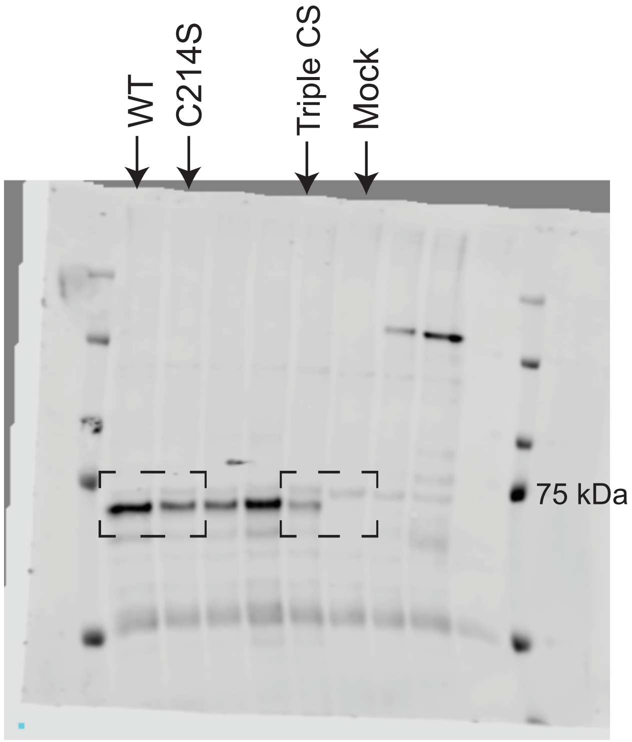

MAB2166 - Total HTT (800/green channel)

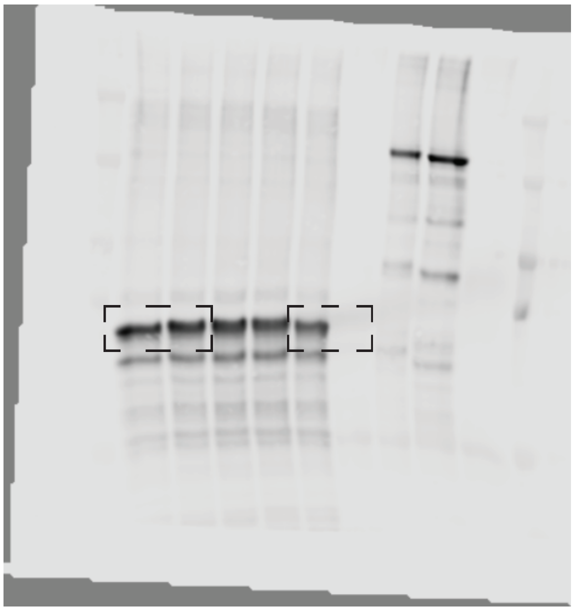

Overlay - Palm-HTT/Total HTT

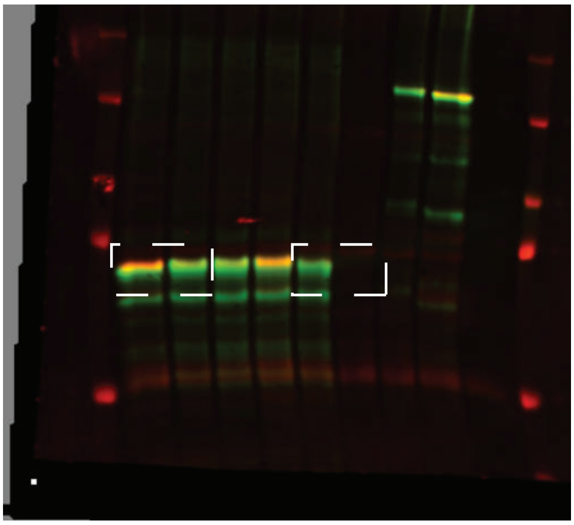

Blots used in Figure 3.A

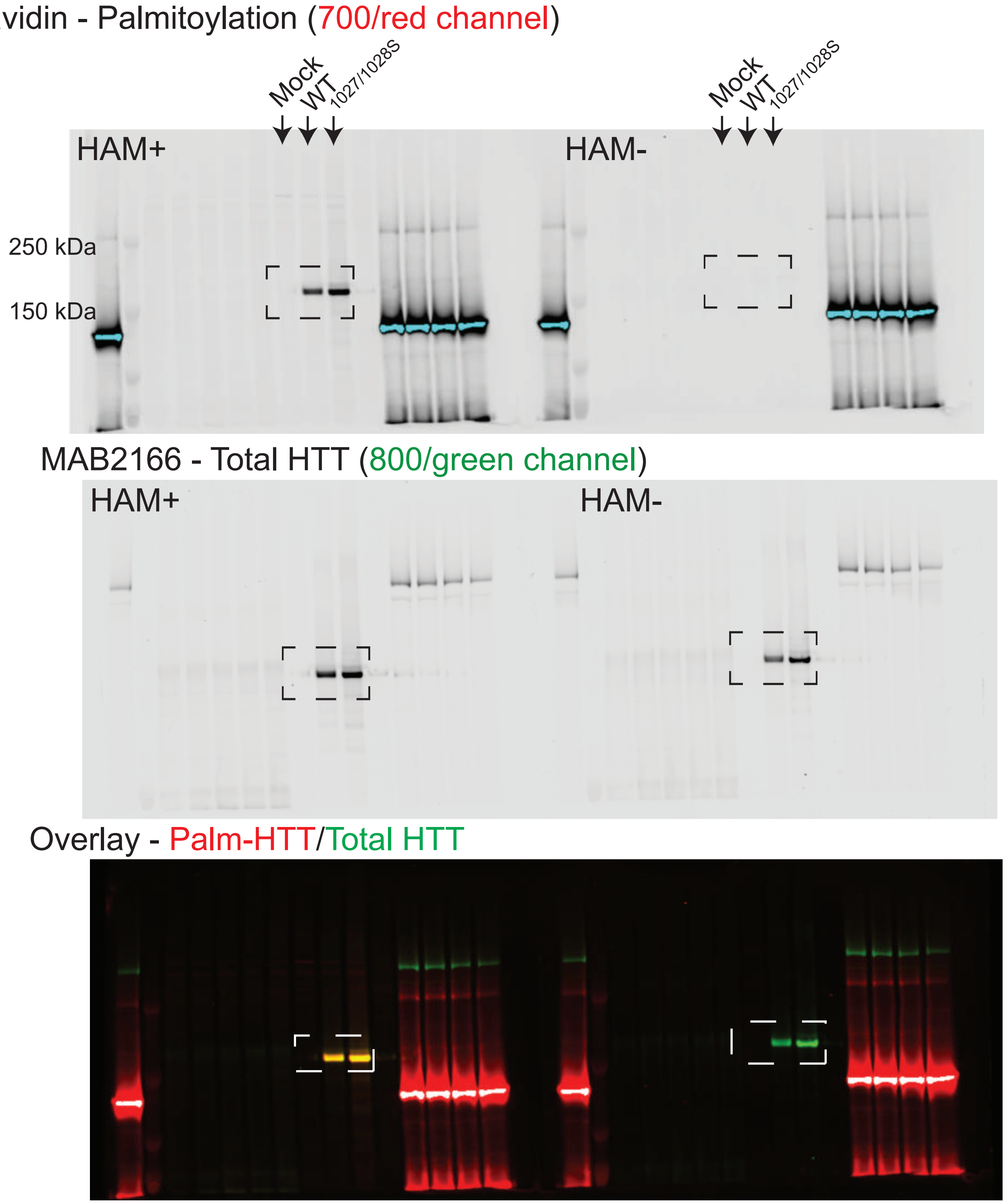

Blots used in Figure 3.C

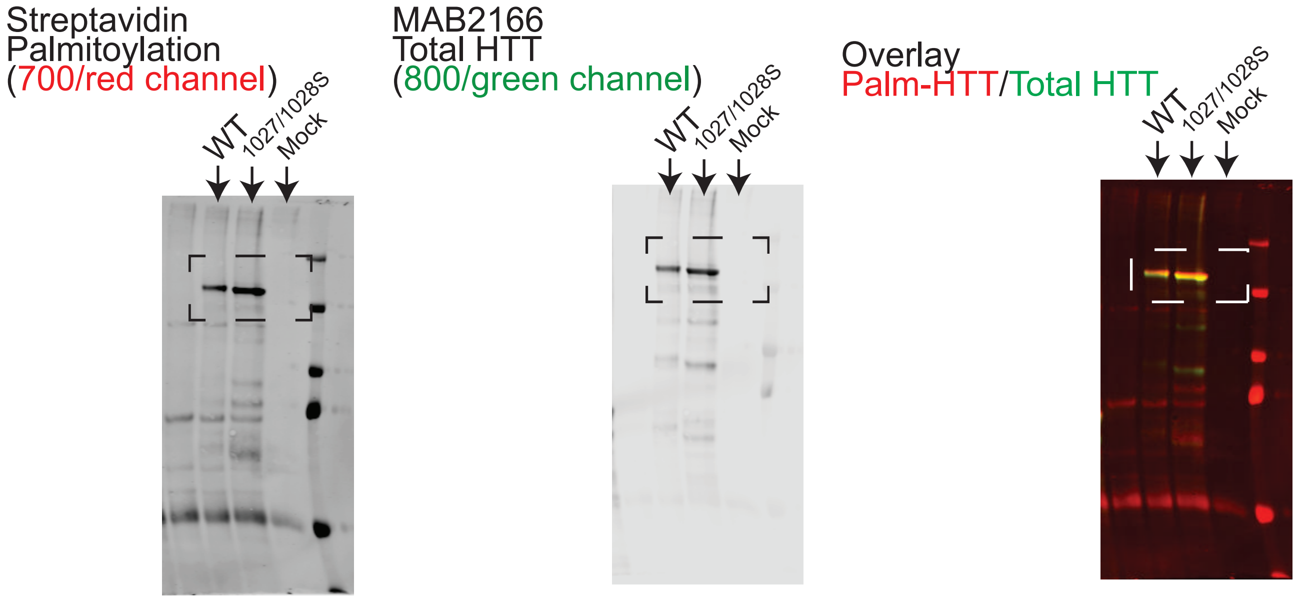

Blots used in Figure 3.E

Streptavidin - Palm
(700/red channel)

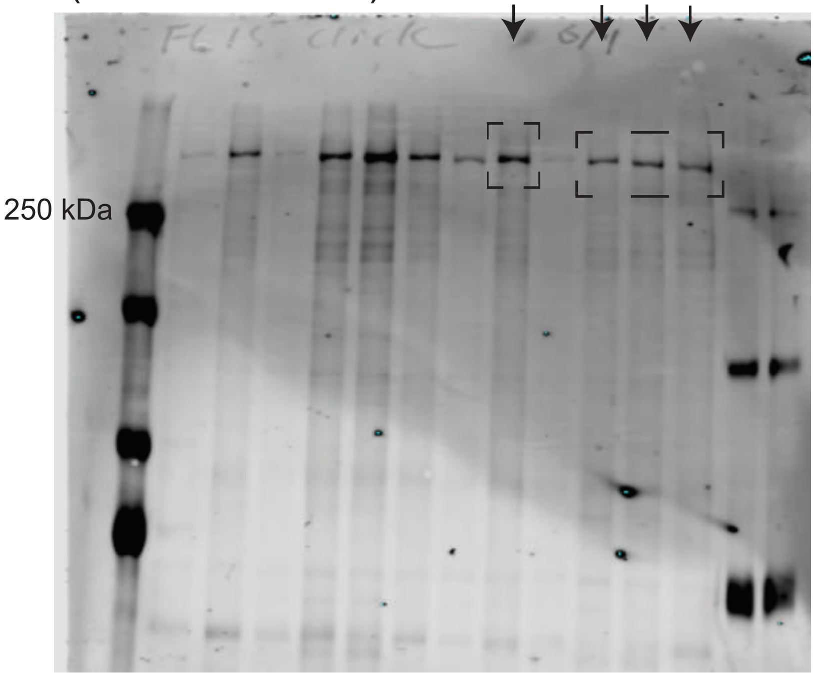

D7F7 - Total HTT (800/green channel)

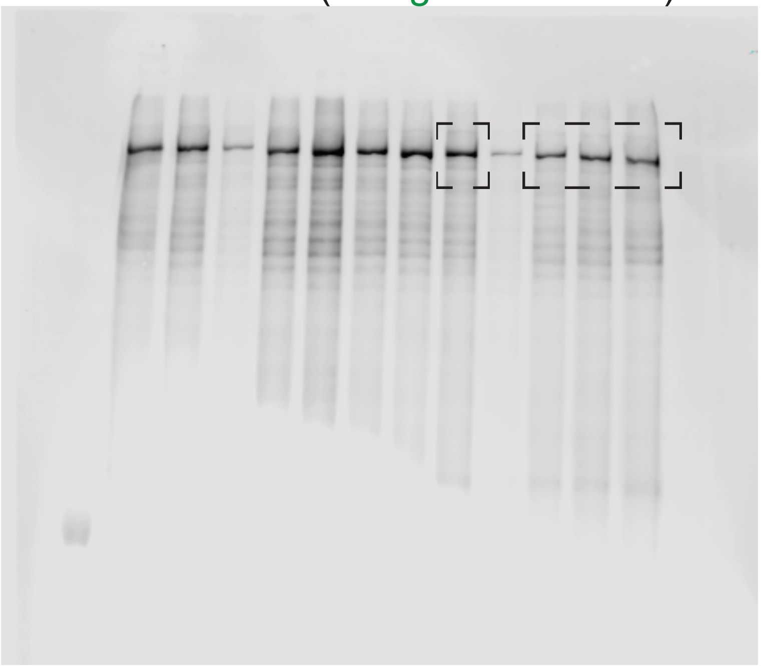

Overlay - Palm-HTT/Total HTT

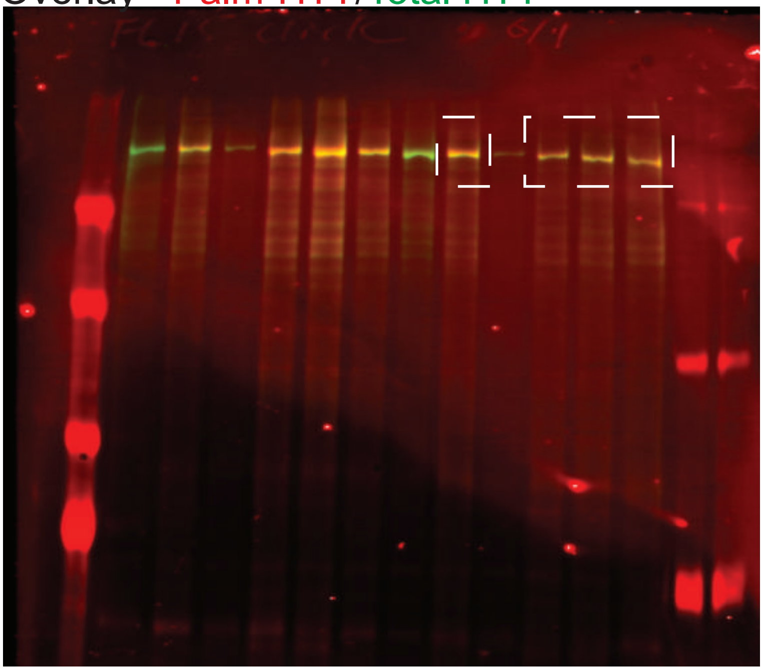

Blots used in Figure 4.B

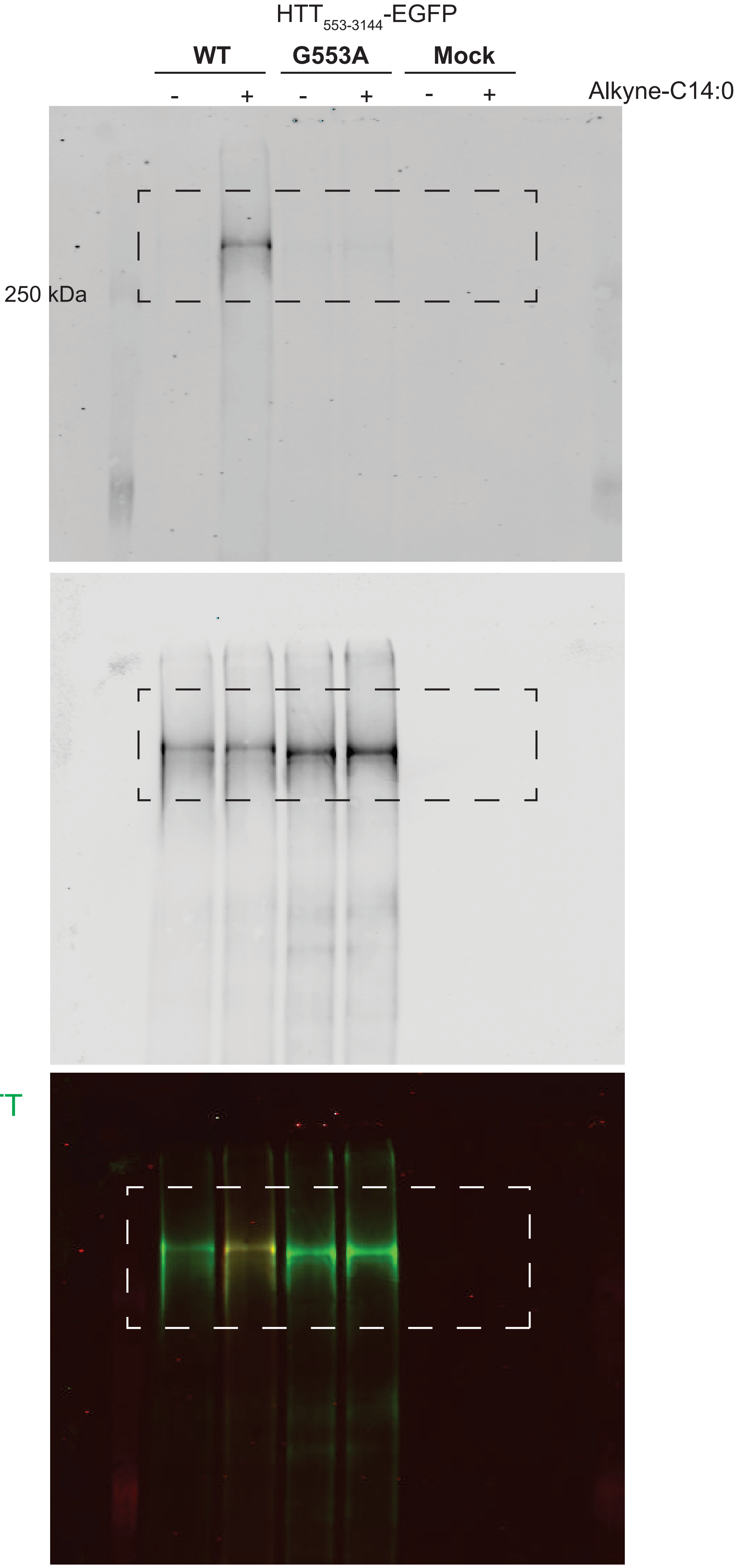

Streptavidin - Myristoylation
(700/red channel)

D7F7 - Total HTT
(800/green channel)

Overlay - Myr-HTT/Total HTT

Blots used in Figure 4.D

Streptavidin - Myristoylation (700/red channel)

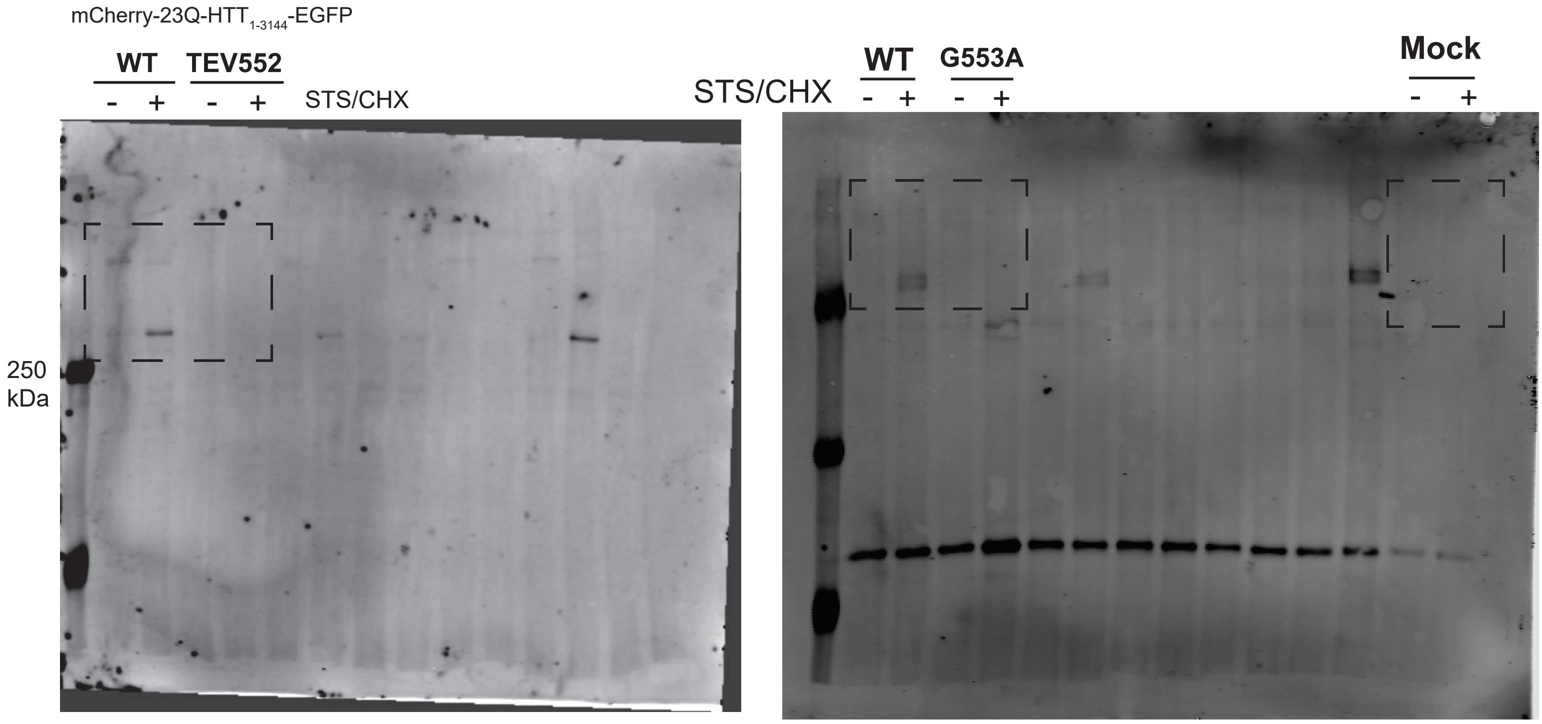

D7F7 - Total HTT (800/green channel)

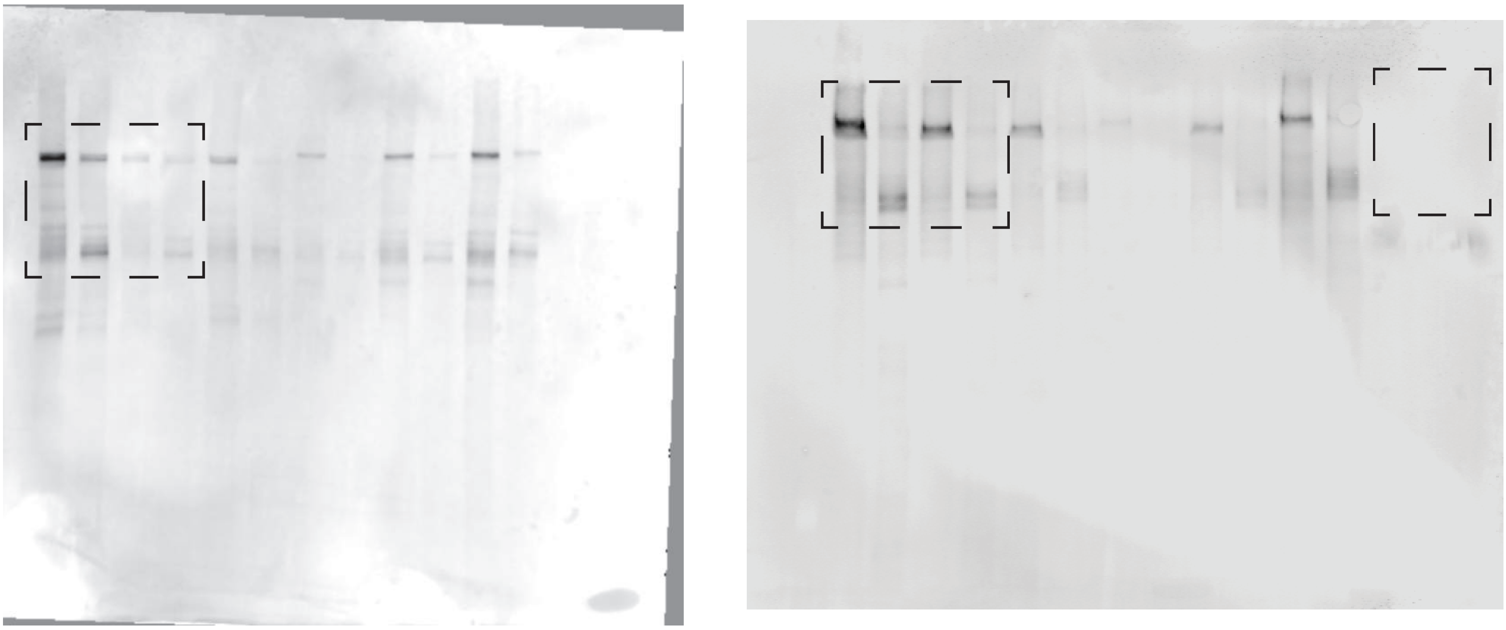

Overlay - Myr-HTT/Total HTT

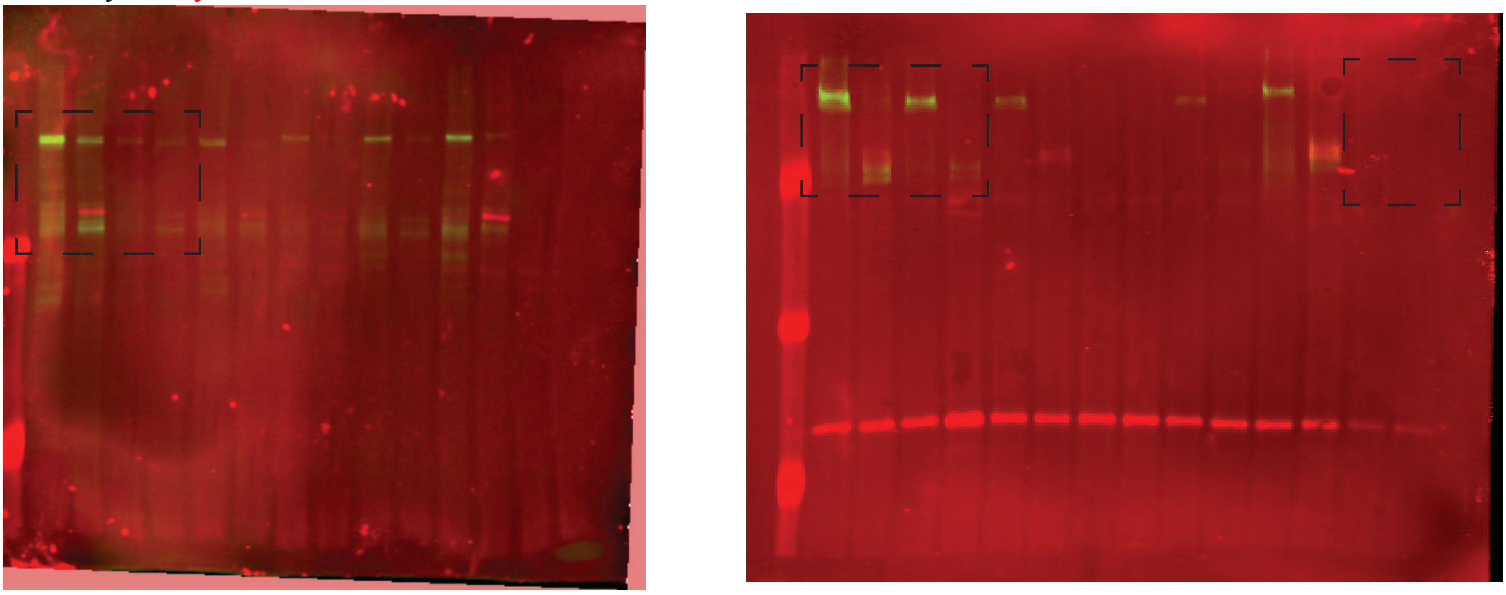

Blots used in Figure 5.B

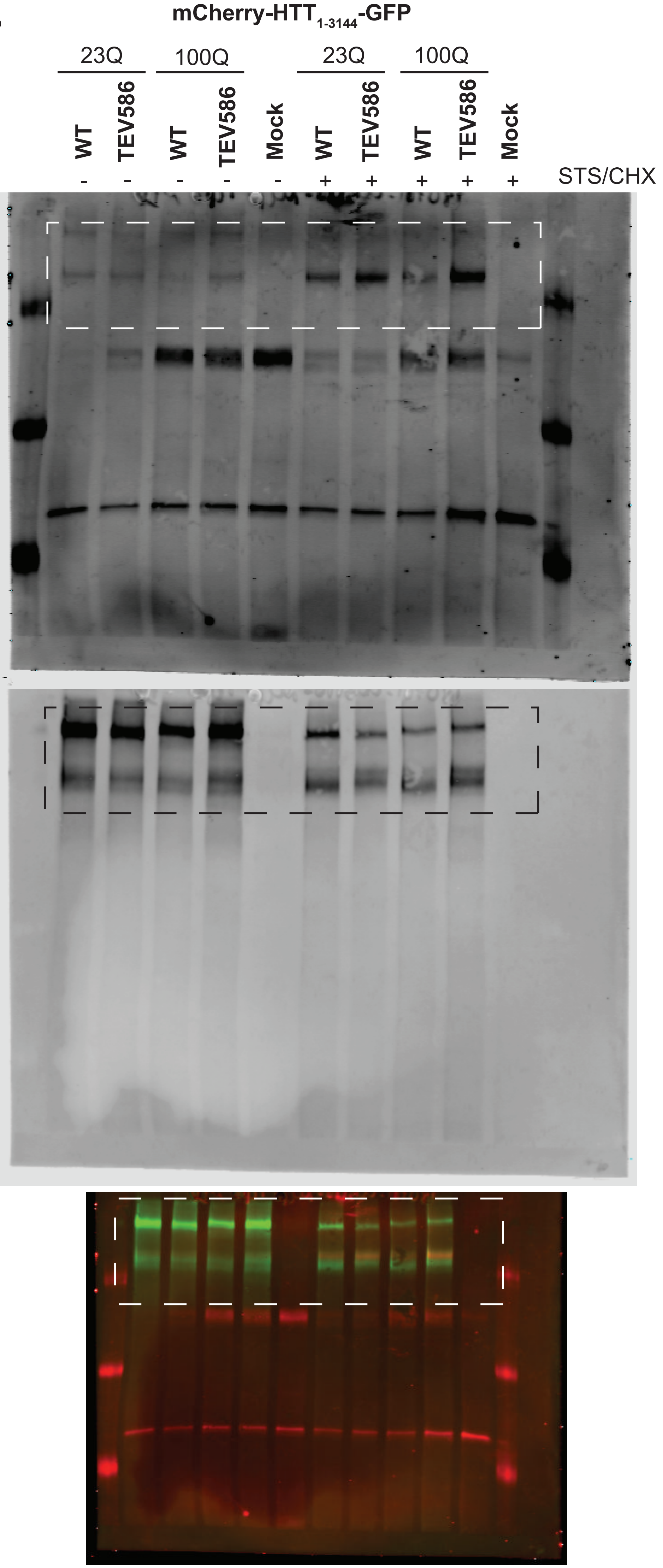

Blots used in Figure 6.A

MAB2166 - N term HTT - (700/red channel)

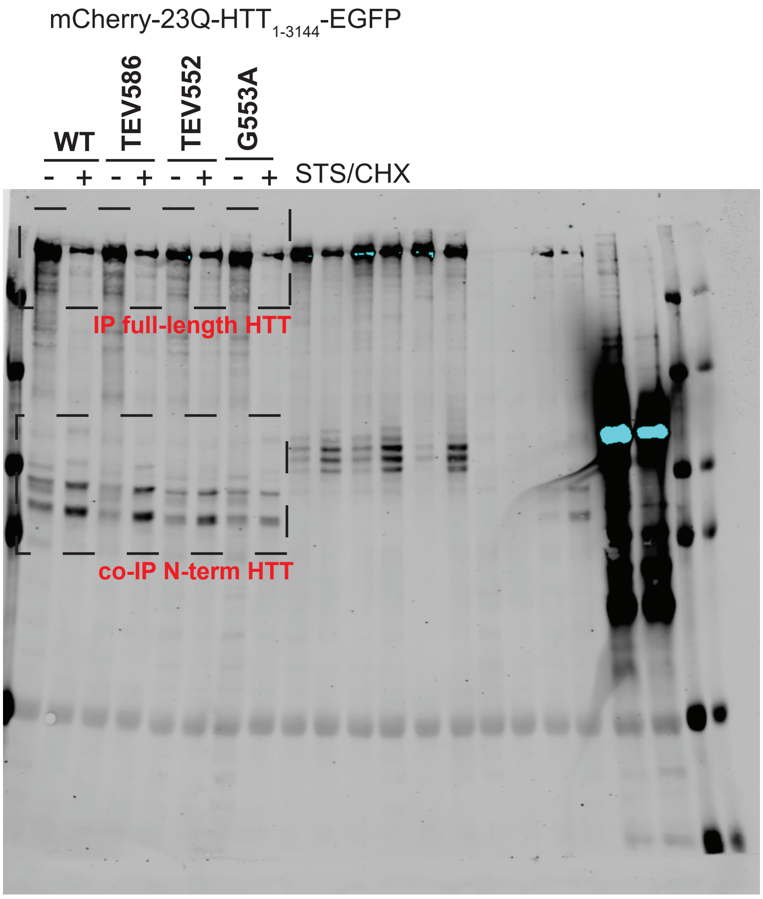

mCherry - N-term HTT (800/green channel) Stripped

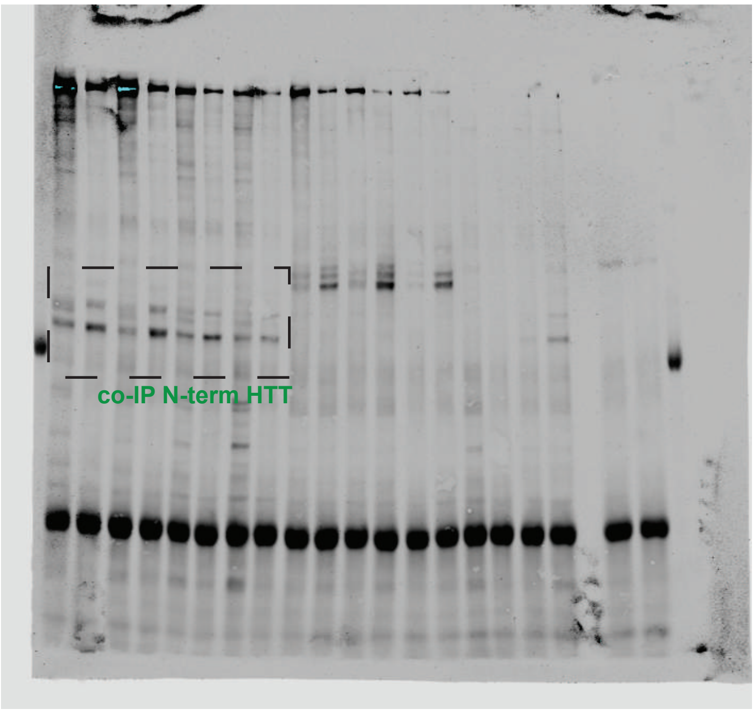

GFP - C-term HTT (800/green channel)

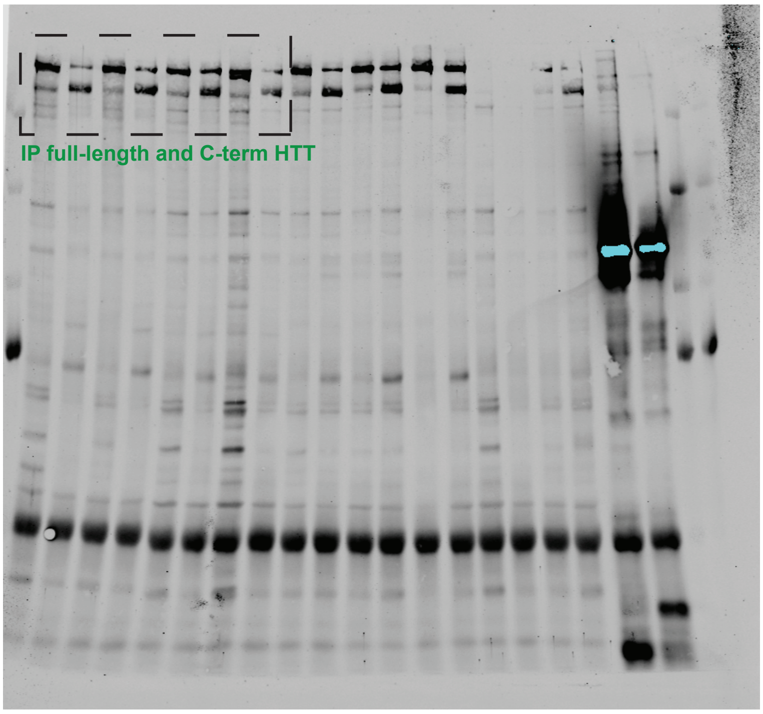

Supplement: Supplementary file 9 [file DataSheet1.PDF]
